# Hyperpolarization Effects in Parahydrogen Activation with Pnictogen Biradicaloids: Metal‐free PHIP and SABRE

**DOI:** 10.1002/cphc.202100141

**Published:** 2021-04-07

**Authors:** Vladimir V. Zhivonitko, Henrik Beer, Danila O. Zakharov, Jonas Bresien, Axel Schulz

**Affiliations:** ^1^ NMR Research Unit University of Oulu P.O. Box 3000 90014 Oulu Finland; ^2^ Institute of Chemistry University of Rostock Albert-Einstein-Strasse 3a 18059 Rostock Germany; ^3^ Leibniz-Institut für Katalyse e.V. Universität Rostock Albert-Einstein-Strasse 29a 18059 Rostock Germany

**Keywords:** biradicaloid, parahydrogen, NMR, hyperpolarization, metal-free activation

## Abstract

Biradicaloids attract attention as a novel class of reagents that can activate small molecules such as H_2_, ethylene and CO_2_. Herein, we study activation of parahydrogen (nuclear spin‐0 isomer of H_2_) by a number of 4‐ and 5‐membered pnictogen biradicaloids based on hetero‐cyclobutanediyl [X(μ‐NTer)_2_Z] and hetero‐cyclopentanediyl [X(μ‐NTer)_2_ZC(NDmp)] moieties (X,Z=P,As; Ter=2,6‐Mes_2_−C_6_H_3_, Dmp=2,6‐Me_2_−C_6_H_3_). The concerted mechanism of this reaction allowed observing strong nuclear spin hyperpolarization effects in ^1^H and ^31^P NMR experiments. Signal enhancements from two to four orders of magnitude were detected at 9.4 T depending on the structure. It is demonstrated that 4‐membered biradicaloids activate H_2_ reversibly, leading to SABRE (signal amplification by reversible exchange) hyperpolarization of biradicaloids themselves and their H_2_ adducts. In contrast, the 5‐membered counterparts demonstrate rather irreversible parahydrogen activation resulting in hyperpolarized H_2_ adducts only. Kinetic measurements provided parameters to support experimental observations.

## Graphical abstract

**Metal‐free parahydrogen activation** by a series of four‐ and five‐ membered pnictogen biradicaloids is reported. Strong ^1^H and ^31^P hyperpolarization effects induced by parahydrogen were observed and discussed. The symmetry of the activator biradicaloids strongly affected the shape and amplitude of the hyperpolarization signals. Signal enhancements of more than three orders of magnitude were detected at 9.4 T.

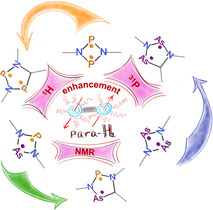

Biradicaloids receive considerable attention due to the unique reactivity that can be used to create new functional materials. For instance, recent applications include photoswitching,^[1]^ non‐linear optics,^[2]^ molecular magnets^[3]^ and activation of small molecules such as CO_2_, NH_3_ and also H_2_.^[4]^ Among other matters, activation of molecular hydrogen is interesting for parahydrogen‐based nuclear spin hyperpolarization methods such as PASADENA (parahydrogen and synthesis allows dramatically enhanced nuclear alignment),^[5]^ ALTADENA (adiabatic longitudinal transport after dissociation engenders net alignment)^[6]^ and SABRE.^[7]^ The hyperpolarization requires a chemical activation of parahydrogen, the spin‐0 nuclear spin isomer of H_2_.^[8]^ If the activation is pairwise, i. e., the two H atoms do not lose each other, a strong nuclear spin hyperpolarization and orders of magnitude NMR signal enhancements are observed due to the parahydrogen‐induced polarization (PHIP).[^8a^, ^9^] Typically, metal‐based catalysts such as various Rh, Ir or other metal complexes are used for this purpose.^[10]^ In principle, metal‐free catalysts/activators made of light biogenic elements can provide a new useful alternative to the traditional catalytic systems.

So far, a few metal‐free systems were demonstrated to produce nuclear spin hyperpolarization effects with parahydrogen (Scheme 1, **1**–**8**). These are mainly N−B intramolecular frustrated Lewis pairs (FLP) (**1**–**7**) such as ansa‐amino boranes,^[11]^ and there is also an example of FLP based C−P pair of aromatic triphosphabenzene **7**.^[12]^ Quite recently, a P−P biradicaloid **8** was demonstrated to show hyperpolarization effects both in ^1^H and ^31^P NMR spectra while activating parahydrogen molecules.^[13]^ The symmetry of **8**‐H_2_ adduct led to observation of unusual ^1^H and ^31^P signal multiplets with enhancements exceeding at least one order of magnitude at 7 Tesla.

**Scheme 1:**
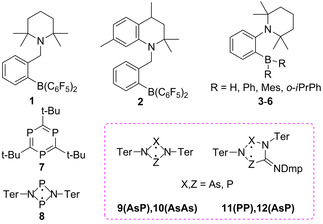
Metal‐free compounds known to activate parahydrogen and demonstrate nuclear spin hyperpolarization effects.

Herein, we study promising novel pnictogen (P,As) biradicaloids **9**–**12**, addressing the nuclear spin hyperpolarization effects observed upon activation of parahydrogen molecules. We show that all these biradicaloids can activate H_2_, which leads to strong hyperpolarization of biradicaloid‐parahydrogen adduct molecules. We demonstrate that H_2_ activation properties of four‐ and five‐membered biradicaloids are different since the former ones show facile reversible activation that leads to metal‐free SABRE of the biradicaloids themselves and incorporation of As atoms promotes the reversibility. Unusual hyperpolarization effects related to the biradicaloid structures were detected.

First, we studied nuclear spin hyperpolarization effects for the four‐membered biradicaloids **8**–**10** at room temperature. The experiments were performed by bubbling parahydrogen gas through biradicaloid solutions in a 9.4 T magnet, which was followed by a π/4‐pulse and NMR signal detection (see Supporting Information). The corresponding ^1^H NMR spectra are shown in Figure 1.

**Figure 1:**
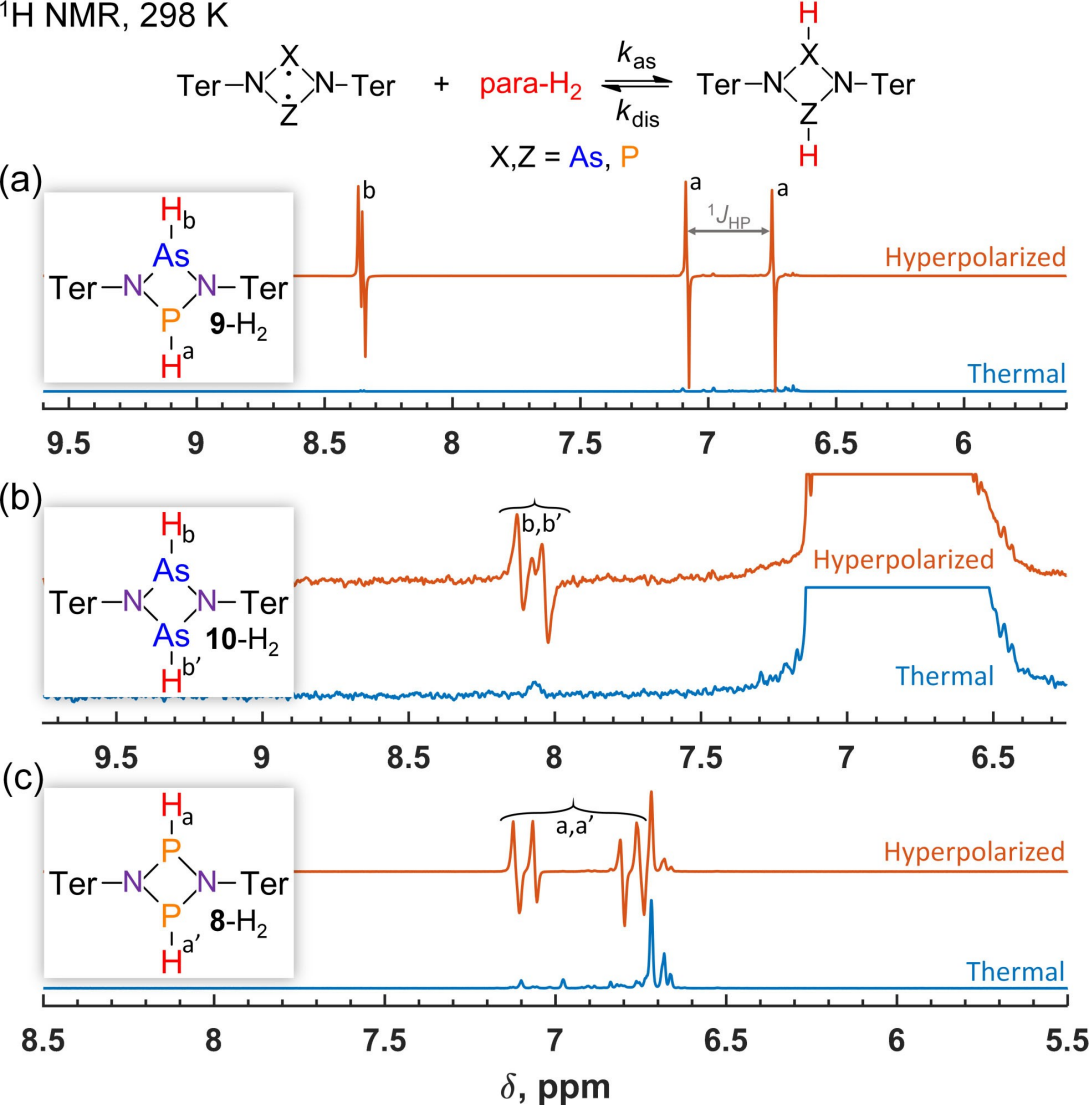
^1^H NMR spectra showing nuclear spin hyperpolarization effects observed at room temperature in the reversible interaction of parahydrogen with four‐membered biradicaloids **9** (a), **10** (b) and **8** (c). The red traces are acquired in first moments after introduction of parahydrogen to 0.04 M solutions of the biradicaloids in toluene‐d8. The blue traces show the thermal polarization level. The high‐intensity signals corresponding to thermal protons are off‐scale and clipped in (b) for a better visibility of other signals.

The asymmetrical As−P biradicaloid **9** demonstrated the most prominent hyperpolarization effect, leading to a substantial enhancement of P−H^a^ and As−H^b 1^H NMR signals of **9**‐H_2_ adduct. As the parahydrogen activation takes place at the high magnetic field, the antiphase signals are observed in this case, as it should be for a weakly coupled spin system according to PASADENA experiment in theory.[^8a^, ^9^] Due to the *J*‐coupled ^31^P, the enhanced antiphase resonances are split for the both H^a^ and H^b^ protons (H^a^: *dd* δ=6.91 ppm, ^4^ *J*_HH_=5.2 Hz, ^1^J_HP_=135.0 Hz; H^b^: *dd* δ=8.36 ppm, ^4^ *J*_HH_=5.2 Hz, ^3^ *J*_HP_=5.8 Hz). The measured enhancement factor ϵ is ca. 250 at 9.4 T and 298 K.

Hyperpolarization effects for the symmetrical As−As (**10**) and P−P (**8**) counterparts cannot be interpreted in a straightforward way as compared to **9** (Figure 1b and 1c). The activation products **10**‐H_2_ and **8**‐H_2_ form AA'XX’ spin systems (A=^1^H, X=^75^As or ^31^P), and this feature complicates corresponding NMR signal shapes significantly. Since the spin pairs of the same kind are strongly coupled, the resulting NMR spectra are expected to have complex signal structures due to the second‐order effects.^[14]^ The observed resonances correspond to superposition states of the spin pairs rather than to the individual spins.

Our measurements show that As−As derivative **10** has a clear central transition in the thermal ^1^H NMR spectrum, while the strongest hyperpolarization effects are observed at the side bands (Figure 1b). Since the side band transitions were invisible in the thermal spectrum due to their weak intensities, the precise measurement of signal enhancement is not possible. However, comparing the enhanced signals to the scaled noise level in the thermal spectrum with extensive scan accumulations, we estimated that the enhancement factor must be at least >10^4^ (Figure S1, see Supporting Information). Our attempts to simulate thermal and hyperpolarized spectra based on computed J‐couplings (see Supporting Information, Table S8) didn't provide good matching of the experiment and theory, most likely due to the quadrupolar nature of ^75^As. Indeed, among possible reasons for the discrepancy are errors in the computed parameters, presence of relatively fast chemical exchange that modulates J‐coupling constants and strong quadrupolar relaxation of ^75^As (spin‐3/2). The latter two sources should lead to shifts of spectral lines and to the scalar relaxation of the first and the second kinds, respectively.^[15]^ The detailed consideration of these mechanisms, however, is beyond the scope of this study and will be addressed in the future.

^1^H signal enhancements for **8** are more than one order of magnitude, as was also shown elsewhere.^[13]^ In principle, the signal enhancements in the symmetrical systems cannot be interpreted very easily in terms of conventional PASADENA theory in the weak coupling limit.^[8a]^ The signal enhancements are observed for “forbidden” transitions, which are not considered in that theory. At the same time, it is possible to use SLIC (Spin‐Lock Induced Crossing)^[16]^ to convert antiphase polarization to in‐phase, which was shown for **8** earlier.^[13]^

In the case of mixed As−P biradicaloid **9**, the substantial ^1^H polarization can be efficiently transferred to ^31^P nuclei. For instance, we utilized recently introduced ESOTHERIC (Efficient Spin Order Transfer via Relayed Inept Chains)^[17]^ to perform the polarization transfer (see Supporting Information). Figure 2a shows ^31^P NMR spectra at 308 K after the polarization transfer and after the relaxation to thermal equilibrium. The comparison reveals a ^31^P signal enhancement factor (ϵ) of 2000 at 9.4 T for **9**‐H_2_ adduct, demonstrating the high efficiency of this process. We should note that this is an unusually high signal enhancement for metal‐free parahydrogen activations, highlighting the high efficiency of this biradicaloid system. Moreover, the reversibility of H_2_ activation/addition by **9** opens a way for hyperpolarizing this molecule simply by waiting for **9**‐H_2_ to dissociate. Indeed, setting a 300 ms delay between polarization transfer pulse‐sequence and acquisition leads to the ^31^P NMR spectrum depicted in Figure 2b. It is clearly seen that in addition to **9**‐H_2_ the initial resonance of **9** becomes strongly enhanced with the factor of ca. 1000‐fold. The hyperpolarization of molecules without the actual chemical modification is the feature of SABRE technique. There is a natural difference between our observations and the original SABRE experiment,^[7]^ since we don't use metal complexes. However, the hyperpolarization of the initial biradicaloid molecule is facilitated by the reversible exchange and the polarization transfer is mediated through the J‐couplings. Therefore, we call this effect metal‐free SABRE. This is another example of metal‐free SABRE, complementing one reported for spontaneous ^15^N hyperpolarization with FLPs based on ansa‐aminoboranes.^[11c]^ However, the SABRE efficiency in the current work was better by at least two orders of magnitude.

**Figure 2:**
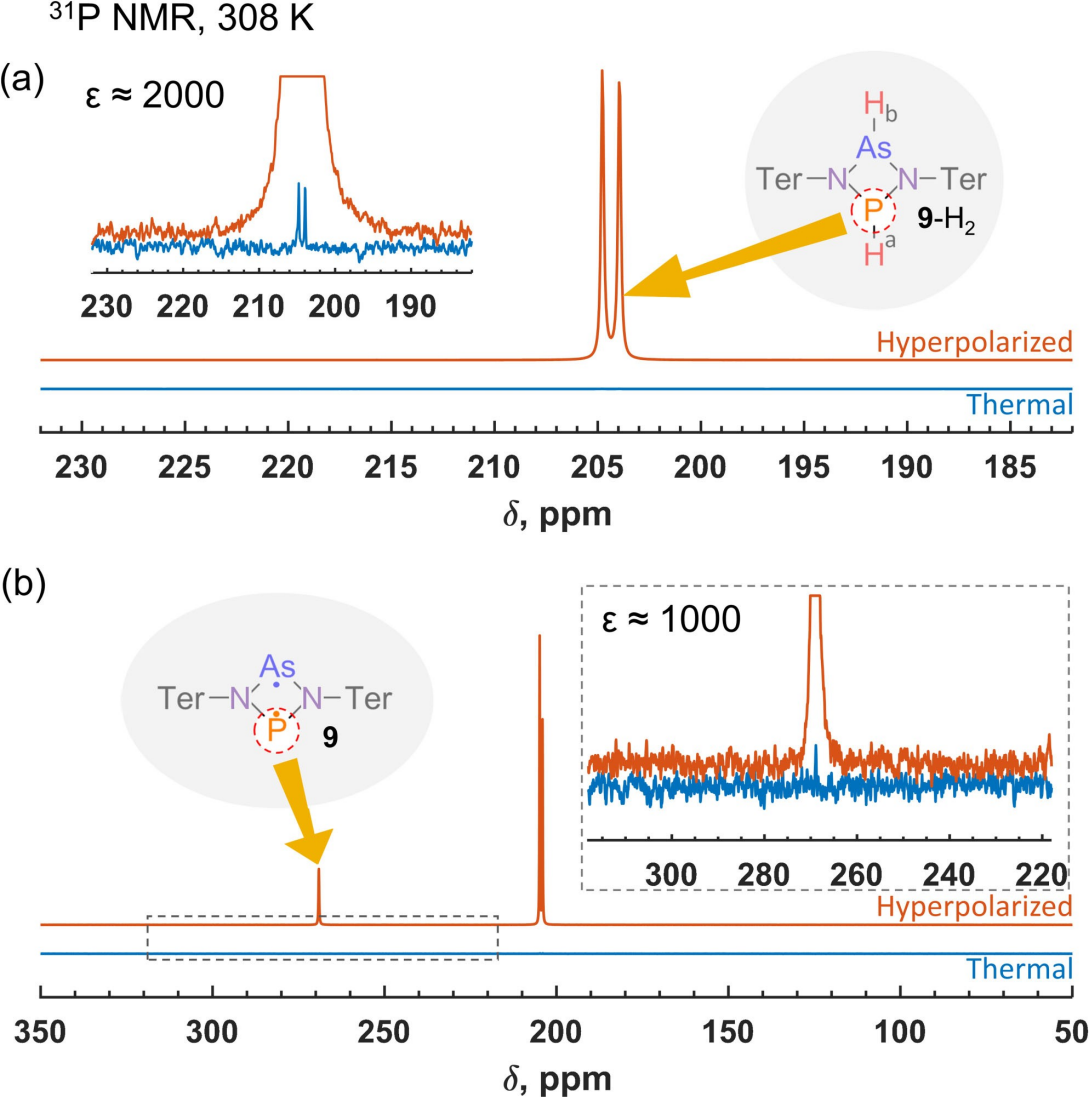
^31^P NMR spectra acquired immediately after the hyperpolarization transfer from ^1^H to ^31^P (a) and with a 300 ms mixing time delay (b). The insets show comparisons of the thermal (blue trace) and hyperpolarized (red trace) signals of **9**‐H_2_ (a) and **9** (b). In the insets, the strong hyperpolarization signals are off‐scale and clipped in.

In contrast to **9**, heteronuclear hyperpolarization with the symmetrical four membered bicadiacaloids **8** (^31^P) and **10** (^75^As) may not require the use of any polarization transfer pulse sequences. In both cases, the symmetry properties of the resulting AA'XX’, wherein A and A’ nuclei come from parahydrogen, allows inherent hyperpolarization of X nuclei. This feature was demonstrated for ^31^P in **8**‐H_2_ adduct,^[13]^ providing up to ca. 300‐fold signal enhancement. In this work, we attempted to observe the inherent hyperpolarization of ^75^As in **10**‐H_2_, but no visible enhancement was detected. Most likely, this is due to the destructive effect of the fast ^75^As relaxation leading to extremely broad ^75^As NMR signals and collapsing partially the multiplet structure.

An important feature of the four‐membered biradicaloids **8**–**10** is the reversibility of H_2_ addition under ambient temperatures, which leads to the dynamic exchange between bound and free H_2_ pools. This feature allows one to hyperpolarize the corresponding adducts constantly, provided that the continuous supply of parahydrogen is present. It should be noted that the reverse reaction rate increases with replacement of P atoms with As. For instance, at room temperature the dissociation of baradicaloid‐H_2_ adducts is characterized by the experimentally measured rate constants (*k*_dis_) 1×10^−4^, 0.15, 24 s^−1^ for **8**, **9** and **10**, correspondingly. The replacement of P with As leads to a clear gradual decrease of the activation energy (Table 1) which qualitatively correlates with computational data shown in Table S5 in Supporting Information. This is reasonable since bonds between hydrogen and atoms of the same main group weaken as we go down the group due to the decreased overlap integral between valence orbitals of the bonded atoms (cf. bond dissociation energies (BDE): BDE(As−H)=319.2(0.8) vs. BDE(P−H)=351.0(−2.1) kJ/mol).^[18]^

**Table 1:** Experimental enthalpies and entropies of dissociation of biradiacaloid‐H_2_ adducts

Compound	ΔH^≠^, [kcal/mol]	ΔS^≠^, [kcal/mol ⋅ K]
**8**‐H_2_^[a]^	21±2	−8±5
**9**‐H_2_	16±1	−8±3
**10**‐H_2_	13±2	−9±7

[a] Published in Ref. [13]

In contrast to the four‐membered biradicaloids, the five‐membered ones, **11** and **12**, bind H_2_ rather irreversibly. Moreover, strong ^1^H NMR hyperpolarization effects with parahydrogen were observed only for P−P derivative **11**. The corresponding ^1^H NMR spectrum is shown in Figure 3. As a result of the weak spin‐spin coupling between nuclei of two ^31^P^1^H pairs in **11**‐H_2_, the spectrum reveals a signal pattern that includes the system of eight antiphase doublets corresponding to protons “a” and “b” originating from parahydrogen (H^a^: *m* δ=4.20 ppm, ^4^ *J*_HH_=2.5 Hz, ^1^ *J*_HP_=169.7 Hz, ^3^J_HP_=4.1 Hz; H^b^: *m* δ=5.08 ppm, ^4^ *J*_HH_=2.5 Hz, ^1^J_HP_=202.2 Hz, ^3^J_HP_=9.6 Hz).

**Figure 3:**
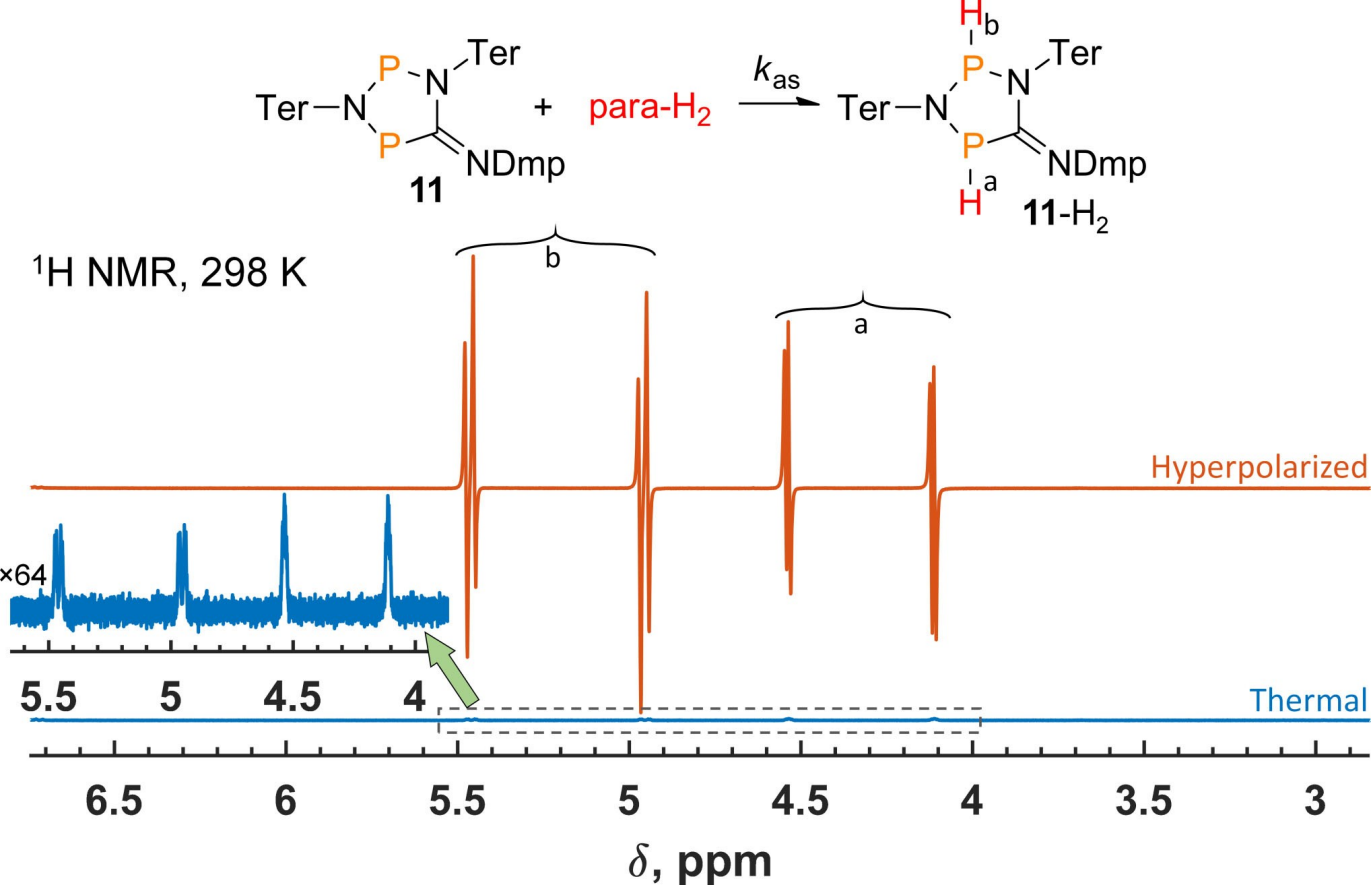
^1^H NMR spectra acquired at the first moment after bubbling parahydrogen (red trace, top) through 0.04 M solution of **11** and after the relaxation to thermal equilibrium (blue trace, bottom) at room temperature. The inset shows the level of thermal signals multiplied by a factor of 64.

Due to the reaction irreversibility, the hyperpolarization effects were observed only at the first moments after parahydrogen bubbling for adduct **11**‐H_2_. The direct comparison of the resulting signal amplitudes with those in thermal spectra shows ca. 120‐fold signal enhancement. We should note, however, that the H−H spin coupling in the adduct is only about 2.5 Hz whereas the linewidth is ca. 2 Hz, which led to a significant mutual cancellation of the signal components of the antiphase doublets. The lineshape fitting predicted at least 250‐fold enhancement. Moreover, the determination of the exact enhancement factor through comparison to the thermal spectra is not a straightforward procedure under such experimental conditions because of the continuous accumulation of the adduct **11**‐H_2_. Therefore, 250 is the lowest possible estimate. The ^1^H hyperpolarization was easy to transfer to ^31^P nuclei via coherent polarization transfer with ESOTHERIC, providing 130‐fold signal enhancements for both phosphorus centers in **11**‐H_2_ (see Supporting Information, Figure S3).

The reaction of **12** with parahydrogen at room temperature resulted in observation of thermally polarized signals of **12**‐H_2_ in ^1^H NMR spectra (see Supporting Information, Figure S6), meaning that this process does not lead to the observable polarization effects. The mechanism of parahydrogen activation with this biradicaloid most likely is still pairwise, but H atoms originating from parahydrogen are not coupled strongly enough by J‐coupling. In this circumstance, the parahydrogen nascent nuclear spin order cannot be efficiently converted into the observable magnetization, thus effectively producing no hyperpolarization effects. The corresponding J‐coupling constant of ≤1 Hz estimated by analysis of experimental thermal spectra supports this conclusion.

Interestingly, weak hyperpolarization effects in ^1^H and ^31^P NMR spectra of **12**‐H_2_ were nevertheless observed by heating its equilibrium solution in the presence of parahydrogen. For instance, in‐phase doublet of **12**‐H_2_ in ^31^P NMR spectrum was converted into antiphase doublet at 334 K, indicating the weak but still detectable reversibility of the H_2_ activation by **12** (see Figure S7 and corresponding discussion in Supporting Information). Similar effect was observed also by heating **11**‐H_2_ but at a higher temperature (373 K) (Figure S5 in Supporting Information), implying that activation H_2_ by **11** also shows signs of reversibility. Attempts to synthesize the five‐membered As−As analogue of **11** and **12** were not successful, since the initial four membered biradicaloid did not react with 2,6‐dimethylphenyl‐isonitrile to give the desired product.

Herein, we considerably expanded the range of metal‐free biradicaloids that demonstrate hyperpolarization effects upon interaction with parahydrogen. The strongest hyperpolarization effects are observed for As−P four‐membered compound **9** in both ^1^H and ^31^P NMR spectra. The experimentally measured signal enhancements exceed three orders of magnitude at 9.4 T, i. e., correspond to at least 3 % spin polarization for both nuclei. To date, this is a record high signal enhancement that was observed using metal‐free parahydrogen activation. Comparing to much more widely explored metal‐free activation with ansa‐aminoboranes,^[11]^ it provides an order of magnitude improvement of the observed hyperpolarization. The reversibility of activation was used to hyperpolarize biradicaloids themselves via a high‐field SABRE effect, providing another example of SABRE in metal‐free systems in addition to ansa‐aminoboranes.^[11c]^ Symmetrical P−P and As−As four‐membered biradicaloid adducts (**8**‐H_2_ and **10**‐H_2_) form symmetrical AA'XX’ spin systems (A=^1^H, X=P,As) resulting in complex NMR multiplets having features of second‐order spectra.^[14]^ This can be viewed as a consequence of chemical equivalence of ^1^H and pnictogen spin pairs at the simultaneous magnetic inequivalence of spins in these pairs. This allows one to observe unusual effects such as hyperpolarization of ^31^P without a need for polarization transfer radio‐frequency pulses for **8**‐H_2_ or strongly enhanced ^1^H side band resonances in the case of **10**‐H_2_. Among five‐membered biradicaloids, P−P based **11** provided strong signal enhancements of at least 250‐fold, and all studied members of this class are shown to activate H_2_ rather irreversibly, as compared to four‐membered biradicaloids. However, the experiments with **11**‐H_2_ and **12**‐H_2_ performed at high temperatures (334 and 373 K) under parahydrogen pressure showed slight hyperpolarization of these adducts, indicating a weak reversibility of H_2_ activation that was practically impossible to detect without the hyperpolarization. Altogether, we demonstrated versatile possibilities of hyperpolarization using metal‐free pnictogen biradicaloids. Immediately, these biradicaloids cannot be used for NMR sensitivity enhancement *in vivo* because of air‐sensitivity and toxicity of As. So far, they were not shown to mediate hydrogenation reactions, either. However, we would expect that a rational design of biradicaloids for parahydrogen activation may lead to applications in these areas in the future.

## Experimental Section

Biradicaloids **8**–**12** were synthesized using modifications of synthetic approaches described elsewhere,[^1^, ^19^] and detailed in Supporting information. The experiments with parahydrogen were performed by using 5 mm gas‐tight NMR tubes containing ca. 0.04 M solutions of biradicaloids in toluene‐d8 under elevated pressure of parahydrogen (92 % enrichment). Dissociation constants were measured using saturation transfer method similarly as described in Ref. [11b, 13] Other essential details are presented in Supporting Information.

## Conflict of interest

The authors declare no conflict of interest.

## Supporting information

As a service to our authors and readers, this journal provides supporting information supplied by the authors. Such materials are peer reviewed and may be re‐organized for online delivery, but are not copy‐edited or typeset. Technical support issues arising from supporting information (other than missing files) should be addressed to the authors.

Supplementary
