# Supplementary material for: Hyperpolarization Effects in Parahydrogen Activation with Pnictogen Biradicaloids: Metal‐free PHIP and SABRE
Source: Chemphyschem. 2021 Apr 7;22(9):813–7. doi: 10.1002/cphc.202100141 (PMC8251785; doi:10.1002/cphc.202100141)
Supplement: Supplementary file 1 — Supplementary [file CPHC-22-813-s001.pdf]

ChemPhysChem

Supporting Information

# Hyperpolarization Effects in Parahydrogen Activation with Pnictogen Biradicaloids: Metal-free PHIP and SABRE

Vladimir V. Zhivonitko,* Henrik Beer, Danila O. Zakharov, Jonas Bresien, and Axel Schulz

-CONTENTS-

1 Parahydrogen experiments ..... 2
1.1 Comparison of $^{1}H$ NMR spectra of hyperpolarized and thermally polarized 4-membered As-As biradicaloid adduct 10-$H_2$ ..... 3
1.2 ESOTHERIC polarization transfer to $^{31}P$ ..... 4
1.3 Reversibility of parahydrogen activation by 5-membred biradicaloids 11 and 12 at high temperature ..... 6
1.4 Kinetic measurements of biradicaloid-$H_2$ dissociation ..... 10
2 Synthetic procedures ..... 12
2.1 Synthesis of starting materials ..... 14
2.2 Synthesis of target biradicaloids ..... 33
3 Computational Details ..... 46
3.1 Summary of calculated data ..... 47
3.2 Optimized structures (.xyz files) ..... 51
4 References ..... 84

# 1 Parahydrogen experiments

General information. NMR experiments with parahydrogen were performed on a 400 MHz Bruker AV 400 NMR spectrometer equipped with a broad-band 5 mm RF probe. The standard temperature control unit of the NMR spectrometer was used for cooling and heating samples. Kinetic measurements were performed using the same spectrometer. Parahydrogen-enriched $H_2$ gas (92%) referred to in the main text as simply parahydrogen was produced with a Bruker parahydrogen generator.

Experimental procedure. In a typical workflow, parahydrogen was bubbled through a 0.04 M solution of the biradicaloids in degassed dry toluene-$d_8$ (Sigma Aldrich) in a 5 mm sample inside the NMR magnet for ca. 5 s, and then the parahydrogen flow was abruptly switched off and an NMR experiment was started. Typically, $\pi/4$-pulses were used to maximize observed effects. The bubbling procedure was performed under a 4 bar parahydrogen pressure in the same manner as explained in detail in Ref.[S1] Parahydrogen was supplied to the bottom of the sample tube through a 1/32” PTFE tubing. The sample temperature was varied in the experiments when it was required. In some test experiments, a 5 mm gas tight sample tube was charged with 6 bar of parahydrogen and shaken just before inserting to the NMR spectrometer for analysis. Both schemes provided similar results in the case of symmetrical biradicaloids indicating that the PASADENA conditions are effective in both cases, i.e. the influence of the magnetic field variation on the signal shape was negligible.

Since investigated biradicaloids are highly sensitive to both air and moisture, the sample preparation procedures were done under inert Ar atmosphere.

## 1.1 Comparison of $^{1}H$ NMR spectra of hyperpolarized and thermally polarized 4-membered As-As biradicaloid adduct 10-$H_2$

A $^{1}H$ NMR spectrum acquired after parahydrogen bubbling at 278 K through the 0.04 M solution of [As(μ-NTer)]$_2$ (10) in toluene-$d_8$ is shown in Figure S1a. For comparison, a thermal equilibrium spectrum after the relaxation is shown in Figure S1b. The lower temperature provided both stronger hyperpolarized and thermal signals. The detection of unusual antiphase signals after the parahydrogen bubbling serves as a solid justification for the formation of the hyperpolarized state. Moreover, it is clear that hyperpolarized signals at the side bands from the central peak demonstrate a very strong signal enhancement (>$10^4$ fold according to our estimations), since no sign of thermal signals were detected even after very long time (44 h) of 18432 scan accumulations.

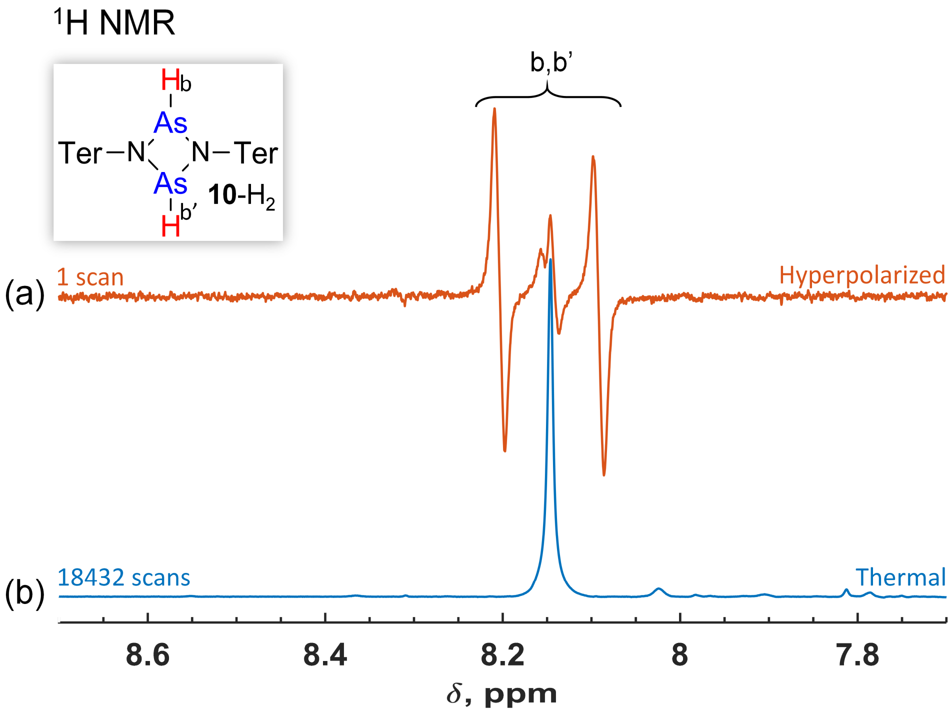


Figure S1. $^{1}H$ NMR spectra obtained after parahydrogen bubbling through a 0.04 M toluene-$d_8$ solution of 10, [As(μ-NTer)]$_2$, with 1 scan accumulation (a) and after the relaxation to thermal equilibrium with 18432 scan accumulations (b) at 278 K. As indicated using structure drawing, the signals corresponding to $H_2$ originating protons in 10-$H_2$ are shown in the figure. Both spectra were acquired using π/4-pulses. No signs of thermal signals at side bands were detected in the thermal spectrum.

## 1.2 ESOTHERIC polarization transfer to $^{31}P$

The following pulse sequence was employed to perform polarization transfer from antiphase $^{1}H$ nuclear spin order originating from parahydrogen to $^{31}P$ using ESOTHERIC (Efficient Spin Order Transfer via Relayed Inept Chains)[S2,3] approach.

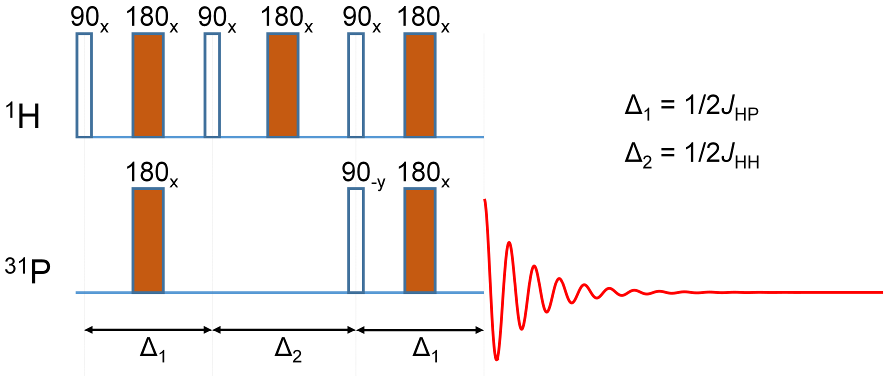


Figure S2. Pulse sequence employed to perform polarization transfer to $^{31}P$ using ESOTHERIC approach.

This pulse sequence was successfully employed in experiments with biradicaloids 9 and 11. The parameters of the pulse sequence are shown in the Table S1.

Table S1. Parameters of ESOTHERIC experiment for different biradicaloids.

| Biradicaloid | $\Delta_1$, ms | $\Delta_2$, ms | $J_{HP}$, Hz | $J_{HH}$, Hz |
|---|---|---|---|---|
| 9 | 2.46 | 185.18 | 136.5 | 5.3 |
| 11 | 3.66 | 95.97 | 203.0 | 2.7 |

A measured ESOTHERIC spectrum for biradicaloid-parahydrogen adduct 11-$H_2$ is shown in Figure S3.

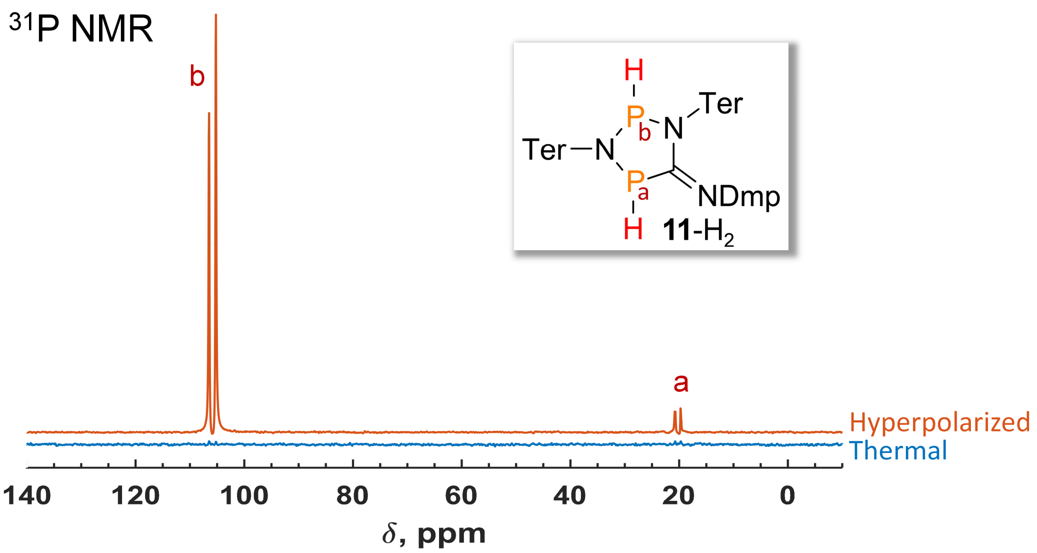


Figure S3. $^{31}$P NMR spectra obtained after a 3 s parahydrogen bubbling through a 0.04 M toluene-$d_8$ solution of 11 followed by ESOTHERIC polarization transfer (red trace) and after the relaxation to thermal equilibrium followed by π/2-pulse (blue trace) at 293 K.

Similar spectrum for biradicaloid-parahydrogen adduct 9-$H_2$ is shown in Figure 2a of the main text. Figure 2b shows also ESOTHERIC experiment with mixing time included to demonstrate SABRE effect on the initial biradicaloid 9. The same sequence parameters (Table S1) were used in the latter experiment, except of addition mixing delay ($\Delta_{mix}$ = 300 ms) inserted as shown in Figure S4 below.

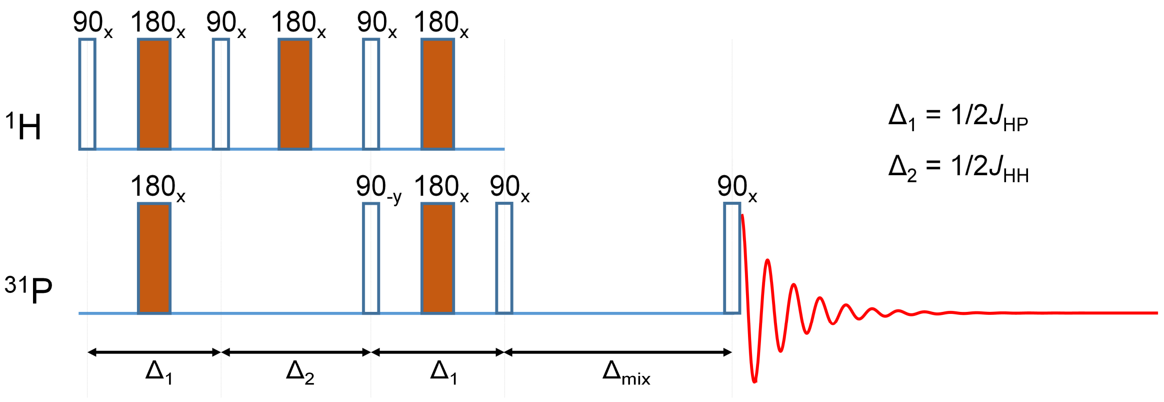


Figure S4. A modified pulse sequence employed to perform polarization transfer to $^{31}$P using ESOTHERIC approach followed by storage of the $^{31}$P magnetization during $\Delta_{mix}$ before the signal acquisition.

## 1.3 Reversibility of parahydrogen activation by 5-membred biradicaloids 11 and 12 at high temperature

Parahydrogen activation by 5-membered biradicaloids 11 and 12 at room temperature is rather irreversible process since no hyperpolarization effects were visible after first moments of bubbling the gas. In the simplest case, the reversibility can be considered as the following overall equilibrium process that in principle can lead to a continuous hyperpolarization of the resulting biradicaloid adduct.

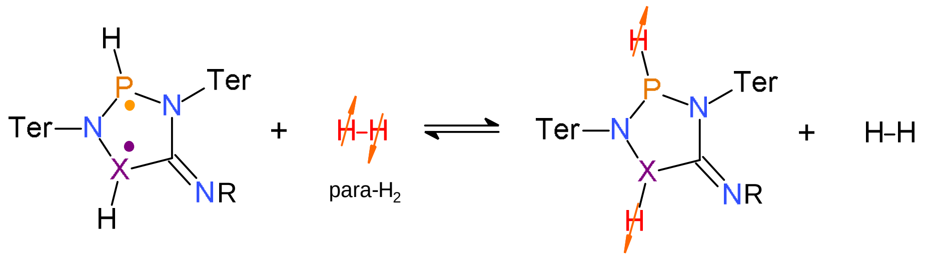


X = P(**11**),As(**12**)

An increase in temperature up to 334 K did not improve the situation with the inefficient reversibility dramatically. However, we observed clear signs of some reversibility of the activation process, which were especially clear in $^{31}$P NMR spectra.

For biradicaloid 11, heating the reaction mixture inside the NMR magnet resulted in the following effects in $^{31}$P NMR spectra (Figure S5).

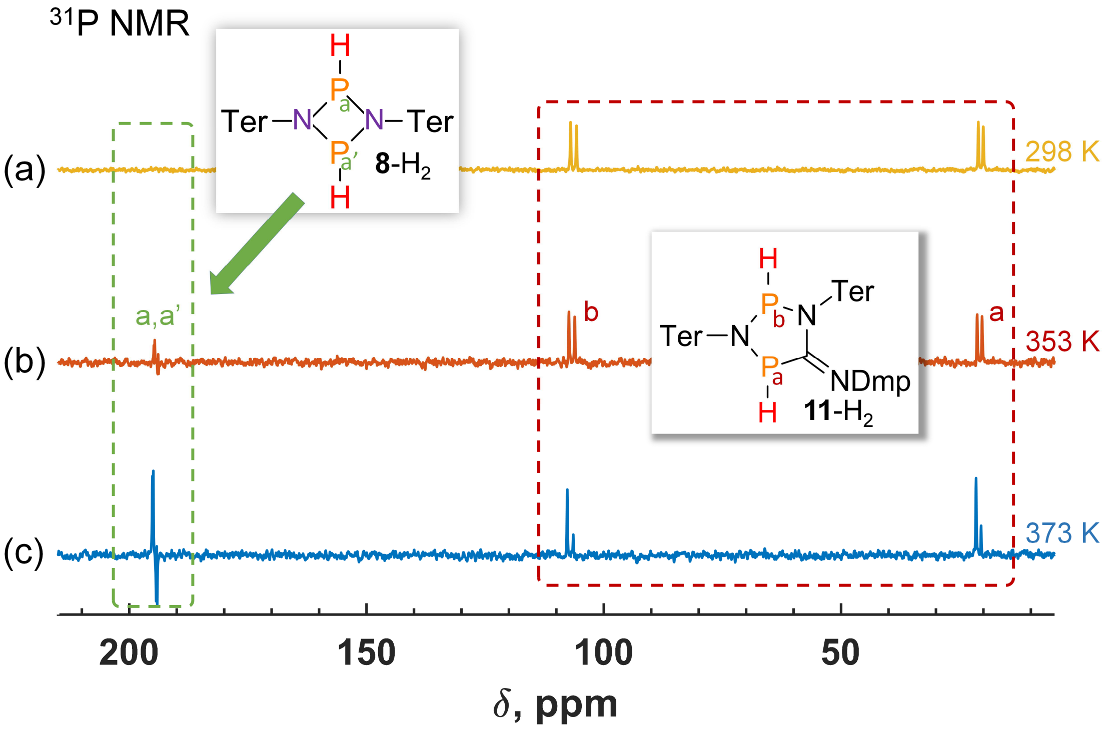


Figure S5. $^{31}P$ NMR spectra acquired after adding parahydrogen into an equilibrium solution (ca. 0.04 M) of 11-$H_2$ adduct at 298 (a), 353 (b) and 373 K (c). The signals corresponding to 8-$H_2$ and 11-$H_2$ are shown with letters from the presented structures in the figure.

It was clear that the increase of the temperature (298→353→373 K) leads to two hyperpolarization effects. The first one is observation of hyperpolarized adduct of 4-membered biradicaloid 8 and parahydrogen. This compound could present in trace amounts after synthesis of biradicaloid 11 or due to slight dissociation of 11 into 8 and 2,6-dimethylphenyl-isonitrile. The second effect is deformation of doublet $^{31}P$ NMR signals corresponding to 11-$H_2$. The higher the temperature, the stronger difference in the amplitude of the individual components of the two doublets. A comparison of spectra in Figure S5a and S5b shows the clear contrast. This deformation directly indicates the admixture of antiphase nuclear spin order that can originate only from parahydrogen, providing an evidence that with the temperature increase the reversibility of $H_2$ activation by 5-membered biradicaloid 11 becomes evident. At the same time, the strength of this effect is not high, and the rate of 11-$H_2$ dissociating is low. Indeed, we could not detect any exchange using the standard methods of exchange NMR spectroscopy and CEST using thermally polarized signals, highlighting

the beneficial role the hyperpolarization in understanding the reversibility of the activation.

In the case of experiments with biradicaloid 12, we could not observe any hyperpolarization after introduction of parahydrogen into a 0.04 M solution of 12 at 298 K (Figure S6). The adduct 12-$H_2$, however, was clearly produced as the result of the reaction. $^1$H NMR signals of this adduct are shown in the inset of Figure S6, and constitute two doublet signals (“a” and “b”), corresponding to the pair of parahydrogen originating protons. The hyperpolarization was not observed, most likely, because of a very small J-coupling constant between these protons in 12-$H_2$. At the same time, we could observe hyperpolarization effects stem from traces of 4-membered biradicaloid 9 (compare to the main text). This compound, likely, remained after synthesis of 12 in a very small amount but the high hyperpolarization allowed to observe adduct 9-$H_2$.

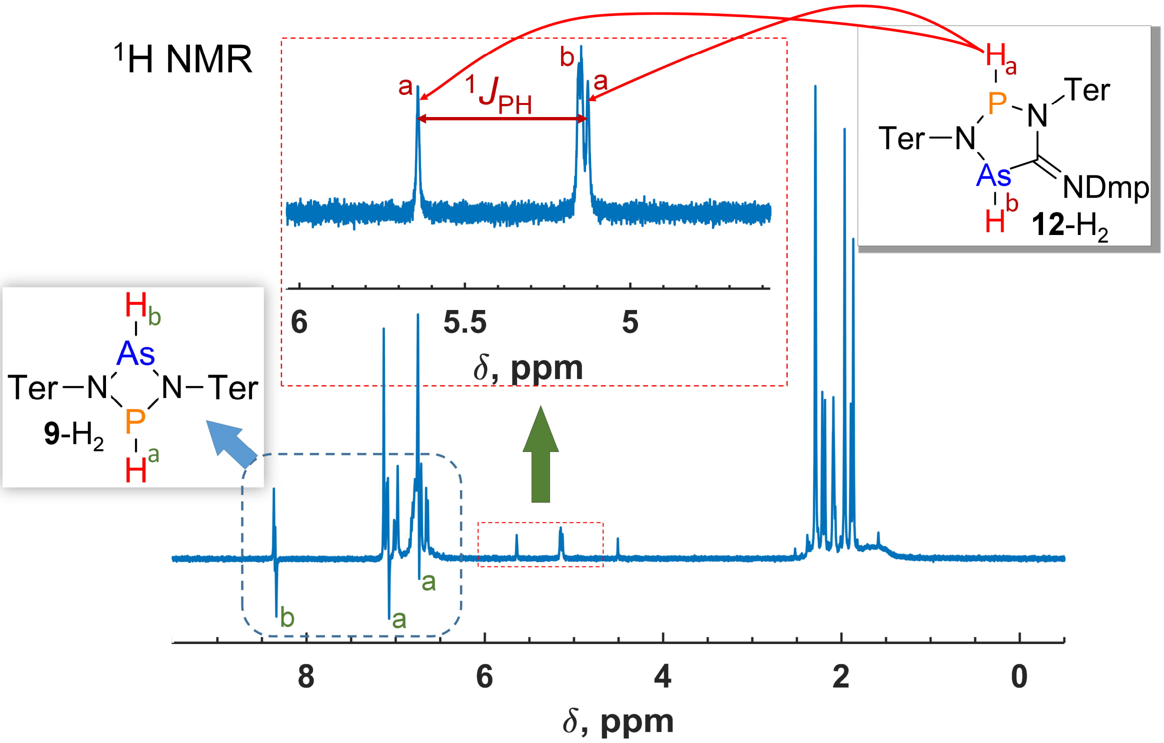


Figure S6. $^1$H NMR spectrum recorded after parahydrogen bubbling through ca. 0.04 M solution of 5-membered biradicaloid 12 at 298 K in a 9.4 T NMR magnet. No hyperpolarization effects visible at this temperature for the corresponding parahydrogen adduct 12-$H_2$. At the same time, hyperpolarized signals were observed for the parahydrogen adduct of 4-membred biradicaloid 9-$H_2$ produced from traces of 9 that remained after synthesis of 12. The labels of the NMR signals are shown using the shown structures.

We found that similarly to 5-membered biradicaloid adduct 11-$H_2$ heating the equilibrium solution of 12-$H_2$ to higher temperatures in the presence of parahydrogen led to a slight hyperpolarization of this compound. The most clearly it was visible in $^{31}$P NMR spectra showing a transformation of the doublet signal from in-phase to antiphase form upon heating from 298 to 334 K (Figure S7).

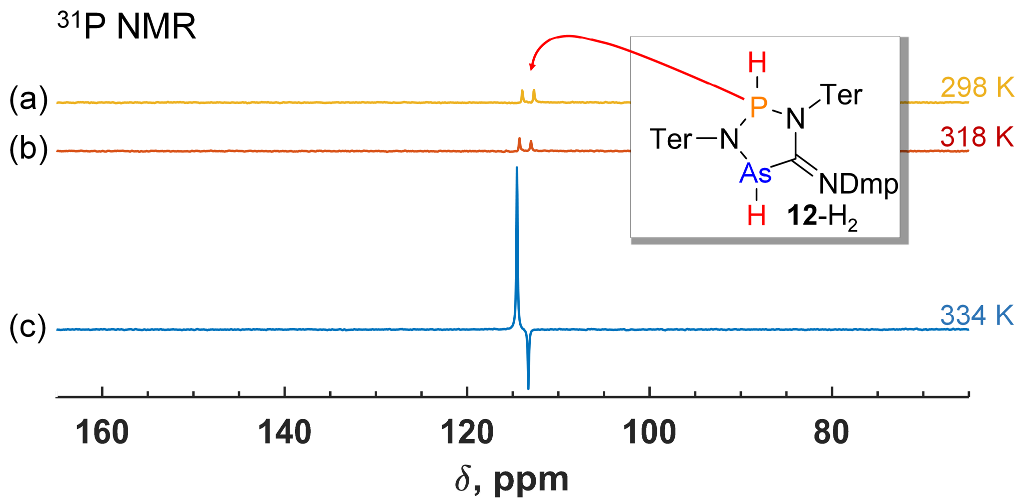


Figure S7. $^{31}$P NMR spectra acquired after introduction of parahydrogen into an equilibrium solution (ca. 0.04 M) of 12-$H_2$ adduct at 298 (a), 318 (b) and 334 K (c). The signal corresponding to 12-$H_2$ is shown with a red arrow.

This observation was reproduced always when parahydrogen enriched $H_2$ was present in the solution, implying that the bound hydrogens in 12-$H_2$ can reversibly exchange with parahydrogen in solution. This observation also indicates the reversibility that otherwise would be difficult to observe with thermally polarized signals.

There was a clear difference in temperature that was required to start observing the reversible exchange effects for the 5-membered adducts 11-$H_2$ and 12-$H_2$. The As derivative 12-$H_2$ allowed observing the effects at lower temperatures as compared to complete P one 11-$H_2$. The same trend of facile dissociation upon introduction of As atoms into the 4-membered biradicaloid structure was present (see main text for this discussion).

We should note that the observed high temperature hyperpolarization effects in $^{31}P$ NMR spectra cannot be explained in terms of simple PASADENA[S4] experiments. In both adducts, 11-$H_2$ and 12-$H_2$, parahydrogen originating atoms form a weakly coupled spin system which makes spontaneous polarization transfer to $^{31}P$ via coherent interactions be inefficient in contrast to, for instance, AA'XX' spin system of 8-$H_2$ adduct.[S5] The hyperpolarization of $^{31}P$ the 5-membered adducts must be built up due to the incoherent relaxation transitions that can create different magnetization modes between spin pairs interacting via dipole-dipole interaction and having chemical shift anisotropy.[S6] Similar mechanisms lead to spontaneous hyperpolarization of $^{15}N$ nuclei in ansa-aminoboranes upon metal-free activation of parahydrogen.[S7] A very thorough discussion of this mechanism is out of scope of this publication and will be addressed in the possible future studies.

## 1.4 Kinetic measurements of biradicaloid-$H_2$ dissociation

The measurements of kinetic constants were performed using spin saturation transfer method[S8] on a Bruker Avance III 400 MHz spectrometer. A heavy-wall 5 mm NMR tube equipped with a tight plug was used in the experiments. The sample was prepared by charging the tube with biradicaloid solutions (~0.04 M) and 5 bar of normal (thermal) $H_2$. $^1H$ decoupling (WALTZ16) was used for all acquisitions. The thermally polarized $^{31}P$ signal of 9-H2 (ca. 204.1 ppm) was used to determine the rate constants for the dissociation of 9-$H_2$ adduct into 9 and $H_2$ ($k_{dis}$), whereas thermally polarized $^1H$ signal of 10-$H_2$ (ca. 8 ppm) was used to determine the rate constants for the dissociation of $H_2$ adduct 10-$H_2$ into 10 and $H_2$ ($k_{dis}$). The constants were calculated from the ratios of the signals in the normal spectra and in the spectra acquired with presaturation of either 9 $^{31}P$ resonance at ca. 268.5 ppm and $H_2$ $^1H$ resonance at ca. 4.6 ppm, respectively. The resonances were presaturated in a continuous wave mode for 10 s. To determine $k_{dis}$, the following expression was used: $k_{dis} = (S_n/S_s – 1)/T_1$, in which $S_n$ is the signal intensity in the normal spectrum, $S_s$ is the signal intensity in the spectrum

with presaturation, and $T_1$ (ca. 1 s) is the relaxation time. The corresponding Eyring plots are shown below.

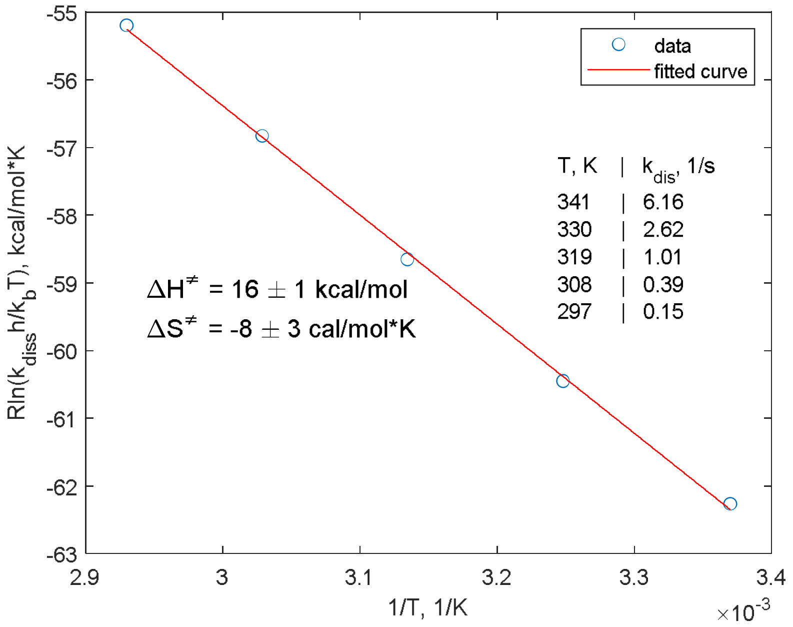


Figure S8. Eyring plot for the 9-$H_2$ dissociation process. Experimental points are drawn with circles and the linear fitting result is shown with a red line. Experimentally measured rate constants and the activation parameters obtained from the fitting at 95% confidence level are also shown in the graph.

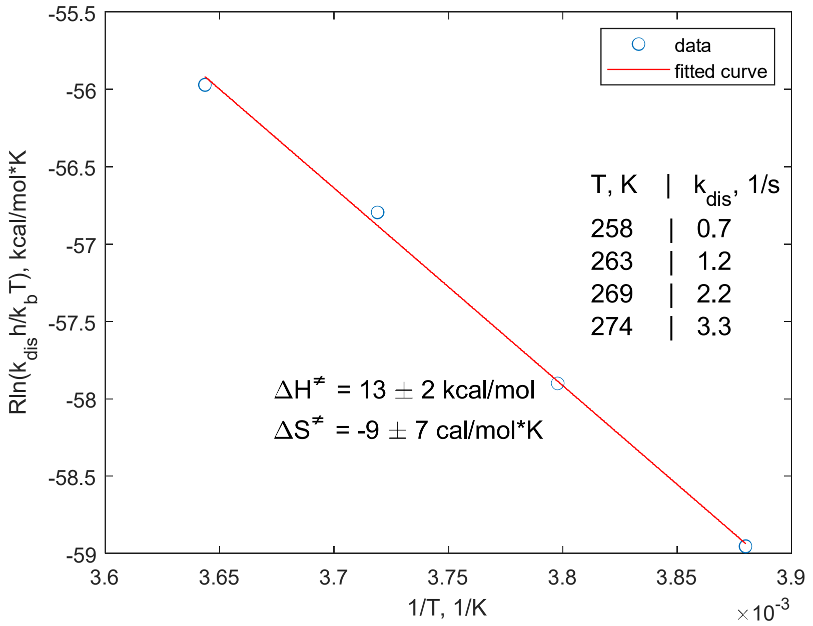


Figure S9. Eyring plot for the 10-$H_2$ dissociation process. Experimental points are drawn with circles and the linear fitting result is shown with a red line. Experimentally measured rate constants and the activation parameters obtained from the fitting at 95% confidence level are also shown in the graph.

# 2 Synthetic procedures

General Information. If not stated otherwise, all manipulations were carried out under oxygen- and moisture-free conditions under an inert atmosphere of argon using standard Schlenk or Drybox techniques. All glassware was heated three times in vacuo using a heat gun and cooled under argon atmosphere. Solvents were transferred using syringes, which were purged three times with argon prior to use. Solvents and reactants were either obtained from commercial sources or synthesized as detailed in Table S2.

Table S2. Origin and purification of solvents and reactants.

| Substance | Origin | Purification |
|---|---|---|
| $CH_2Cl_2$ | local trade | purified according to literature procedure[S9]<br>dried over $P_4O_{10}$, stored over $CaH_2$<br>freshly distilled and degassed (freeze-pump-thaw) |
| THF, $Et_2O$ | local trade | dried over Na/benzophenone<br>freshly distilled prior to use |
| $CD_2Cl_2$ | euriso-top | dried over $P_4O_{10}$ and $CaH_2$<br>freshly distilled prior to use |
| $C_6D_6$ | euriso-top | dried over Na<br>freshly distilled prior to use |
| $NEt_3$ | Sigma Aldrich, 99% | dried over Na<br>freshly distilled prior to use |
| Mg | abcr, 99.8%, for Grignards | can be activated by stirring for several days under argon using a glass covered magnetic stir bar. |
| $POCl_3$ | old stock | dried over $P_4O_{10}$<br>freshly distilled and degassed (freeze-pump-thaw) |
| $DmpNH_2$ | Arcos Organics, 99 % | freshly distilled |
| $TerNH_2$ | synthesized[S10] | re-crystallized as described in the literature |
| $TerN(H)PCl_2$ | synthesized[S10] | re-crystallized as described in the literature |
| Acetic Acid | old stock | used as recieved |
| $NaHCO_3$ | Baker | used as recieved |
| Thionylchlorid | Acros | used as recieved |
| Benzyl potassium (BzK) | synthesized[S11] | re-crystallized |
| n-BuLi | Sigma Aldrich | 2.5 M in hexane<br>used as received |
| $[P(\mu\text{-NTer})]_2$ (8) | synthesized[S10] | re-crystallized as described in the literature |
| $[As(\mu\text{-NTer})]_2$ (10) | old stock[S12] | re-crystallized[S12] |

NMR spectra were obtained on Bruker spectrometers AVANCE 250, 300 or 500 and were referenced internally to the signals of deuterated solvents ($^{13}C$: $CD_2Cl_2$ $\delta_{ref}$ = 54.0 ppm, $C_6D_6$ $\delta_{ref}$ = 128.4 ppm), to the signals of protic species in the deuterated solvents ($^{1}H$: $CHDCl_2$ $\delta_{ref}$ = 5.32 ppm, $C_6HD_5$ $\delta_{ref}$ = 7.16 ppm) or externally ($^{31}P$: 85 % $H_3PO_4$ $\delta_{ref}$ = 0 ppm). All measurements were carried out at ambient temperature unless denoted otherwise. NMR signals were assigned using experimental data (e.g. chemical shifts, coupling constants, integrals where applicable) in conjunction with computed NMR data (GIAO method, cf. Computational Details, p. S46).

IR spectra of crystalline samples were recorded on a Bruker Alpha II FT-IR spectrometer equipped with an ATR unit at ambient temperature under argon atmosphere. Relative intensities are reported according to the following intervals: very weak (vw, 0–10%), weak (w, 10–30%), medium (m, 30–60%), strong (s, 60–90%), very strong (vs, 90–100%).

Raman spectra of crystalline samples were recorded using a LabRAM HR 800 Horiba Jobin YVON Raman spectrometer equipped with an Olympus BX41 microscope with variable lenses. The samples were excited by a red laser (633 nm, 17 mW, air-cooled HeNe laser). All measurements were carried out at ambient temperature unless stated otherwise.

Elemental analyses were obtained using an Elementar vario Micro cube CHNS analyser.

Melting points (uncorrected) were determined using a Stanford Research Systems EZ Melt at a heating rate of 10 °C/min. Clearing points are reported.

Mass spectra were recorded on a Thermo Electron MAT 95-XP sector field mass spectrometer using crystalline samples.

UV-Vis spectra were acquired on a Perkin-Elmer Lambda 19 UV-Vis spectrometer.

## 2.1 Synthesis of starting materials

### 2.1.1 DmpN(H)COH

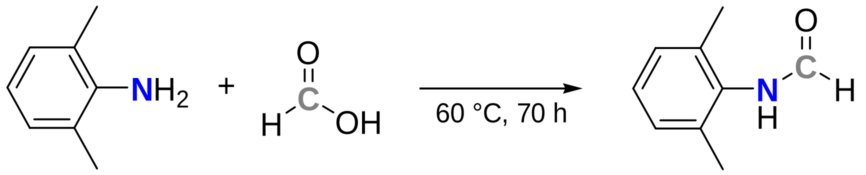


DmpN(H)COH was synthesized according to our previously published synthesis procedure.[S13] The synthesis was carried out under non-inert conditions.

2,6-Dimethylaniline (5.030 g, 41.51 mmol) was dissolved in formic acid (5.040 g, 109.5 mmol). The solution was degassed by several freeze-pump-thaw cycles. Afterwards the reaction mixture was stored at 60 °C for 70 hours (drying oven) resulting in white needles. The crude product was washed three times with water (25 mL) and dried in vacuo at 100 °C ($1\times10^{-3}$ mbar, oil bath). Yield: 5.423 g (36.34 mmol, 88%).

$C_9H_{11}NO$ (149.19 g/mol). Mp. 169 °C. CHN calcd. (found) in %: C 72.46 (72.22), H 7.43 (7.33), N 9.39 (9.33). $^1$H NMR ($CD_2Cl_2$, 250.1 MHz): $\delta$ = 2.22 (s, 6 H, trans-o-$CH_3$), 2.30 (s, 6 H, cis-o-$CH_3$), 7.08-7.09 (m, 3 H, cis/trans-m/p-CH), 7.09 (s, 2 H, trans-m-CH), 7.12-13 (m, 3 H, cis/trans-m/p-CH), 8.06 (d, $^3J(^1H,^1H)$ = 12 Hz, 1 H, NH), 8.30 (d, $^3J(^1H,^1H)$= 12 Hz, 1 H, CHO). $^{13}C\{^1H\}$ NMR ($CD_2Cl_2$, 62.9 MHz): $\delta$ = 18.7 ($CH_3$), 18.9 ($CH_3$), 127.9 (CH), 127.09 (CH), 128.5 (CH), 129.0 (CH), 133.3 (C), 133.8 (C), 135.8 (C), 135.9 (C),159.7 (C), 165.0 (C). IR (ATR, 32 scans, $cm^{-1}$): $\tilde{\nu}$ = 3231 (m), 3182 (w), 2881 (m), 2753 (w), 2741 (w), 1960 (w), 1873 (w), 1787 (w), 1653 (s), 1593 (m), 1519 (m), 1494 (m), 1471 (m), 1436 (m), 1383 (s), 1300 (w), 1284 (w), 1259 (m), 1226 (m), 1175 (w), 1152 (m), 1090 (w), 1045 (w), 1034 (w), 983 (w), 927 (w), 898 (w), 876 (m), 800 (w), 779 (vs), 729 (m), 709 (s), 643 (m), 565 (w), 521 (m), 482 (m). Raman (633 nm, 20 s, 20 scans, $cm^{-1}$): $\tilde{\nu}$ = 3235 (1), 3183 (1), 3074 (1), 3044 (2), 3015 (1), 2987 (1), 2951 (2), 2922 (5), 2884 (2), 2743 (1), 2582 (1), 2565 (1), 2046 (1), 1960 (1), 1655 (3), 1600 (2), 1592 (2), 1523 (1), 1475 (1), 1444 (1), 1384 (7), 1381 (6), 1301 (1), 1286 (1), 1259 (4), 1228 (1), 1174 (1), 1154 (1), 1091 (3), 1046 (1), 1025 (1), 995 (2), 981 (1), 929 (1), 891 (1), 876 (1), 799 (1), 778 (1), 737 (1), 711 (2),

645 (10), 565 (1), 523 (2), 514 (1), 492 (2), 484 (2), 383 (1), 326 (1), 298 (2), 282 (4), 238 (4), 202 (2). MS (GC-MS) m/z (%): 119 (13) [$C_7H_5NO$]$^+$, 134 (100) [$C_8H_8NO$]$^+$, 149 (36) [M]$^+$.

$^1$H NMR spectrum

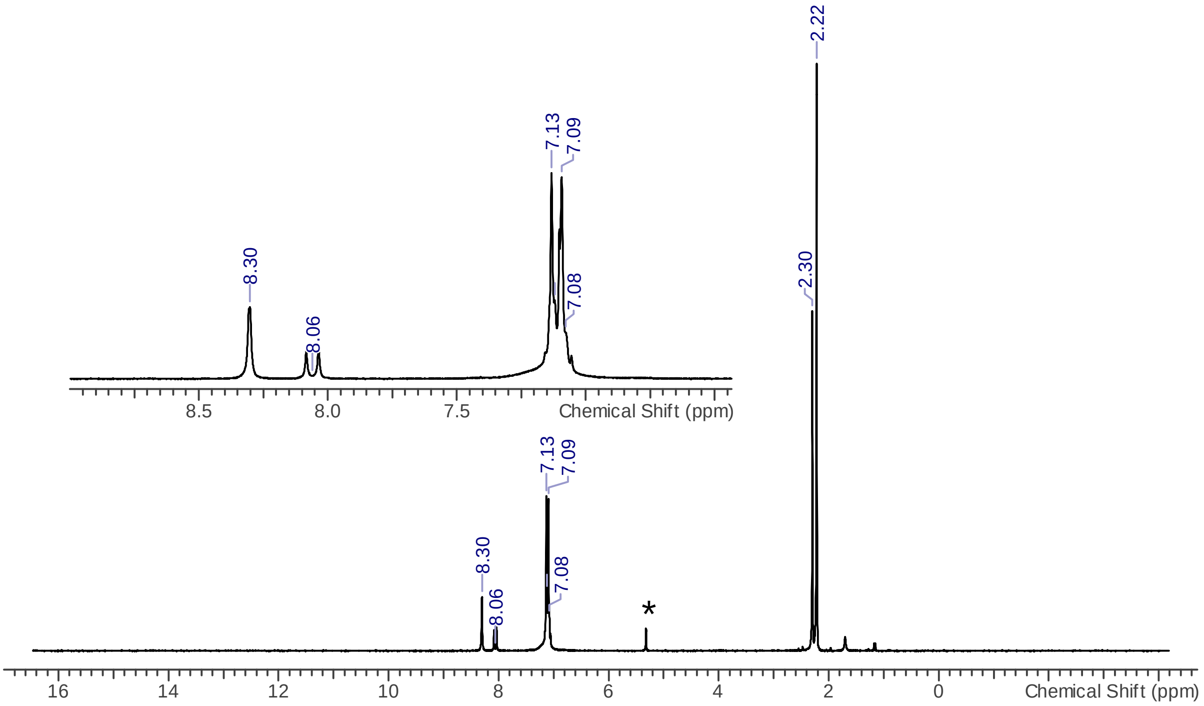


$^{13}$C{$^1$H} NMR spectrum

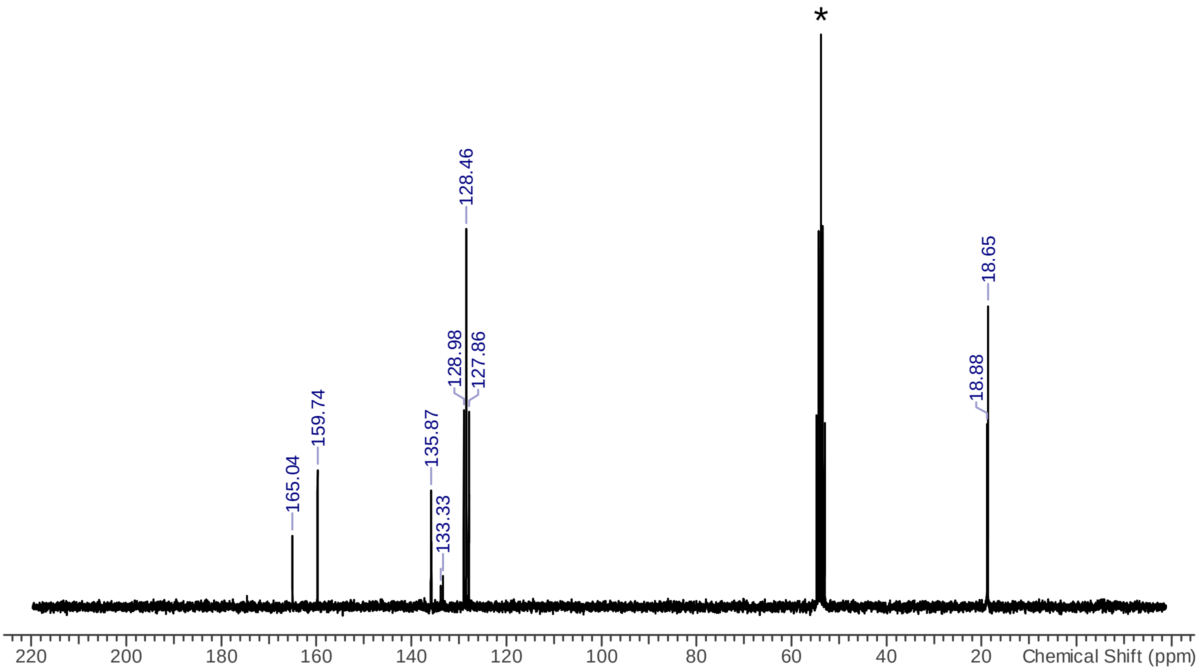

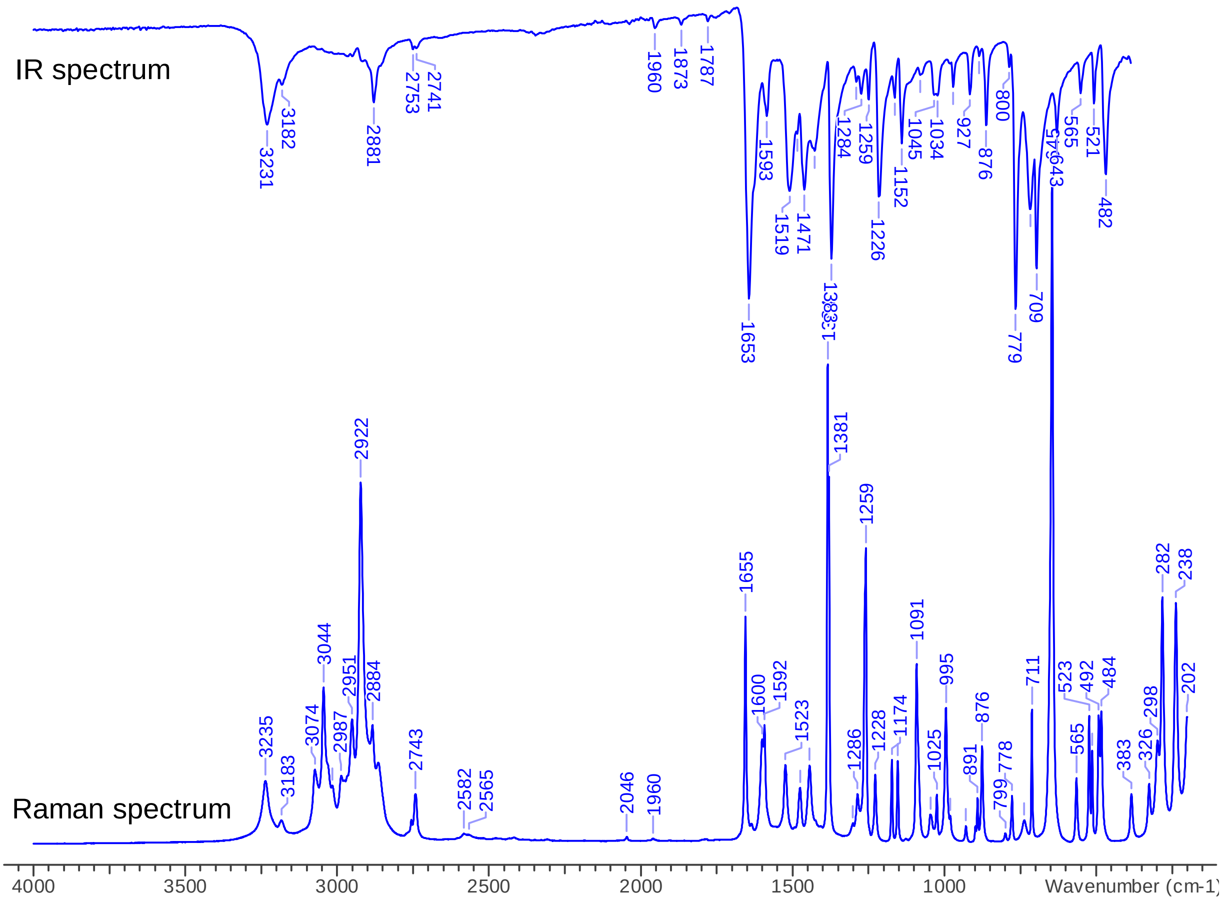


Figure S10. NMR, Raman and IR spectra of DmpN(H)COH in $CD_2Cl_2$ (solvent signals are indicated by asterisks).

## 2.1.2 DmpNC

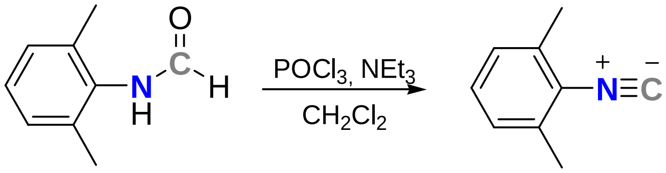


DmpNC was synthesized according to our previously published synthesis procedure.[S13] The synthesis was carried out under non-inert conditions.

N-(2,6-dimethylphenyl)formamide (5.423 g, 36.34 mmol) was dissolved in $CH_2Cl_2$ (50 mL). Two equivalents of phosphoryl trichloride (11.16 g, 72.75 mmol) were added at 0 °C followed by the dropwise addition of 10 equivalents of $NEt_3$ (30.55 g, 363.4 mmol). The reaction mixture was stirred for 16 h, whereupon it turned orange and became cloudy.

Afterwards, the orange suspension was diluted in ice water (100 mL). The aqueous phase was extracted three times with $CH_2Cl_2$ (40 mL). The combined organic layers were extracted two times with water (50 mL) and two times with a saturated $NaHCO_3$ solution (50 mL).

The solvent of the organic phase was removed in vacuo ($1\times10^{-3}$ mbar) and the crude product was purified by distillation of the melt (80 °C, $1\times10^{-3}$ mbar, oil bath). Afterwards, the pale yellow product was sublimed in vacuo at 60 °C ($1\times10^{-3}$ mbar, oil bath) yielding large colorless crystals of 2,6-dimethylphenyl isocyanide. Yield: 3.271 g (24.94 mmol, 69%).

$C_9H_9N$ (131.18 g/mol). Mp. 76 °C. CHN calcd. (found) in %: C 82.41 (82.46), H 6.92 (6.92), N 10.68 (10.66). $^1$H NMR ($CD_2Cl_2$, 300.1 MHz): $\delta$ = 2.41 (s, 6 H, o-$CH_3$), 7.00-7.20 (m, 3H, m,p-CH). $^{13}$C{$^1$H} NMR ($CD_2Cl_2$, 75.5 MHz): $\delta$ = 19.1 (o-$CH_3$), 128.1 (p-CH), 129.0 (m-CH), 135.3 (o-C), 168.7 (i-C), NC not observed. IR (ATR, 32 scans, cm$^{-1}$): $\tilde{\nu}$ = 3233 (w), 3184 (w), 2984 (w), 2947 (w), 2920 (w), 2881 (w), 2739 (w), 2120 (m), 2085 (w), 1949 (w), 1879 (w), 1811 (w), 1655 (m), 1591 (w), 1525 (w), 1490 (w), 1471 (m), 1440 (m), 1379 (m), 1302 (w), 1282 (w), 1228 (w), 1170 (m), 1084 (m), 1036 (m), 991 (w), 977 (w), 923 (w), 800 (w), 775 (vs), 721 (m), 637 (w), 548 (w). Raman (633 nm, 20 s, 20 scans, cm$^{-1}$): $\tilde{\nu}$ = 3071 (3), 3043 (3), 2985 (3), 2947 (4), 2919 (10), 2911 (9), 2885 (3), 2882 (3), 2873 (3), 2863 (4), 2740 (3), 2735 (3), 2119 (7), 1600 (1), 1590 (2), 1471 (1), 1464 (1), 1437 (1), 1423 (1), 1408 (1), 1383 (1), 1373 (1), 1264 (1), 1254 (2), 1171 (2), 1092 (1), 1078 (2), 990 (1), 796 (1), 779 (1), 727 (1), 719 (1), 636 (8), 565 (1), 542 (1), 518 (1), 505 (1), 491 (1), 458 (1), 361 (4), 284 (1), 240 (2). MS (GC-MS) m/z (%): 103 (30) $[C_7H_4N]^+$, 116 (62) $[C_8H_8N]^+$, 130 (100) $[C_9H_8N]^+$, 131 (68) $[M]^+$.

$^1H$ NMR spectrum

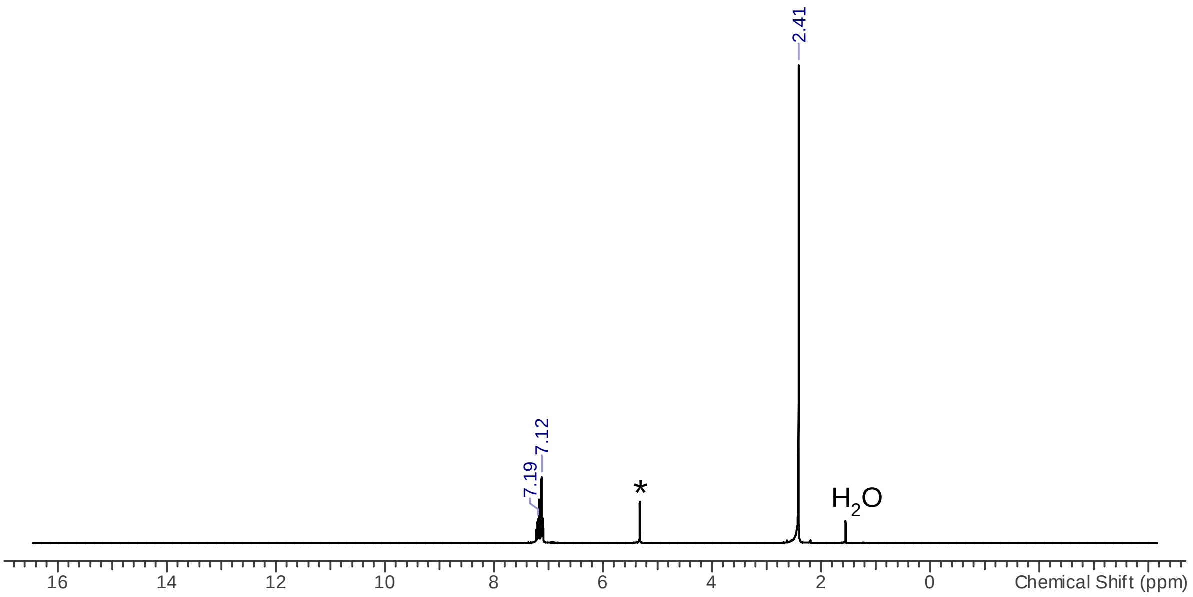


$^{13}C\{^1H\}$ NMR spectrum

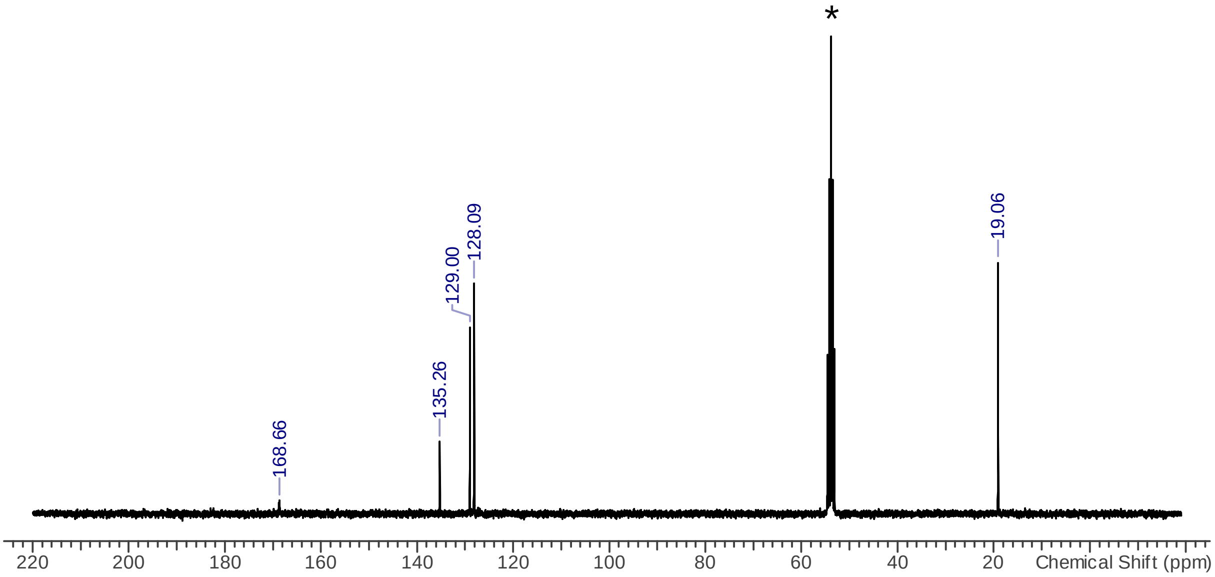

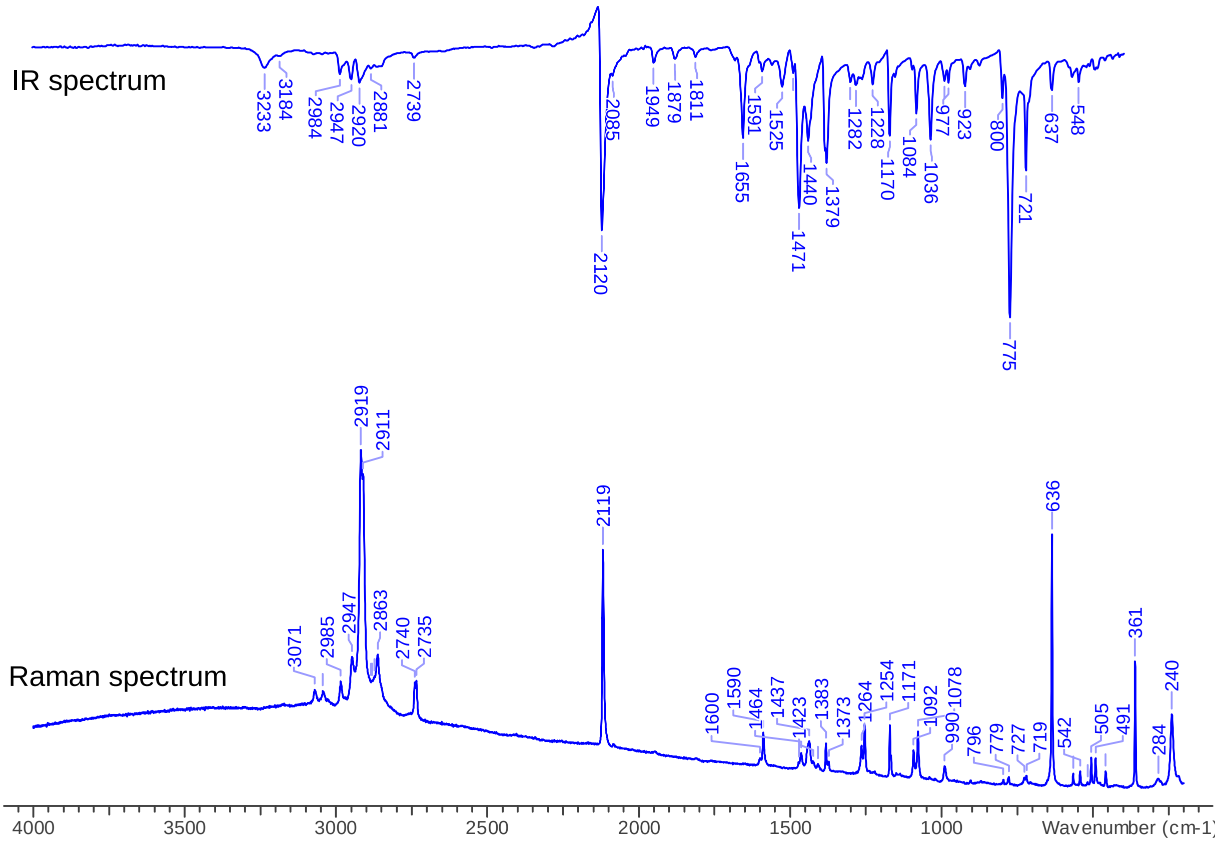


Figure S11. NMR, Raman and IR spectra of DmpNC in $CD_2Cl_2$ (solvent signals indicated by asterisks).

## 2.1.3 $AsCl_3$

$$As_2O_3 \quad + \quad 3\ SOCl_2 \longrightarrow AsCl_3 \quad + \quad 3\ SO_2$$

$AsCl_3$ was synthesized according to a modified literature procedure.[S14]

Finely powdered arsenic(III) oxide (61.001 g, 310.15 mmol) was filled into a 500 mL three-necked SCHLENK flask equipped with a condenser and dropping funnel. Under stirring, thionyl chloride (198.918 g, 115 mL, 1.672 mol) was added dropwise (1 drop/sec) at 0 °C (ice bath). After approx. one third of the thionyl chloride had been added (after ca. 30 min), the reaction mixture was allowed to warm to ambient temperature. Due to the reaction heat, the mixture started to boil slowly, and the solids dissolved completely, whereupon the mixture was cooled again using an ice bath. Once all the thionyl chloride had been added, the yellow solution was stirred for further 100 hours at ambient temperature. The crude product was distilled at atmospheric

pressure: At an oil bath temperature of 110 °C, unreacted thionyl chloride was recovered ($T_{vap}$ 77 °C). Heating the oil bath to 155 °C yielded the first fraction (8 mL) of pale-yellow arsenic trichloride, which contained some last traces of thionyl chloride. Upon heating the oil bath to 160 °C, clear, colourless $AsCl_3$ could be collected. Yield: 75.238 g (415.04 mmol, 67%).

Raman (633 nm, 20 s, 20 scans, $cm^{-1}$): $\tilde{\nu}$ = 406 (10), 372 (6), 192 (7), 155(9).

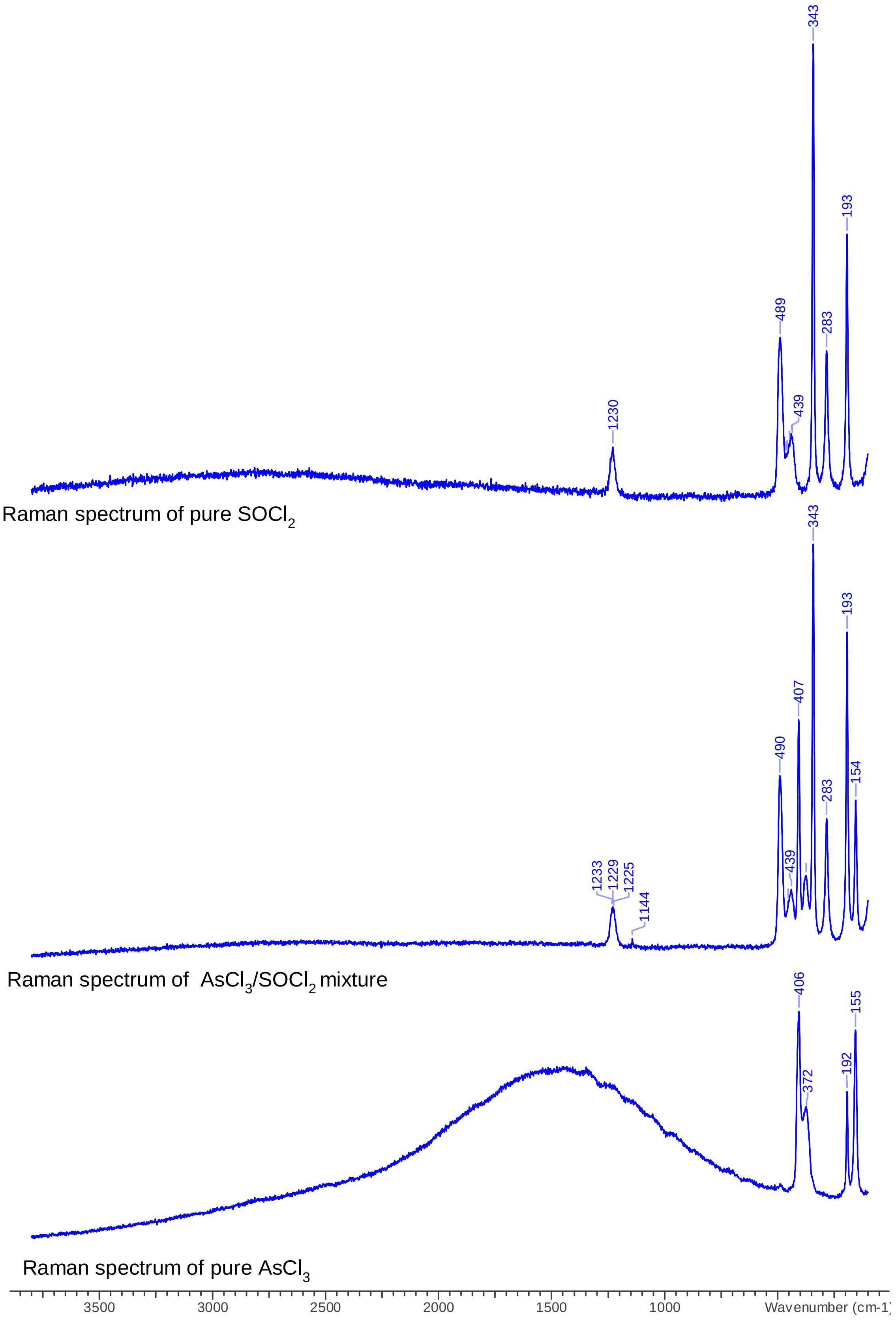


Figure S12. Raman spectra of $SOCl_2$, $SOCl_2/AsCl_3$ mixture and $AsCl_3$.

### 2.1.4 TerNPN(H)Ter

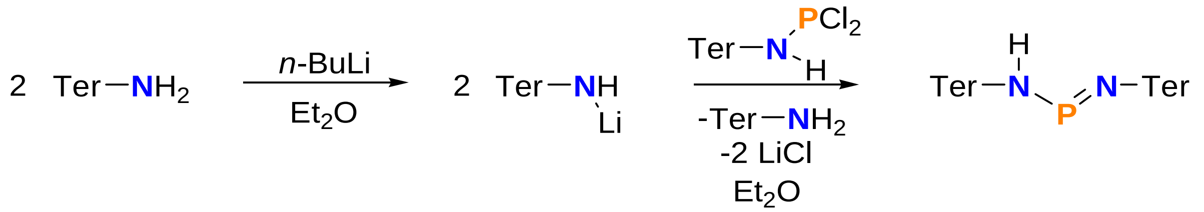


TerNPN(H)Ter was synthesized according to a modified literature procedure.[S15]

$TerNH_2$ (15.037 g, 45.637 mmol) was dissolved in $Et_2O$ (200 mL). The solution was cooled to 0 °C (ice bath) and n-BuLi (2.5 M, 19 mL) was added dropwise over a period of 10 minutes. The reaction mixture was slowly (15 minutes) warmed to ambient temperature and stirred for 2 h. The reaction solution was cooled to 0 °C (ice bath), whereupon a solution of $TerN(H)PCl_2$ (9.558 g, 22.21 mmol) in $Et_2O$ (100 mL) was added over a period of 20 minutes. The mixture was stirred overnight at ambient temperature. Afterwards, all volatiles were removed in vacuo ($1\times10^{-3}$ mbar) and the remaining solids were dried in vacuo ($1\times10^{-3}$ mbar) at 50 °C (water bath) for 30 min. The solids were suspended in benzene (400 mL), and the insoluble material was allowed to settle overnight. The mixture was then filtered over a celite-packed frit. To avoid clogging, most of the clear supernatant was decanted through the frit, while the insoluble material was filled onto the frit only at the end of the filtration. The solvent of the filtrate was removed in vacuo ($1\times10^{-3}$ mbar) and the residue was dried in vacuo ($1\times10^{-3}$ mbar) at 50 °C (water bath) for 30 min. The solid raw product was finely powdered and filled in a sublimation apparatus to remove excess $TerNH_2$ (sublimation at 70 °C, $1\times10^{-3}$ mbar, oil bath). To speed up the sublimation, it proved advantageous to use a large sublimation apparatus, so as to increase the surface-to-volume ratio of the powder bed. The sublimation was carried out until $TerNH_2$ could no longer be detected in the $^1H$ NMR spectrum (ca. 140 h). Yield: 13.713 g (19.997 mmol, 90%).

$C_{48}H_{51}N_2P$ (686.92 g/mol). Mp. 233 °C (decompn.). CHN % calcd. (found): C 83.93 (83.51), H 7.48 (7.09), N 4.08 (4.34). $^1H$ NMR ($C_6D_6$, 300.1 MHz): $\delta$ = 1.81 (s, 12 H, o-$CH_3$), 1.95 (s, 12 H, o-$CH_3$), 2.24 (s, 6 H, m-$CH_3$), 2.26 (s, 6 H, m-$CH_3$), 6.70 (d, $^1J(^1H,^{14}N)$=

9.2 Hz,1 H, NH), 6.81 (m, 4 H, m-CH), 6.82 (m, 4 H, m-CH), 6.89 (m, 1 H, p-CH), 6.91 (m, 1 H, p-CH), 6.96 (s, 2 H, m-CH), 6.99 (s, 2 H, m-CH). $^{13}C\{^{1}H\}$ NMR ($C_6D_6$, 75.5 MHz): $\delta$ = 20.4 (s, $CH_3$), 20.4 (s, $CH_3$), 21.0 (s, $CH_3$), 21.0 (s, $CH_3$), 21.7 (s,), 21.7 (s, $CH_3$), 122.5 (s, arom. CH), 123.5 (s, arom. CH), 128.5 (s, arom. CH), 129.1 (s, arom. CH), 129.5 (s, arom. CH), 129.8 (s, arom. CH), 131.2 (d, $J(^{13}C,^{31}P)$ = 2 Hz, arom. C), 131.7 (d, $J(^{13}C,^{31}P)$ = 8 Hz, arom. C), 136.0 (s, arom. CH), 136.8 (s, arom. CH), 136.9 (s, arom. CH), 136.9 (s, arom. CH), 137.9 (s, arom. CH), 138.5 (s, arom. CH), 144.3 (s, arom. C), 144.4 (s, arom. C). $^{14}N$ NMR: no signals observed. $^{31}P\{^{1}H\}$ NMR ($C_6D_6$, 101.3 MHz): $\delta$ = 276.6 (s, 1P). IR (ATR, 32 scans, $cm^{-1}$): $\tilde{\nu}$ = 3357 (w), 3311 (w), 3289 (w), 2996 (w), 2957 (w), 2943 (w), 2914 (m), 2854 (w), 2798 (w), 2730 (w), 2403 (w), 1611 (w), 1572 (w), 1488 (w), 1434 (m), 1422 (m), 1372 (m), 1304 (w), 1261 (m), 1234 (m), 1216 (m), 1183 (m), 1158 (w), 1127 (w), 1098 (m), 1074 (m), 1030 (m), 1008 (m), 968 (m), 948 (m), 917 (s), 849 (vs), 822 (m), 795 (m), 775 (m), 752 (s), 738 (m), 647 (m), 628 (m), 618 (m), 598 (m), 560 (m), 550 (m), 528 (m), 492 (m), 482 (m), 462 (m), 427 (m). Raman (633 nm, 15 s, 15 scans, $cm^{-1}$): $\tilde{\nu}$ = 3293 (1), 3055 (2), 3011 (2), 2914 (3), 2855 (2), 2729 (1), 1613 (2), 1588 (5), 1481 (2), 1442 (9), 1412 (5), 1379 (3), 1363 (2), 1344 (2), 1307 (10), 1268 (2), 1226 (3), 1183 (2), 1166 (2), 1159 (2), 1086 (5), 1078 (3), 1034 (3), 1008 (3), 948 (2), 922 (2), 822 (2), 778 (3), 757 (2), 734 (2), 656 (3), 620 (2), 596 (2), 579 (4), 557 (3), 543 (2), 532 (2), 520 (3), 497 (2), 483 (2), 412 (2), 392 (2), 380 (2), 365 (2), 335 (2), 266 (3), 257 (2), 230 (2), 166 (2), 139 (2), 98 (10). MS (CI, pos., isobutane) m/z (%): 330 (14) $[TerNH_3]^+$, 671 (15), 687 (100) $[M]^+$, 705 (25), 743 $[M+C_4H_9]^{+\cdot}$

$^{1}$H NMR spectrum

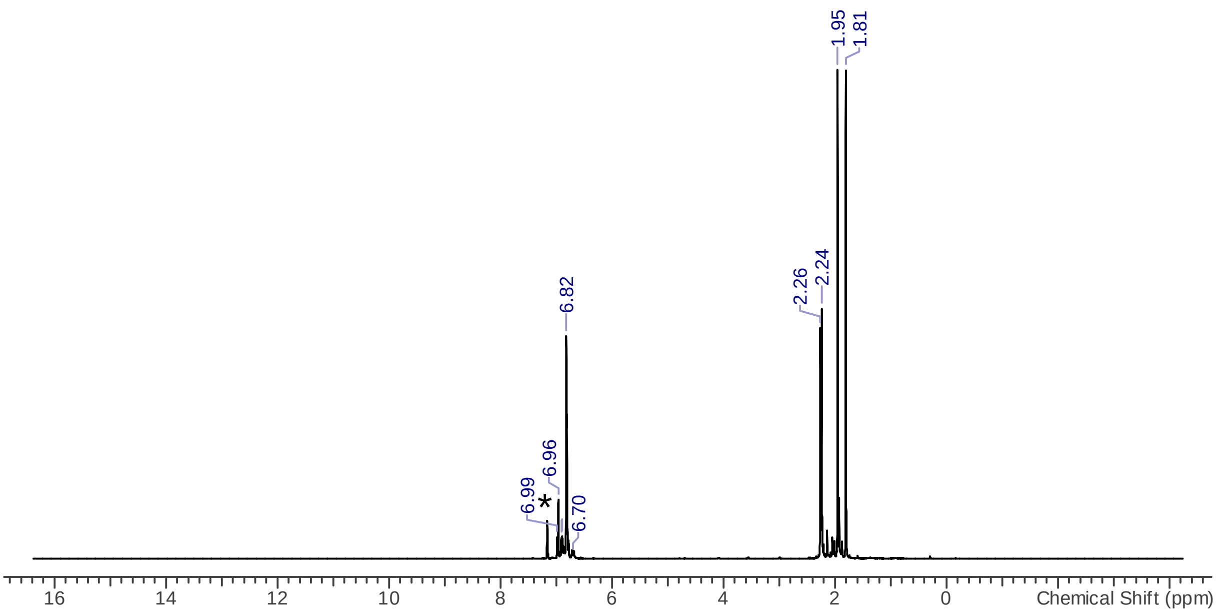


$^{13}$C{$^{1}$H} NMR spectrum

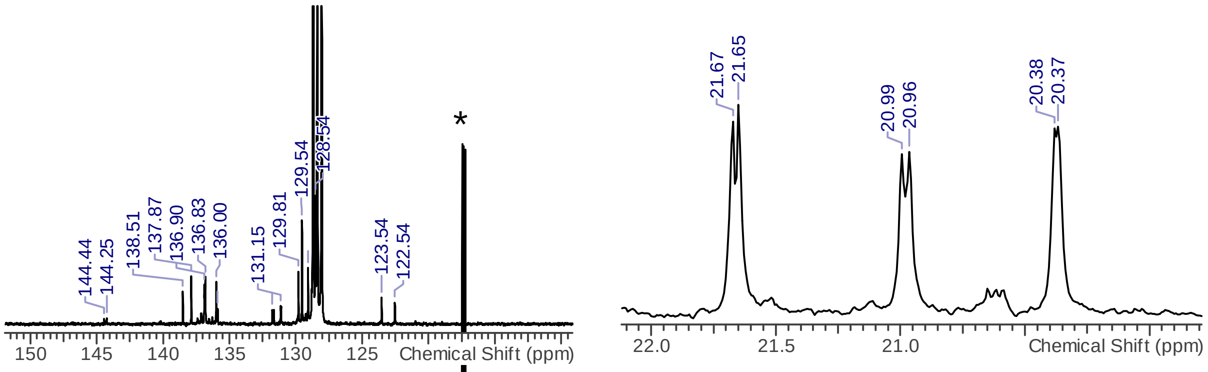


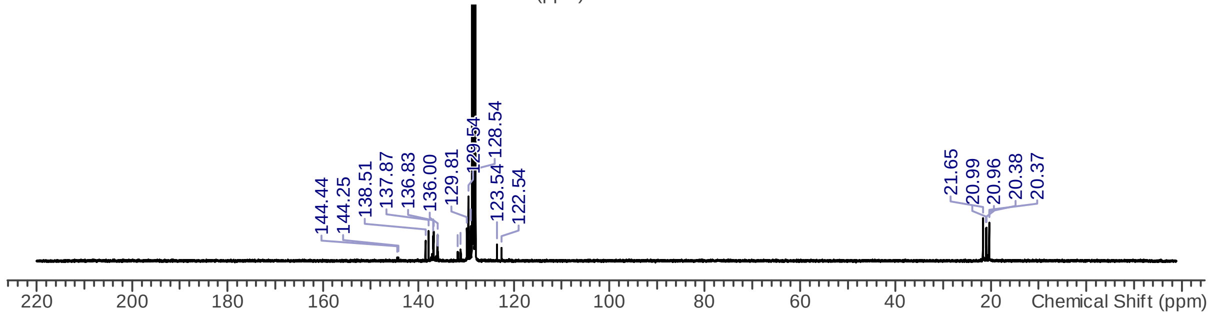

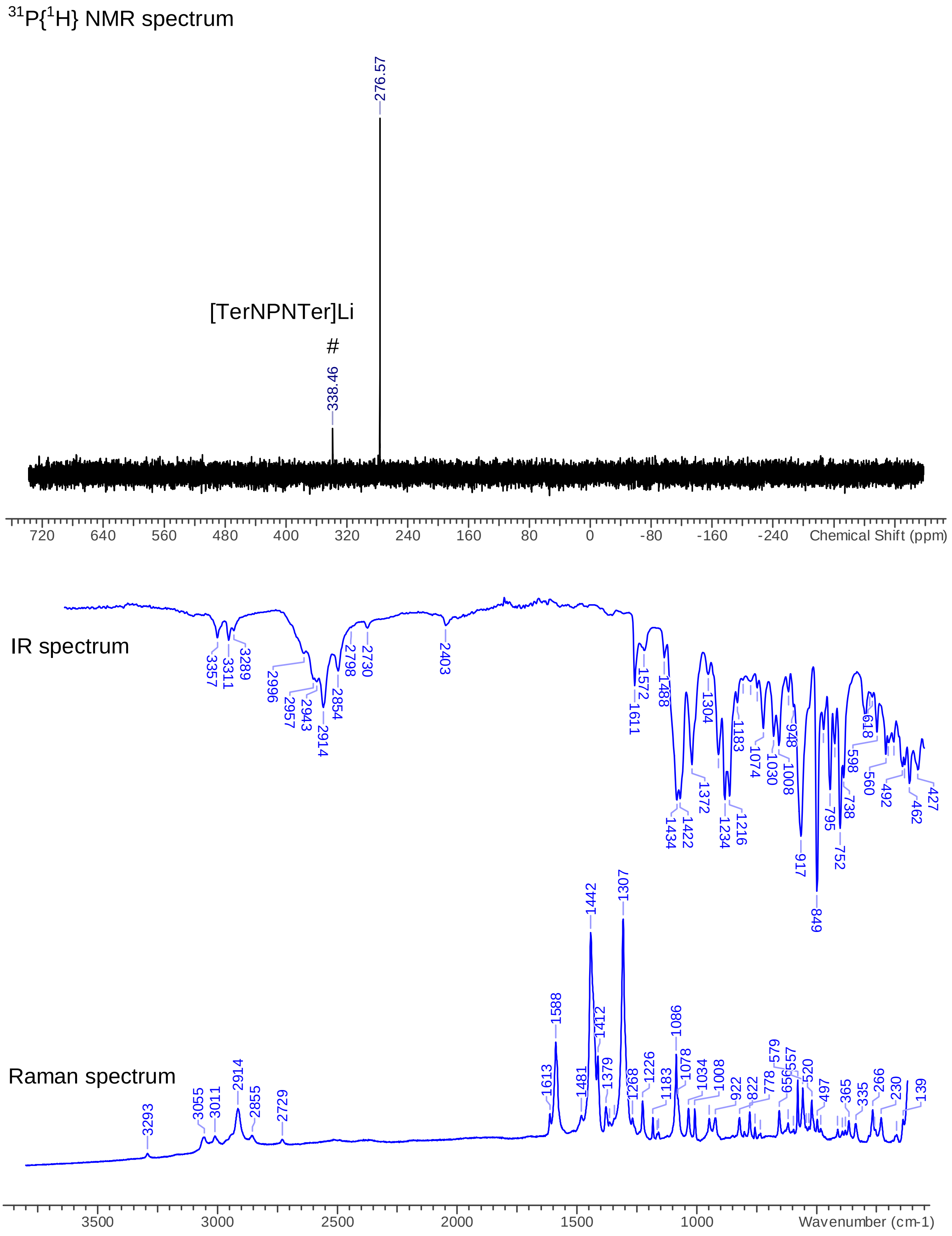


Figure S13. NMR, IR and Raman spectra of TerNPN(H)Ter (solvent signals are marked by an asterisk).

### 2.1.5 [TerNPNTer]K

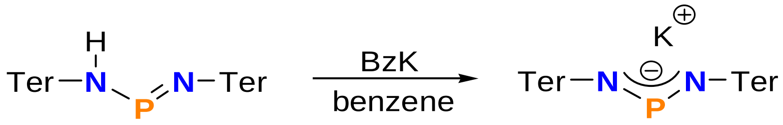


[TerNPNTer]K was synthesized according to a modified literature procedure.[S15]

BzK (2.583 g, 19.84 mmol) was added in portions to a vigorously stirred solution of TerNPN(H)Ter (13.608 g, 19.836 mmol) in benzene at ambient temperature over a period of 30 minutes. The progress of the reaction must be monitored by $^{31}$P NMR spectroscopy to check the completeness of the reaction. Signals from intermediate species (101 ppm, 128 ppm) were detected. Afterwards, more BzK (6.51 mg in 3 portions, 0.05 mmol) was added and the reaction progress was monitored by $^{31}$P NMR spectroscopy. The reaction was completed when all signals of the intermediate species had disappeared and only the signal of the product (326 ppm) could be detected. Once the reaction was completed, the solvent was removed in vacuo ($1\times10^{-3}$ mbar) and the residue was dried in vacuo ($1\times10^{-3}$ mbar) at 50 °C (water bath) for 60 min. The residue was then suspended in benzene (250 mL), and the insoluble material was allowed to settle overnight. The mixture was then filtered over a celite-packed frit and the filtrate was concentrated to incipient crystallization. After crystallizing overnight at ambient temperature, a small amount of pale yellow crystals of the product was obtained. The oily red supernatant was removed by syringe and subsequently dried in vacuo ($1\times10^{-3}$ mbar) for 30 minutes. Since the product could only be crystallized in low yields, the dried supernatant was also used for the synthesis of [ClP(μ-NTer)$_2$AsCl] (2.1.6). The isolated crystals were dried in vacuo ($1\times10^{-3}$ mbar). Yield: 2.879 g (3.976 mmol, 20%).

$C_{48}H_{50}N_2PK$ (724.08 g/mol). Mp. 268 °C (decompn.). CHN % calcd. (found): C 79.52 (79.71), H 6.95 (7.09), N 3.86 (3.83). $^{1}$H NMR ($C_6D_6$, 250.1 MHz): $\delta$ = 2.02 (s, 12 H, o-CH$_3$), 2.25 (s, 6 H, p-CH$_3$), 6.71 (m, 4 H, m-CH), 6.88 (m, 2 H, m-CH), 6.89 (m, 1 H, p-CH). $^{13}$C{$^{1}$H} NMR ($C_6D_6$, 62.9 MHz): $\delta$ = 21.3 (s, CH$_3$), 21.3 (s, CH$_3$), 21.0 (s, CH$_3$), 21.6 (s, CH$_3$), 119.6 (s, arom. CH), 128.4 (s, arom. CH), 129.4 (s, arom. CH), 133.1 (d, J($^{13}$C,$^{31}$P) = 8 Hz, arom. C), 135.3 (s, arom. CH), 137.2 (d, J($^{13}$C,$^{31}$P) = 1 Hz, arom. C), 141.8 (s, arom.

CH), 149.4 (d, arom. C, $^{2}J(^{13}C,^{31}P)$ = 18 Hz). $^{14}N$ NMR ($C_6D_6$, 18.1 MHz): $\delta$ = −188 (s, 2N). $^{31}P\{^{1}H\}$ NMR ($C_6D_6$, 101.3 MHz): $\delta$ = 326.0 (s, 1P). IR (ATR, 32 scans, $cm^{-1}$): $\tilde{\nu}$ = 3324 (w), 2951 (w), 2916 (w), 2854 (w), 2732 (w), 1611 (w), 1574 (w), 1482 (w), 1453 (m), 1436 (m), 1405 (m), 1377 (m), 1269 (w), 1224 (s), 1187 (w), 1164 (w), 1098 (w), 1082 (m), 1032 (w), 1014 (w), 1004 (w), 954 (w), 896 (m), 845 (vs), 793 (m), 762 (m), 750 (s), 696 (w), 682 (m), 645 (w), 589 (w), 571 (w), 558 (w), 548 (w), 540 (m), 511 (w), 501 (w), 476 (w), 445 (m), 429 (m), 412 (m). Raman (633 nm, 10 s, 10 scans, $cm^{-1}$): $\tilde{\nu}$ = 3011 (1), 2917 (1), 2852 (1), 2724 (1), 2499 (1), 1613 (3), 1604 (3), 1580 (4), 1559 (3), 1481 (3), 1416 (10), 1384 (3), 1375 (3), 1371 (3), 1349 (3), 1302 (10), 1282 (4), 1271 (3), 1265 (3), 1258 (3), 1247 (3), 1233 (3), 1181 (3), 1164 (3), 1155 (3), 1121 (3), 1109 (3), 1095 (3), 1083 (5), 1078 (4), 1006 (4), 992 (3), 979 (6), 956 (3), 948 (3), 944 (3), 860 (2), 845 (2), 841 (2), 810 (3), 794 (4), 775 (4), 765 (3), 754 (5), 735 (3), 654 (3), 645 (3), 619 (2), 600 (3), 580 (3), 556 (3), 546 (4), 523 (3), 517 (2), 506 (3), 499 (2), 489 (3), 480 (2), 415 (2), 376 (3), 339 (2), 326 (2), 278 (2), 267 (2), 236 (2), 218 (3), 181 (2), 144 (3), 100 (7), 82 (5). MS (CI, pos., isobutane) m/z (%): 330 (7) $[TerNH_3]^+$, 687 (10) $[TerNPN(H)Ter]^+$, 724 (100) $[M]^+$.

$^{1}H$ NMR spectrum

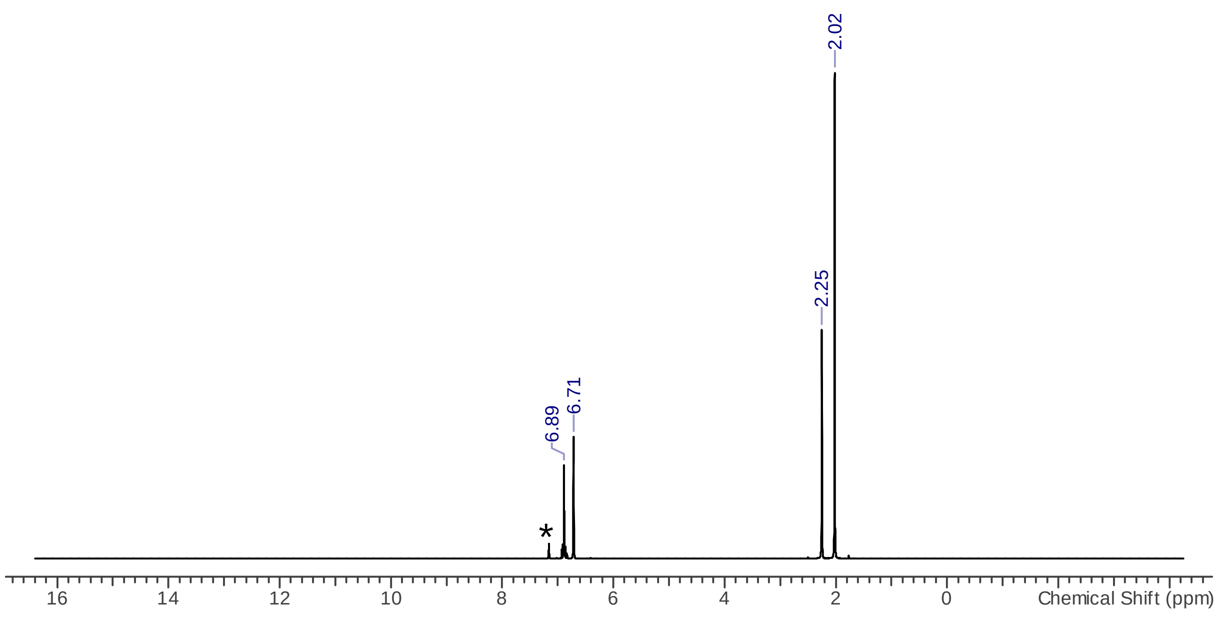

$^{13}C\{^1H\}$ NMR spectrum

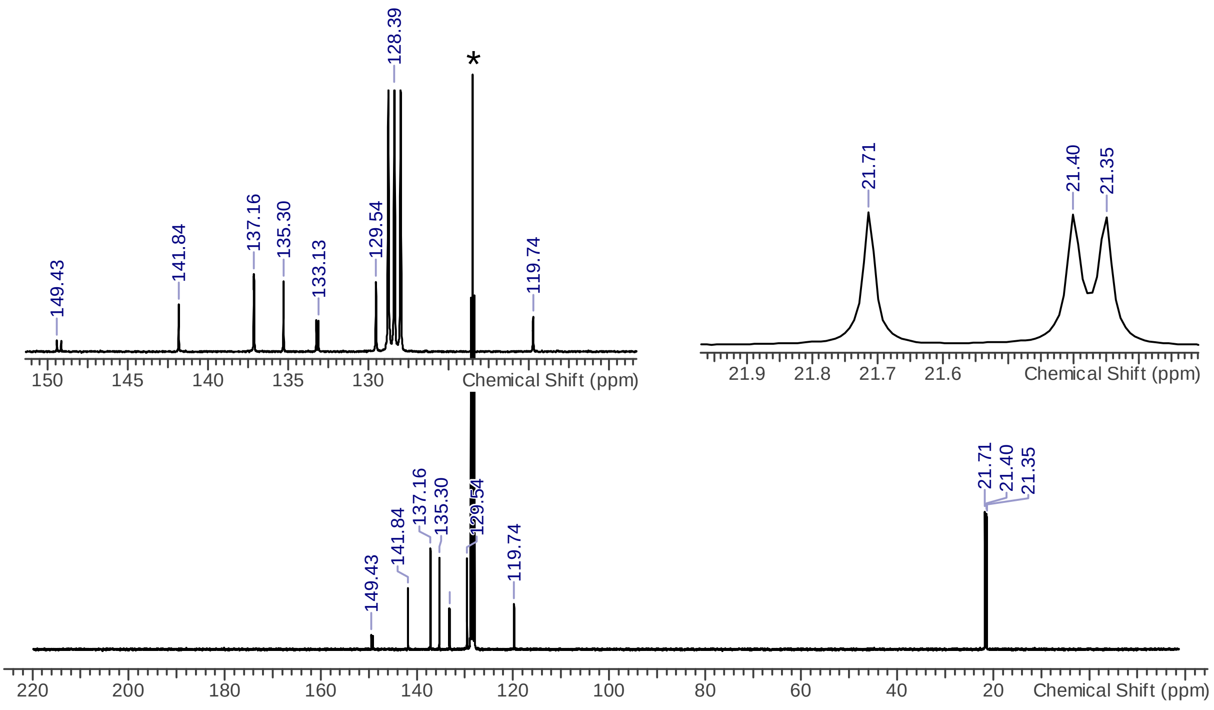


$^{31}P\{^1H\}$ NMR spectrum

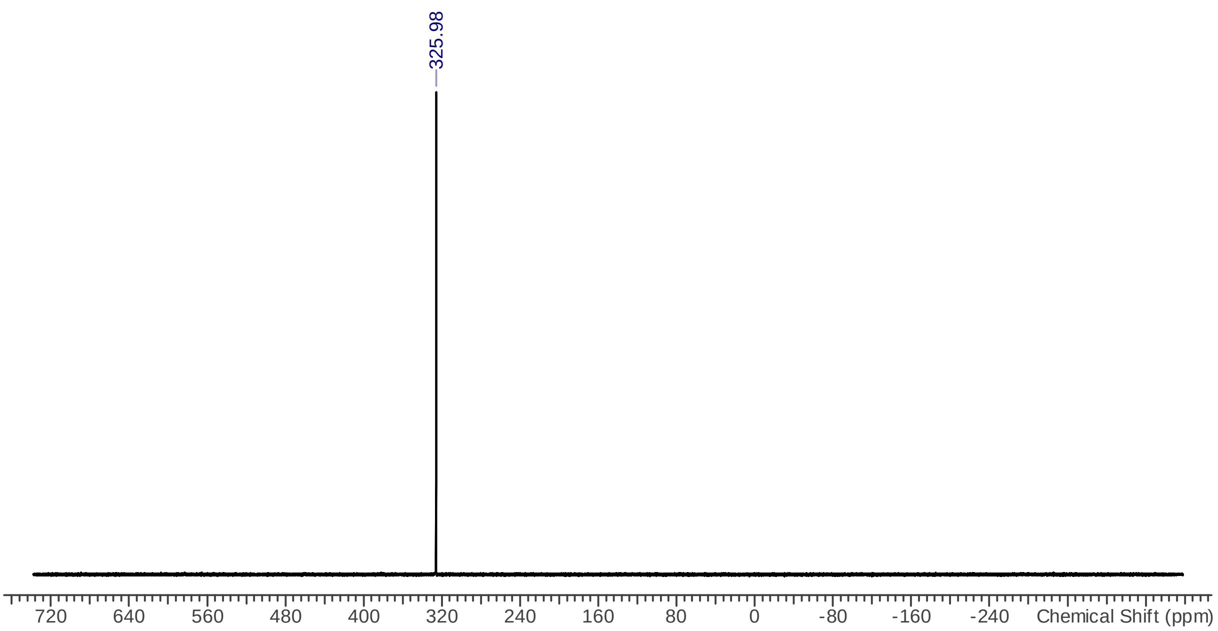

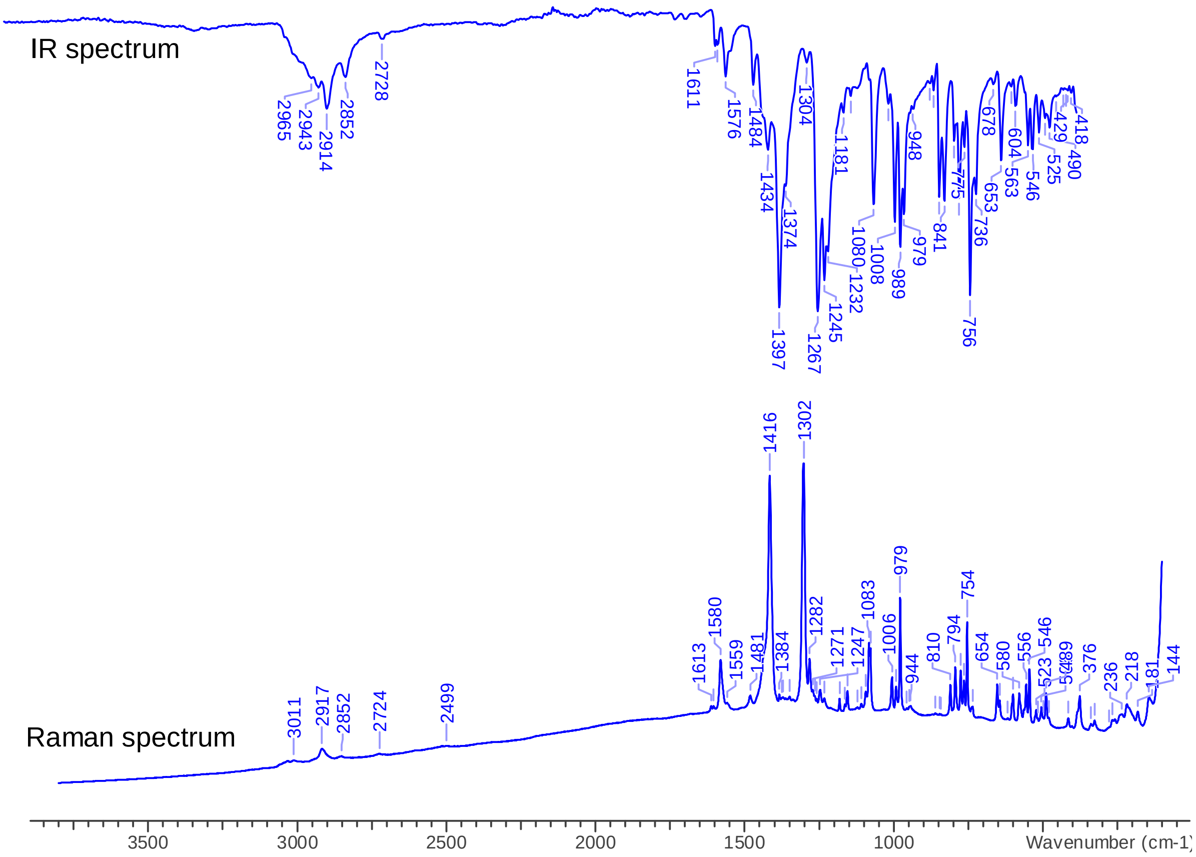


Figure S14. NMR, IR and Raman spectra of [TerNPNTer]K (solvent signals are marked by an asterisk).

## 2.1.6 [ClP(µ-NTer)$_2$AsCl]

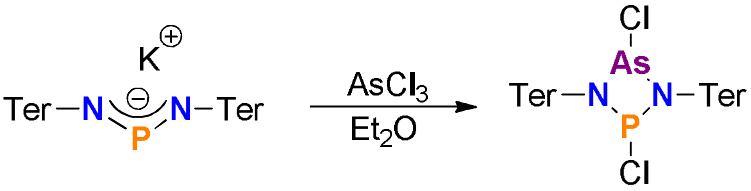


[ClP(µ-NTer)$_2$AsCl] was synthesized according to a modified literature procedure.[S15]

The red oily crude product of K[TerNPNTer] was used for the synthesis of [ClP(µ-NTer)$_2$AsCl].

A stirred solution of K[TerNPNTer] (14.342 g, 19.836 mmol) in $Et_2O$ (250 mL) was cooled to −90 °C, and $AsCl_3$ (9.532 g, 52.58 mmol) was added dropwise over a period of 15 minutes. The reaction mixture turned greenish, whereupon it was warmed to ambient temperature and stirred for 16 h. Afterwards, all volatiles were removed in vacuo

($1\times10^{-3}$ mbar) and the solid residue was dried in vacuo ($1\times10^{-3}$ mbar) for 60 minutes at 50 °C (water bath). The residue was then suspended in benzene (200 mL), and the insoluble material was allowed to settle overnight. The mixture was then filtered over a celite-packed frit. Afterwards, all volatiles were removed in vacuo ($1\times10^{-3}$ mbar) and the solid residue was dried in vacuo ($1\times10^{-3}$ mbar) for 30 minutes at 50 °C (water bath). The crude product was washed with 20 mL of n-hexane and the colourless product was dried in vacuo ($1\times10^{-3}$ mbar) for 30 minutes at 50 °C (water bath). Yield: 12.312 g (14.839 mmol, 75%).

$C_{48}H_{50}N_2PAsCl_2$ (830.74 g/mol). Mp. 210 °C (decompn.). CHN % calcd. (found): C 69.32 (68.89), H 6.06 (5.64), N 3.37 (3.12). $^{1}H$ NMR ($C_6D_6$, 500.1 MHz) Broad signals due to hindered rotation of the Ter subsituent: $\delta$ = 2.06 (broad, 6 H, $CH_3$), 2.19 (broad, 6 H, $CH_3$), 2.26 (broad, 6 H, $CH_3$), 2.35 (broad, 6 H, $CH_3$), 6.61 (broad, 4 H, m-CH), 6.73 (broad, 4 H, m-CH), 6.79 (broad, 2 H, m-CH), 6.85 (broad, 1 H, p-CH). $^{13}C\{^{1}H\}$ NMR ($C_6D_6$, 125.7 MHz): $\delta$ = 21.4 (s, $CH_3$), 22.1 (s, $CH_3$), 22.3 (s, $CH_3$), 123.9 (s, arom. CH), 128.7 (s, arom. CH), 128.9 (s, arom. CH), 129.8 (broad, arom. CH), 129.8 (broad, arom. CH), 130.9 (broad, arom. CH), 132.4 (broad, arom. CH), 132.8 (broad, arom. CH), 133.5 (broad, arom. CH), 135.0 (broad, arom. CH), 136.3 (broad, arom. CH), 137.5 (broad, arom. CH), 138.1 (broad, arom. CH), 138.9 (broad, arom. CH). $^{14}N$ NMR No signals observed. $^{31}P\{^{1}H\}$ NMR ($C_6D_6$, 202.8 MHz): $\delta$ = 268.4 (s, 1P). IR (ATR, 32 scans, $cm^{-1}$): $\tilde{\nu}$ = 3324 (w), 2951 (w), 2916 (w), 2854 (w), 2732 (w), 1611 (w), 1574 (w), 1482 (w), 1453 (m), 1436 (m), 1405 (m), 1377 (m), 1269 (w), 1224 (s), 1187 (w), 1164 (w), 1098 (w), 1082 (m), 1032 (w), 1014 (w), 1004 (w), 954 (w), 896 (m), 845 (vs), 793 (m), 762 (m), 750 (s), 696 (w), 682 (m), 645 (w), 589 (w), 571 (w), 558 (w), 548 (w), 540 (m), 511 (w), 501 (w), 476 (w), 445 (m), 429 (m), 412 (m). Raman (633 nm, 10 s, 10 scans, $cm^{-1}$): $\tilde{\nu}$ = 3040 (3), 2917 (4), 2853 (3), 1611 (8), 1576 (8), 1481 (7), 1441 (7), 1417 (8), 1380 (7), 1376 (7), 1302 (9), 1277 (8), 1188 (7), 1162 (7), 1101 (7), 1088 (7), 1005 (7), 991 (6), 956 (7), 943 (7), 741 (6), 736 (7), 694 (6), 646 (6), 572 (8), 558 (7), 538 (6), 522 (6), 512 (6), 499 (6), 483 (6), 466 (6), 443 (8), 430 (6), 412 (6), 378 (6), 333 (7), 328 (7), 274 (6), 258 (5), 239 (5), 213 (5),

202 (5), 166 (6), 159 (6), 142 (6), 117 (6), 103 (7), 90 (8), 78 (10). MS (CI, pos., isobutane) m/z (%): 795 (100) [HP(μ-TerN)$_2$AsCl]$^+$, 830 (26) [M]$^+$.

$^1$H NMR spectrum

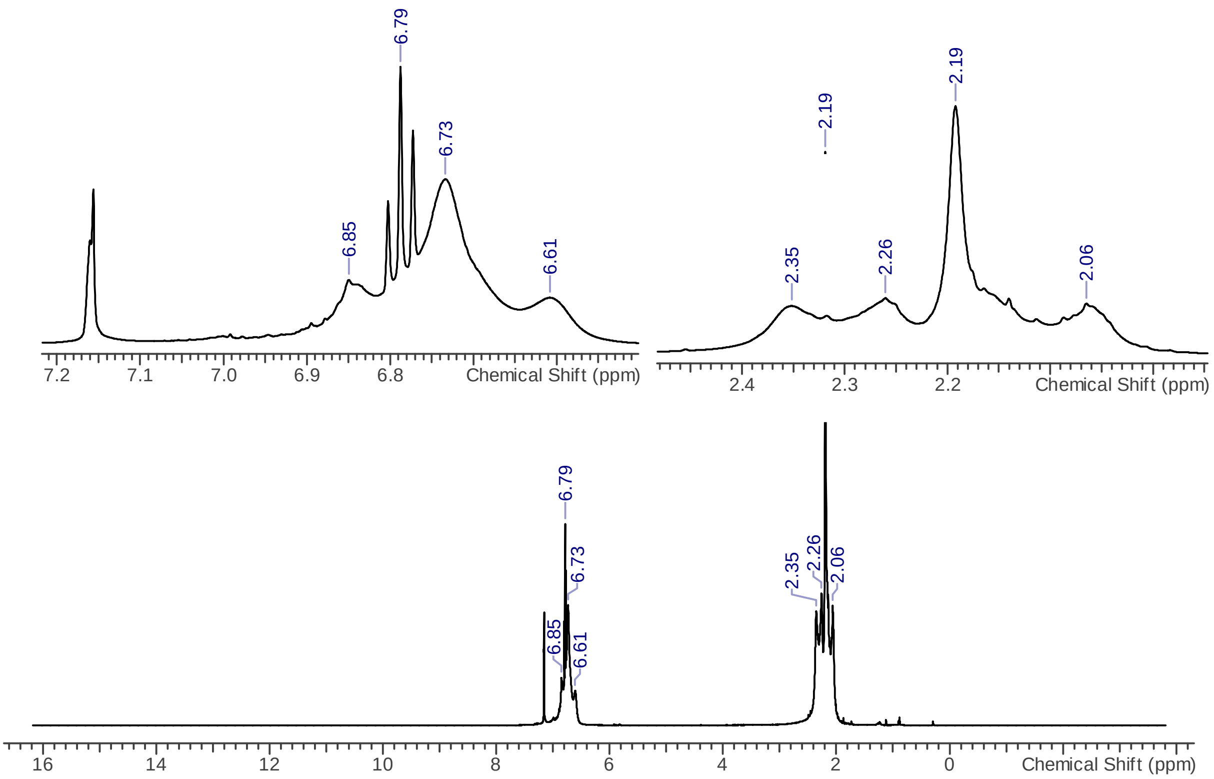

$^{13}C\{^1H\}$ NMR spectrum

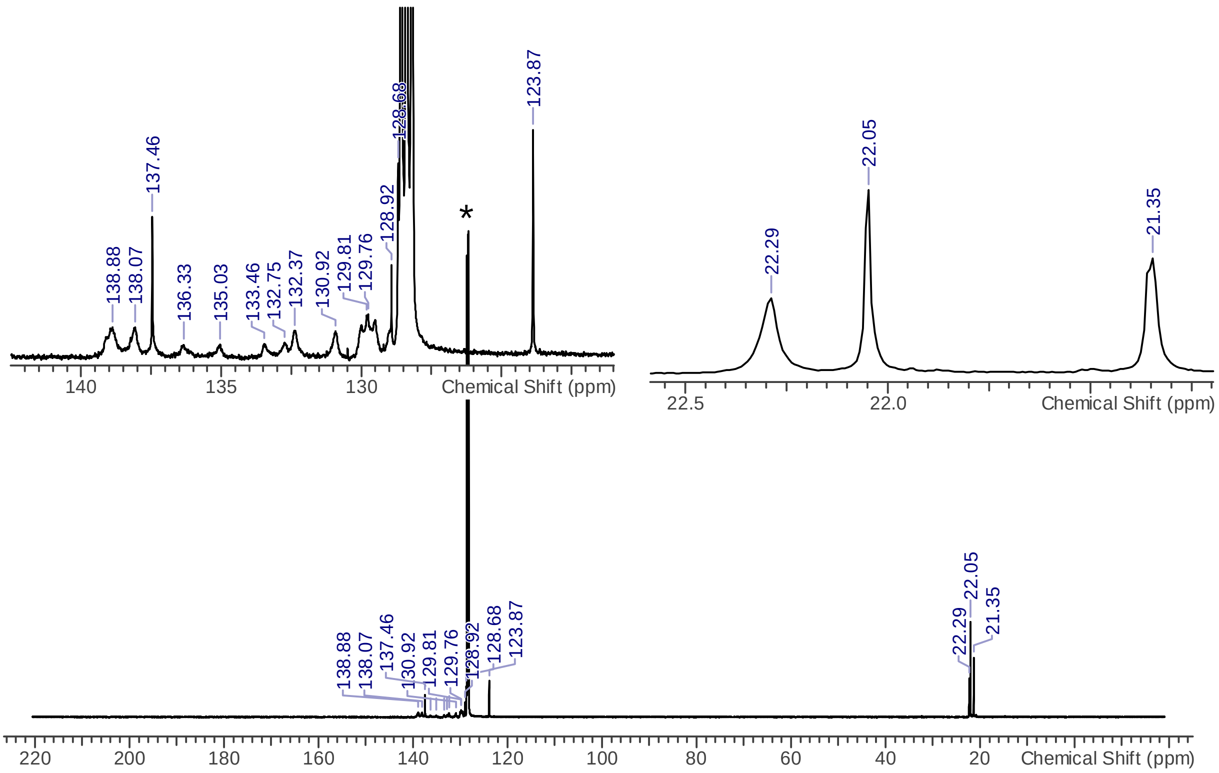


$^{31}P\{^1H\}$ NMR spectrum

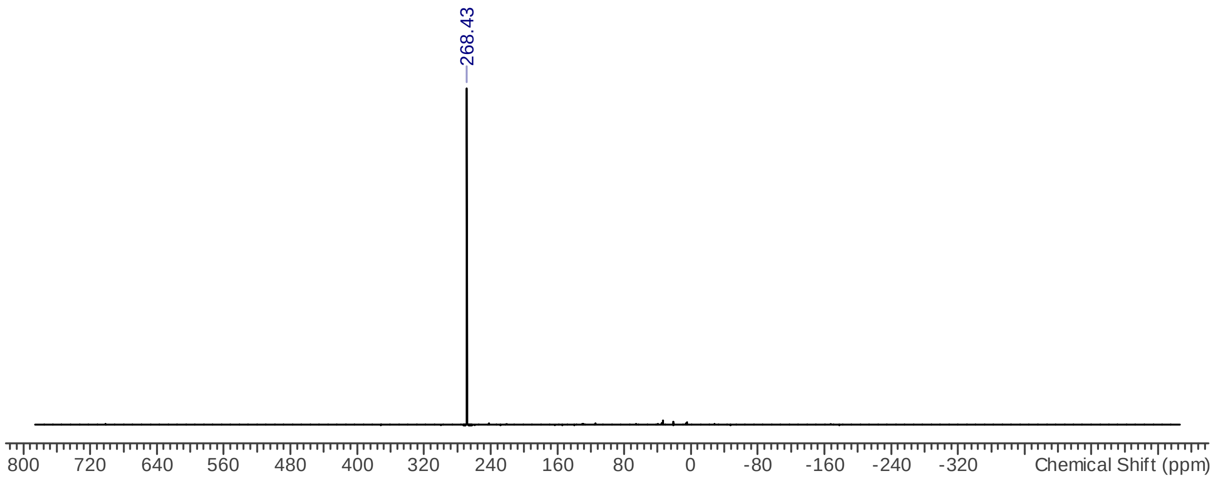

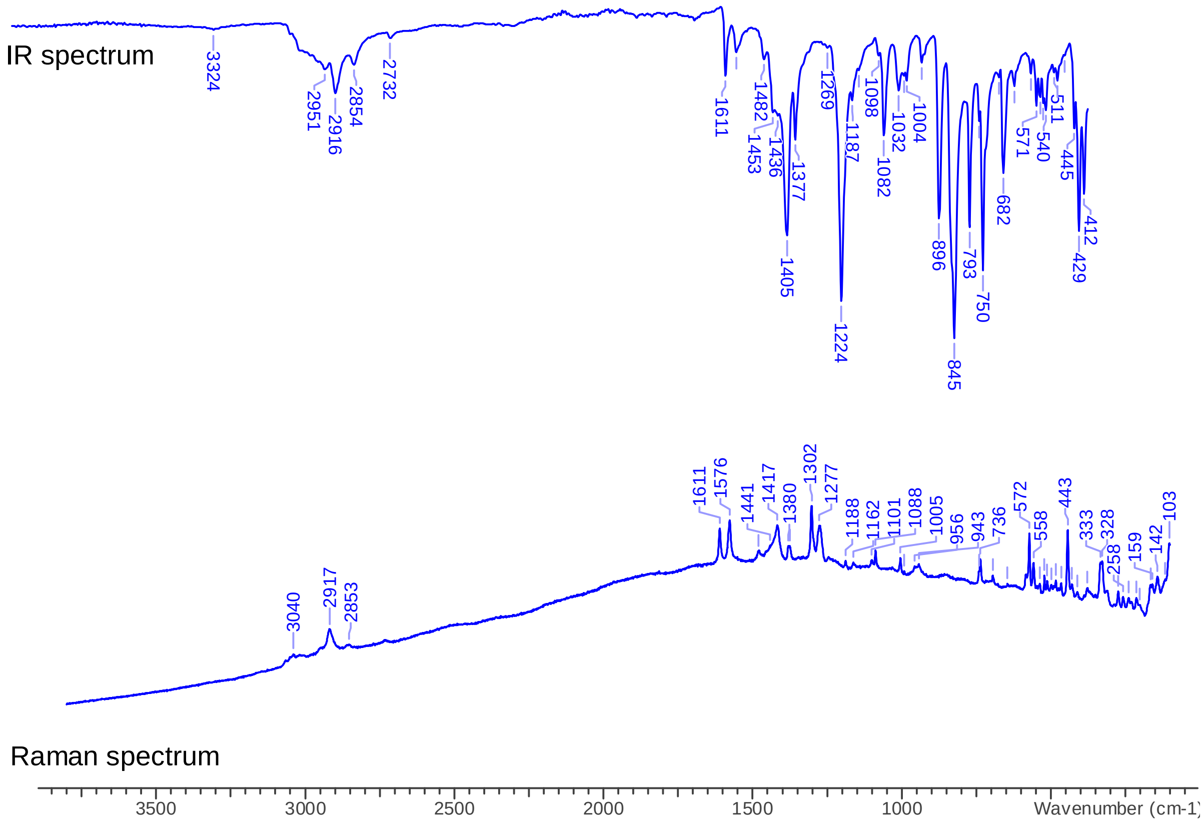


Figure S15. NMR, IR and Raman spectra of [ClP(μ-NTer)$_2$AsCl] (solvent signals are marked by an asterisk).

## 2.2 Synthesis of target biradicaloids

### 2.2.1 [P(μ-NTer)]$_2$DmpNC (11)

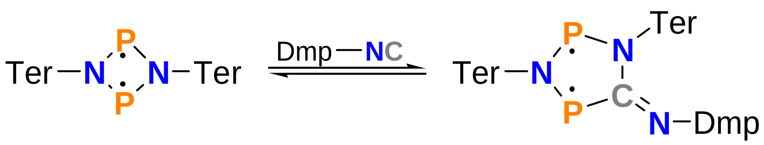


[P(μ-NTer)]$_2$DmpNC was synthesized according to our previously published synthesis procedure.[S13]

DmpNC (82 mg, 0.64 mmol) was added quickly to a stirred solution of [P(μ-NTer)]$_2$ (459 mg, 0.640 mmol) in benzene (10 mL). An immediate colour change from red to deep blue was observed. After two hours the solvent was removed in vacuo (1×10$^{-3}$ mbar) and the blue residue was dried in vacuo (1×10$^{-3}$ mbar) at 50 °C (water bath) for 30 min. The product was crystallized from a minimal amount of fresh benzene

at ambient temperature. The supernatant was removed by syringe and the crystals were dried in vacuo ($1\times10^{-3}$ mbar) at 50 °C (water bath) for 30 minutes. Yield: 480 mg (0.560 mmol, 88%).

$C_{57}H_{59}N_3P_2$ (848.07 g/mol). Mp. 207 °C. CHN calcd. (found) in %: C 80.73 (80.36), H 7.01 (6.54), N 4.95 (4.81). $^{1}H$ NMR ($C_6D_6$, 500.1 MHz): $\delta$ = 1.68 (s, 6 H, $CH_3$), 1.72 (s, 6 H, $CH_3$), 1.94 (br s, 12 H, o-$CH_3$), 2.27 (s, 6 H, $CH_3$), 2.27 (s, 6 H, $CH_3$), 2.28 (s, 6 H, $CH_3$), 6.72-6.99 (m, 17 H, CH). $^{13}C\{^{1}H\}$ NMR ($C_6D_6$, 75.5 MHz): $\delta$ = 18.5 (s, $CH_3$), 18.6 (s, $CH_3$), 20.6 (s, $CH_3$), 20.7 (s, $CH_3$), 21.0 (s, $CH_3$), 21.0 (s, $CH_3$), 21.1 (s, $CH_3$), 121.4 (arom. C), 127.4 (arom. C), 127.6 (arom. C), 127.8 (arom. C), 128.0 (arom. C), 128.2 (arom. C), 128.3 (arom. C), 128.4 (arom. C), 128.9 (arom. C), 129.6 (arom. C), 130.8 (arom. C), 131.9 (arom. C), 135.3 (arom. C), 135.9 (arom. C), 136.5 (arom. C), 136.5 (arom. C), 136.6 (arom. C), 137.1 (arom. C), 137.5 (arom. C), 138.9 (arom. C), 140.1 (arom. C), 142.8 (arom. C), 145.6 (arom. C), 150.0 (arom. C). $^{14}N$ NMR No signals observed. $^{31}P\{^{1}H\}$ NMR ($C_6D_6$, 202.5 MHz): $\delta$ = 221.7 (d, $^{2}J(^{31}P,^{31}P)$ = 136 Hz, 1 P, NPC), 258.3 (d, $^{2}J(^{31}P,^{31}P)$ = 136 Hz, 1 P, NPN). IR (ATR, 32 scans, $cm^{-1}$): $\tilde{\nu}$ = 3444 (w), 3350 (w), 3030 (w), 2937 (m), 2912 (m), 2852 (w), 2727 (w), 2324 (w), 1815 (w), 1740 (w), 1724 (w), 1641 (w), 1610 (m), 1591 (w), 1572 (w), 1537 (m), 1477 (m), 1450 (m), 1406 (m), 1377 (m), 1342 (w), 1329 (w), 1298 (w), 1284 (w), 1271 (m), 1238 (m), 1223 (m), 1192 (m), 1165 (m), 1142 (m), 1092 (m), 1082 (m), 1030 (m), 1016 (m), 982 (m), 960 (m), 951 (m), 887 (m), 877 (m), 845 (s), 837 (s), 800 (s), 771 (m), 760 (s), 750 (s), 710 (m), 687 (m), 677 (vs), 658 (m), 652 (m), 619 (m), 600 (m), 580 (m), 573 (m), 559 (m), 548 (m), 530 (m). MS ($CI^+$, iso-butane, m/z (%)): 330 (22) $[TerNH_3]^+$, 687 (10) $(\mu\text{-NTer})_2PH^+$, 716 (100) $[P(\mu\text{-NTer})]_2^+$, 748 $[M]^+$ (2).

$^{1}H$ NMR spectrum

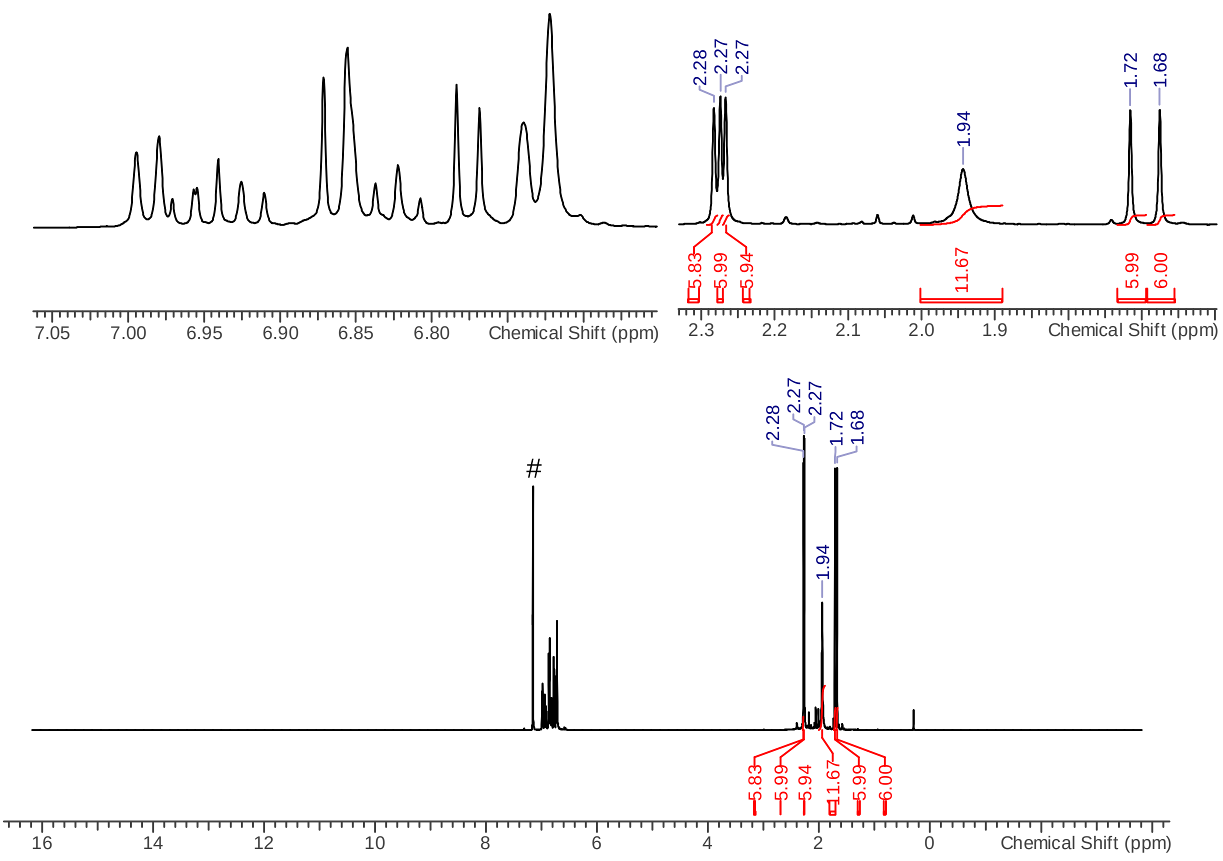


$^{13}C\{^{1}H\}$ NMR spectrum

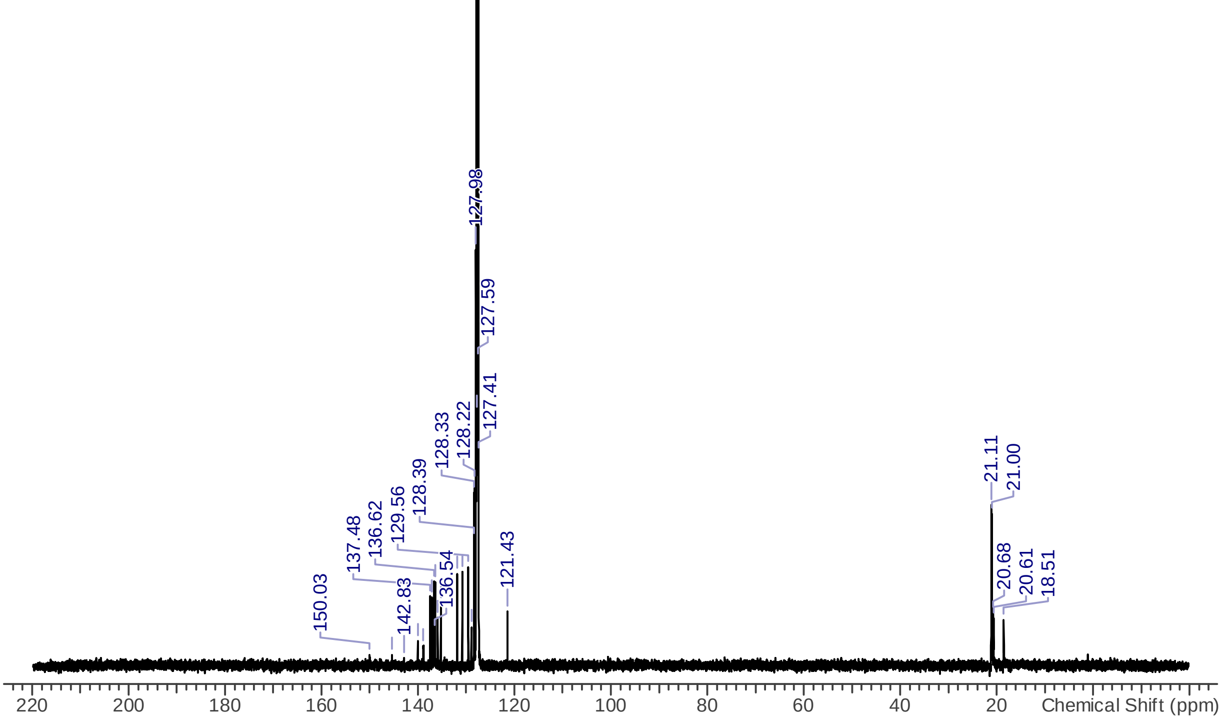

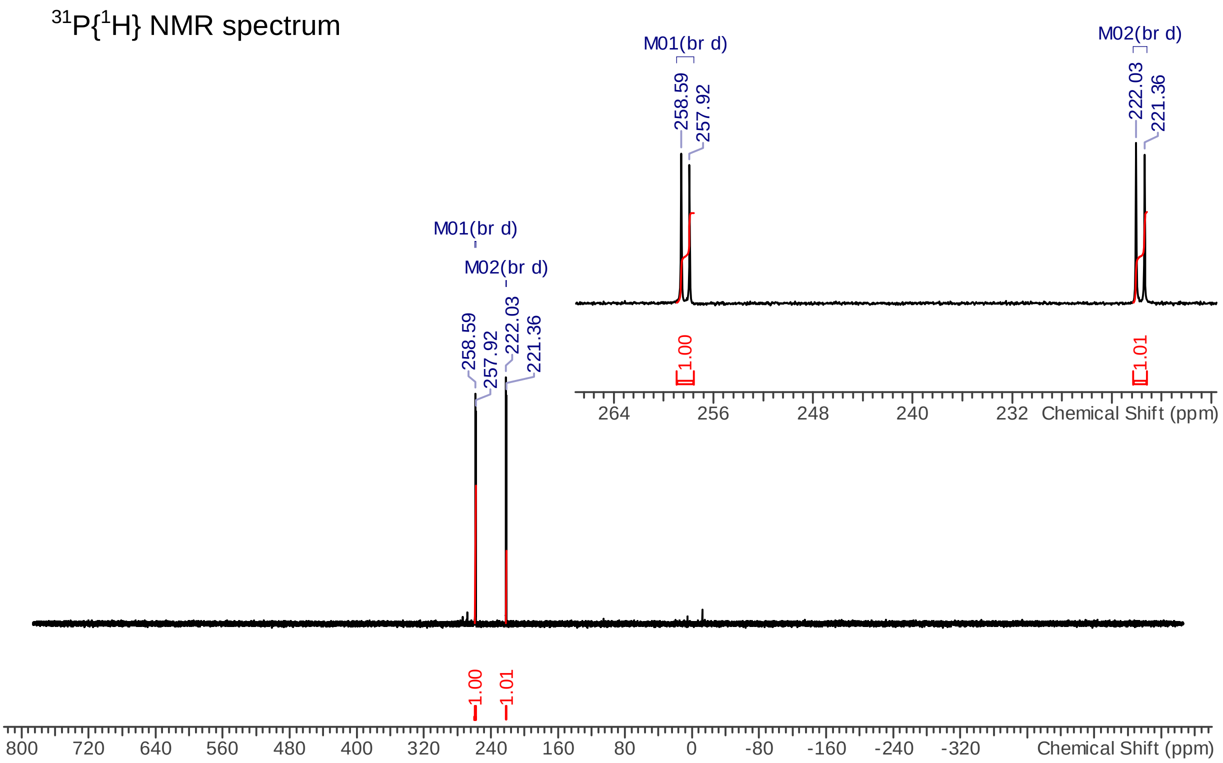


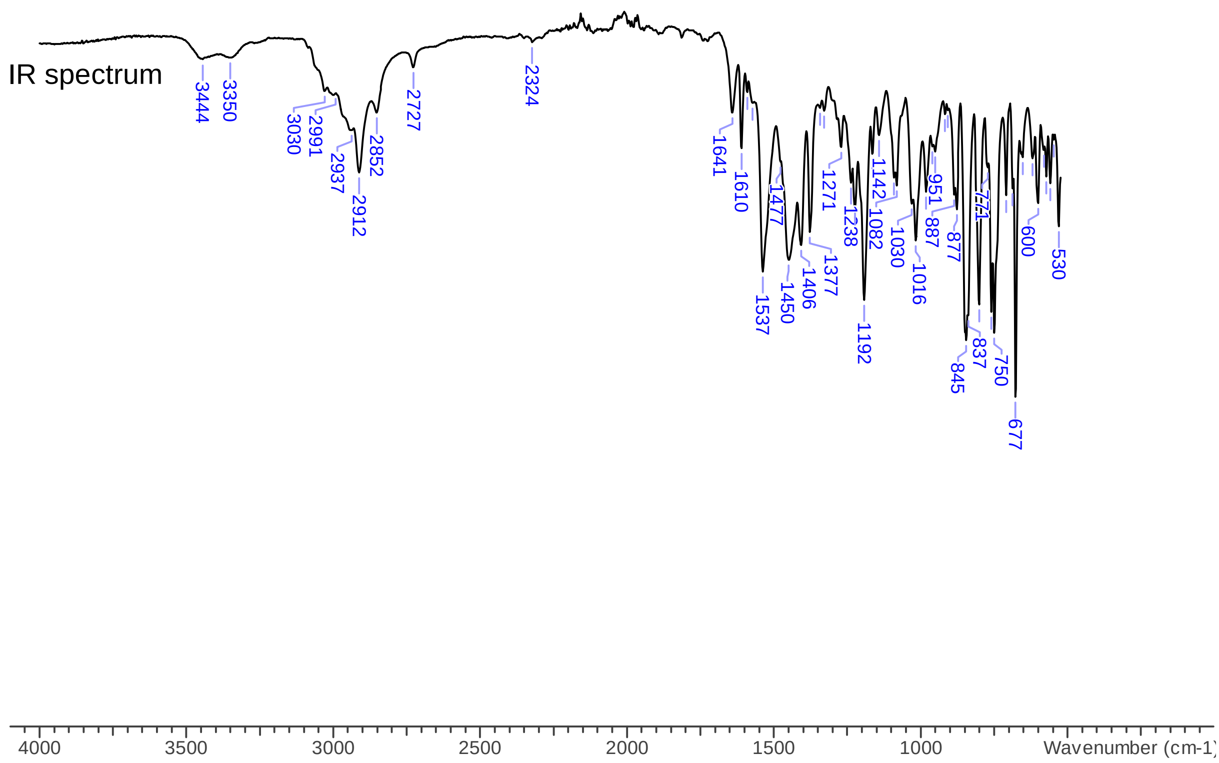


Figure S16. NMR, Raman und IR spectra of $[P(\mu\text{-}NTer)]_2DmpNC$ in $C_6D_6$ (solvent signals indicated by asterisks).

### 2.2.2 [P(µ-NTer)$_2$As] (**9**)

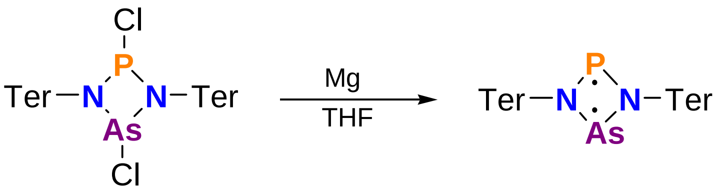


[P(µ-NTerCl)$_2$As] is synthesized according to a modified literature procedure.[S15]

Mg turnings can be activated by stirring under Argon atmosphere for several days using a glass covered magnetic stir bar.

[P(µ-NTerCl)$_2$As] (3.43 g, 4.12 mmol) and Mg turnings (4.17 g, 171 mmol) were combined in a Schlenk flask. Attention: It is paramount to ensure that no grease finds its way into the reaction vessel. THF (30 mL) was added and the reaction mixture was stirred at ambient temperature. The colorless mixture gradually turned purple. The progress of the reaction was monitored by $^{31}$P NMR spectroscopy, as over-reduction occured quickly. When the reaction was completed, the solvent was removed in vacuo ($1\times10^{-3}$ mbar), and the solid residue was dried at 40 °C (water bath) for 30 minutes. Benzene (75 mL) was added, and the insoluble material was separated by filtration. If necessary, the cloudy filtrate was filtered a second time over a celite-packed frit. The intensively purple filtrate was concentrated to incipient crystallization. After crystallization overnight at ambient temperature, purple crystals of the product were obtained. The supernatant was removed by syringe and the isolated crystals were dried in vacuo ($1\times10^{-3}$ mbar) for 30 minutes at 50 °C (water bath). Yield: 0.59 g (0.77 mmol, 19%).

$C_{48}H_{50}N_2PAs$ (760.82 g/mol). Mp. 191-203 °C (decompn.). CHN % calcd. (found): C 75.76 (76.39), H 6.62 (6.83), N 3.68 (3.45). $^{1}$H NMR ($C_6D_6$, 300.1 MHz): $\delta$ = 2.08 (s, 24 H, o-$CH_3$), 2.28 (s, 12 H, p-$CH_3$), 6.71 (s, 8 H, Mes m-CH), 6.86 (m, 4 H, Ph m-CH), 6.87 (m, 2 H, Ph p-CH). $^{13}$C{$^{1}$H} NMR ($C_6D_6$, 125.8 MHz): $\delta$ = 20.3 (s, $CH_3$), 21.5 (s, $CH_3$), 123.4 (s, arom. CH), 128.4 (s, arom. CH), 128.6 (s, arom. CH), 128.8 (s, arom. CH), 130.0 (s, arom. CH), 131.1 (d, $^{3}J(^{13}C,^{31}P)$ = 3 Hz, arom. C), 135.6 (d, $^{3}J(^{13}C,^{31}P)$ = 3 Hz, arom. C), 137.9 (s,

arom. C), 138.5 (d, $^3J(^{13}C,^{31}P)$ = 4 Hz, arom. C), 141.2 (s, arom. C). $^{14}N$ NMR no signals observed. $^{15}N$ HMBC NMR no signals observed. $^{31}P\{^1H\}$ NMR ($C_6D_6$, 121.5 MHz): $\delta$ = 268.8 (s). IR (ATR, 32 scans, $cm^{-1}$): $\tilde{\nu}$ = 3069 (m), 3055 (m), 2993 (m), 2962 (m), 2947 (m), 2914 (s), 2882 (m), 2869 (m), 2853 (m), 2824 (m), 2809 (m), 2731 (m), 1612 (m), 1579 (m), 1480 (m), 1447 (s), 1404 (s), 1377 (m), 1340 (s), 1296 (m), 1272 (m), 1237 (m), 1214 (s), 1193 (s), 1162 (m), 1111 (s), 1080 (s), 1033 (m), 1012 (m), 950 (m), 897 (s), 839 (vs), 818 (m), 796 (s), 767 (s), 752 (m), 736 (m), 699 (m), 676 (m), 585 (w), 561 (m), 540 (m), 507 (m), 470 (m), 429 (w). Raman (785 nm, 10 s, 10 scans, $cm^{-1}$): $\tilde{\nu}$ = 1612 (1), 1424 (1), 1421 (1), 1381 (1), 1380 (1), 1375 (1), 1305 (2), 1296 (1), 1295 (1), 1287 (2), 1187 (1), 1086 (1), 1005 (1), 846 (1), 819 (3), 805 (1), 793 (1), 771 (1), 763 (2), 748 (2), 746 (2), 742 (1), 740 (1), 737 (1), 733 (1), 700 (1), 692 (3), 676 (1), 587 (1), 582 (1), 580 (1), 577 (1), 570 (4), 565 (1), 558 (2), 547 (1), 537 (1), 520 (1), 507 (2), 480 (2), 434 (9), 417 (4), 407 (1), 403 (1), 378 (1), 367 (1), 352 (3), 337 (1), 332 (1), 329 (1), 327 (1), 318 (1), 300 (4), 288 (1), 277 (1), 253 (2), 242 (1), 237 (2), 232 (2), 230 (1), 227 (1), 226 (1), 213 (2), 199 (1), 197 (1), 169 (4), 155 (2), 151 (2), 141 (3), 131 (2), 129 (2), 113 (3), 94 (8), 71 (10). MS (CI, pos., isobutane) m/z (%): 73 (20), 330 (18) $[TerNH_3]^+$, 687 (11) $[Ter_2N_2PH_2]^+$, 760 (100) $[M]^+$.

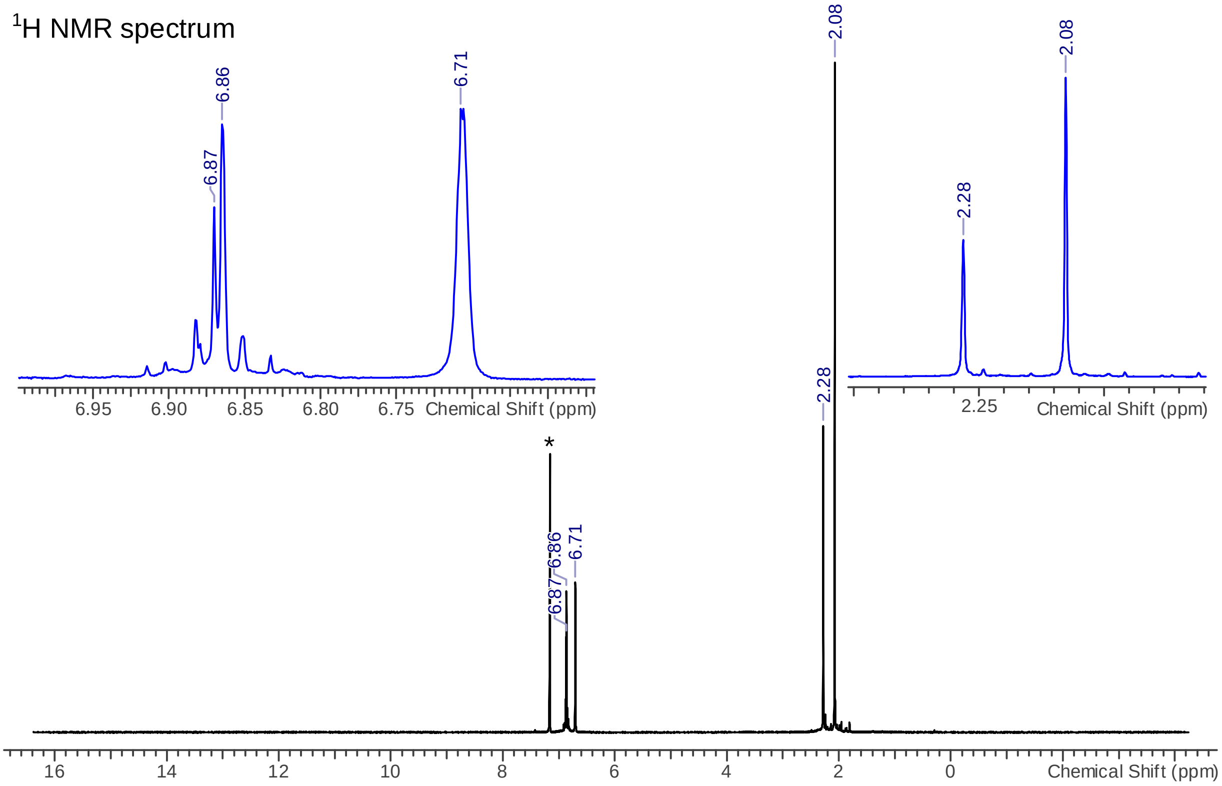

1H NMR spectrum
6.87
6.86
6.71
2.28
2.08
Chemical Shift (ppm)


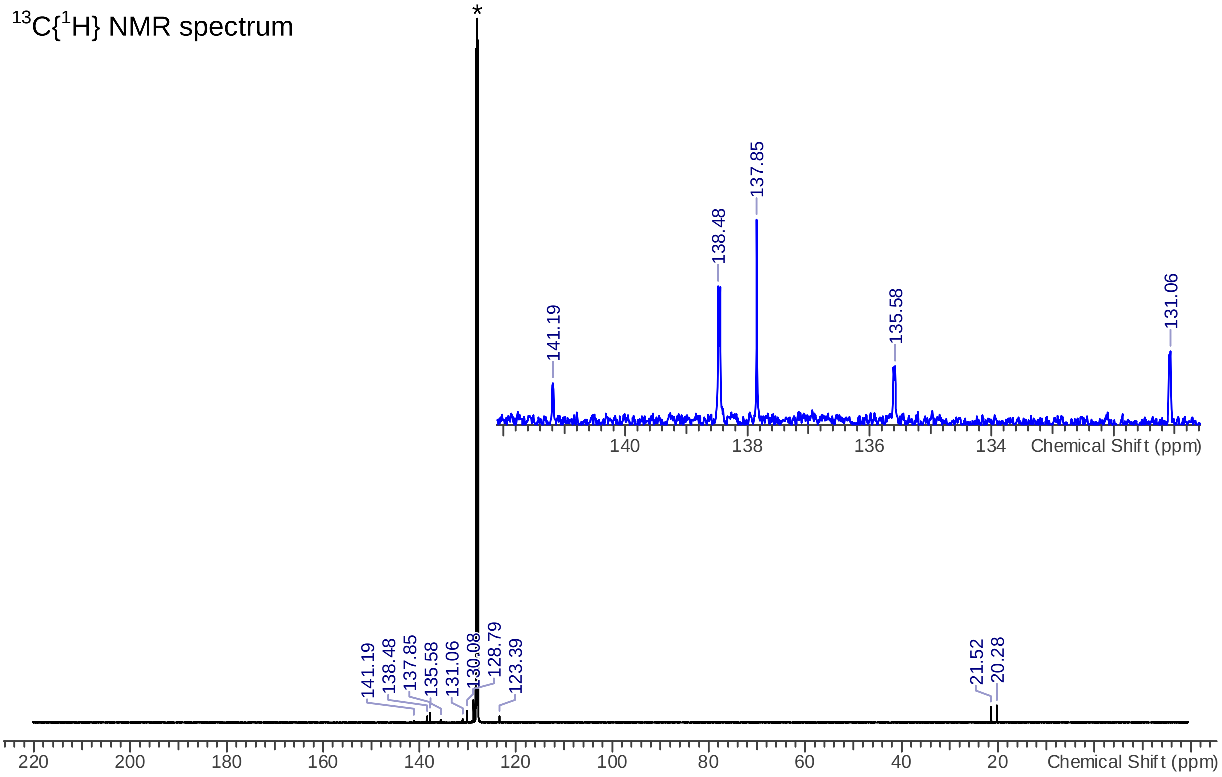

13C{1H} NMR spectrum
141.19
138.48
137.85
135.58
131.06
130.08
128.79
123.39
21.52
20.28
Chemical Shift (ppm)

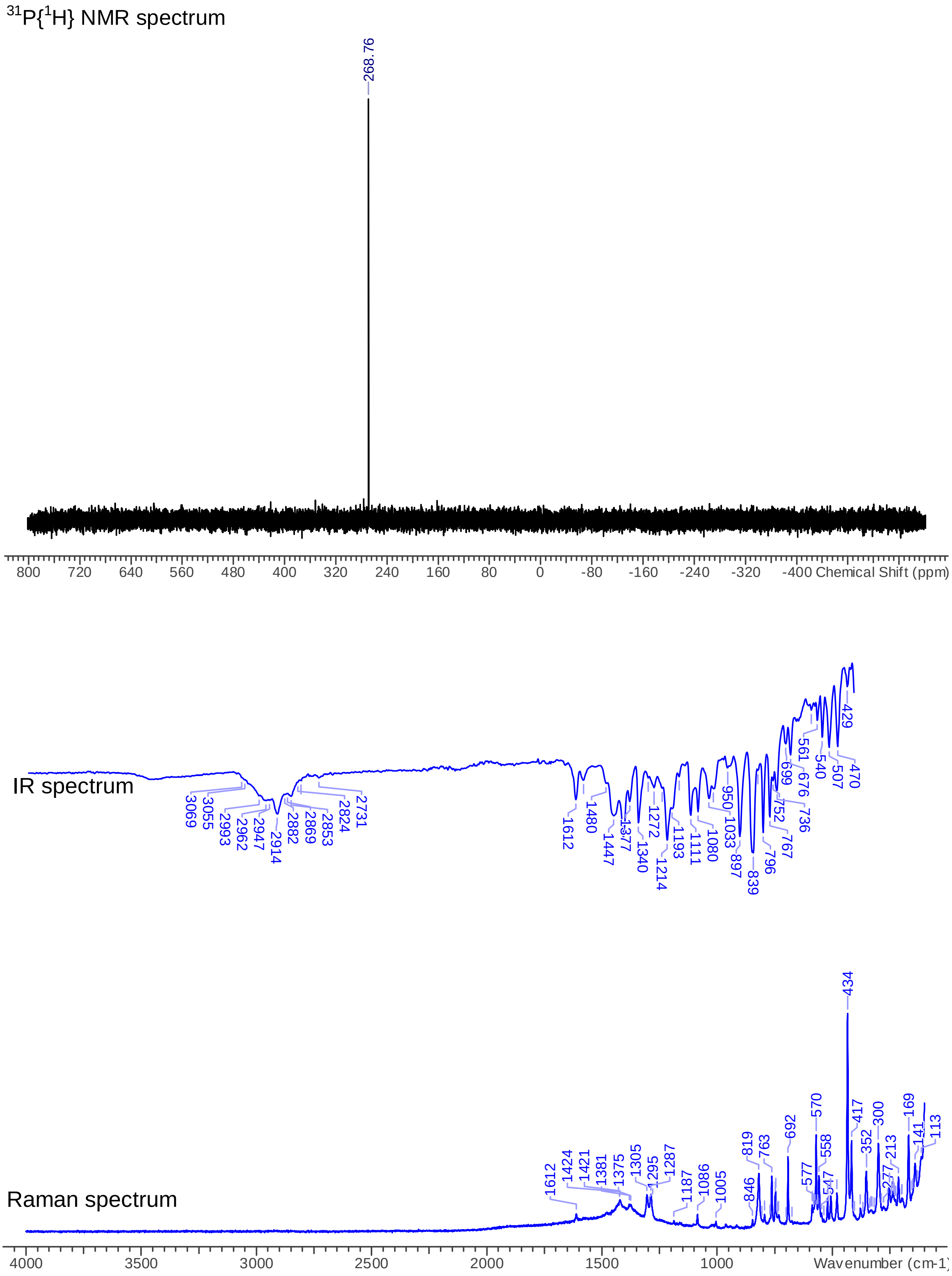


Figure S17. NMR, Raman und IR spectra of [P(μ−NTer)$_2$As] in $C_6D_6$ (solvent signals indicated by asterisks).

### 2.2.3 [P(μ-NTer)$_2$As]DmpNC (**12**)

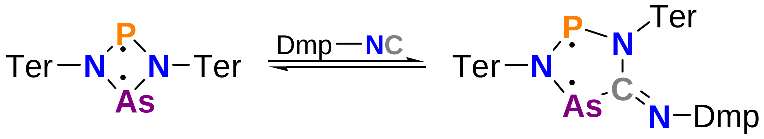

[P(μ-NTer)$_2$As]DmpNC was synthesized according to a modified literature procedure.[S16]

DmpNC (33 mg, 0.25 mmol) was added quickly to a stirred solution of [P(μ-NTer)$_2$As] (171 mg, 0.220 mmol) in benzene (5 mL). An immediate colour change from purple to dark green was observed. After two hours the solvent was removed in vacuo ($1\times10^{-3}$ mbar) and the green residue was dried in vacuo ($1\times10^{-3}$ mbar) at 50 °C (water bath) for 30 min. The product was crystallized from a minimal amount of fresh toluene at ambient temperature. The supernatant was removed by syringe and the crystals were dried in vacuo ($1\times10^{-3}$ mbar) at 50 °C (water bath) for 30 minutes. Yield: 116 mg (0.130 mmol, 58%).

$C_{57}H_{59}AsN_3P$ (891.99 g/mol). Mp. 120 °C (decompn.). $^1$H NMR ($C_6D_6$, 500.1 MHz): $\delta$ = 1.71 (s, 6 H, $CH_3$), 1.73 (s, 6 H, $CH_3$), 1.96 (s, 12 H, $CH_3$), 2.25 (s, 6 H, $CH_3$), 2.28 (s, 6 H, $CH_3$), 2.31 (s, 6 H, $CH_3$), 6.72-6.99 (m, 17 H, arom. CH). $^{13}$C{$^1$H} NMR ($C_6D_6$, 125.8 MHz): $\delta$ = 21.1 (s, $CH_3$), 21.1 (s, $CH_3$), 21.3 (s, $CH_3$), 21.4 (s, $CH_3$), 21.4 (s, $CH_3$), 21.6 (s, $CH_3$), 122.3 (s, arom. CH), 127.9 (s, arom. CH), 128.0 (s, arom. CH), 128.4 (s, arom. CH), 128.8 (s, arom. CH), 129.1 (s, arom. CH), 129.9 (s, arom. CH), 131.4 (s, arom. CH), 132.3 (s, arom. CH), 136.1 (s, arom. C), 136.5 (s, arom. C), 136.9 (s, arom. C), 137.1 (s, arom. C), 137.2 (s, arom. C), 137.6 (s, arom. C), 138.0 (s, arom. C), 138.0 (s, arom. C), 140.9 (d, J = 3 Hz, arom. C), 151.1 (s, arom. C).$^{14}$N NMR no signals observed. $^{15}$N HMBC NMR no signals observed. $^{31}$P{$^1$H} NMR ($C_6D_6$, 202.5 MHz): $\delta$ = 268.9 (s). IR (ATR, 32 scans, cm$^{-1}$): $\tilde{\nu}$ = 3090 (m), 3069 (m), 3055 (m), 3032 (m), 2993 (m), 2964 (m), 2947 (s), 2914 (s), 2853 (m), 2816 (m), 1639 (s), 1612 (s), 1581 (m), 1478 (m), 1439 (s), 1406 (s), 1375 (s), 1336 (vs), 1268 (s), 1237 (s), 1208 (s), 1189 (s), 1162 (m), 1130 (m), 1117 (m), 1094 (s), 1080 (s), 1031 (m), 1016 (m), 983 (m), 845 (vs), 791 (s), 765 (s), 752 (s), 676 (s), 559 (m), 534 (m), 521 (m), 476 (w).

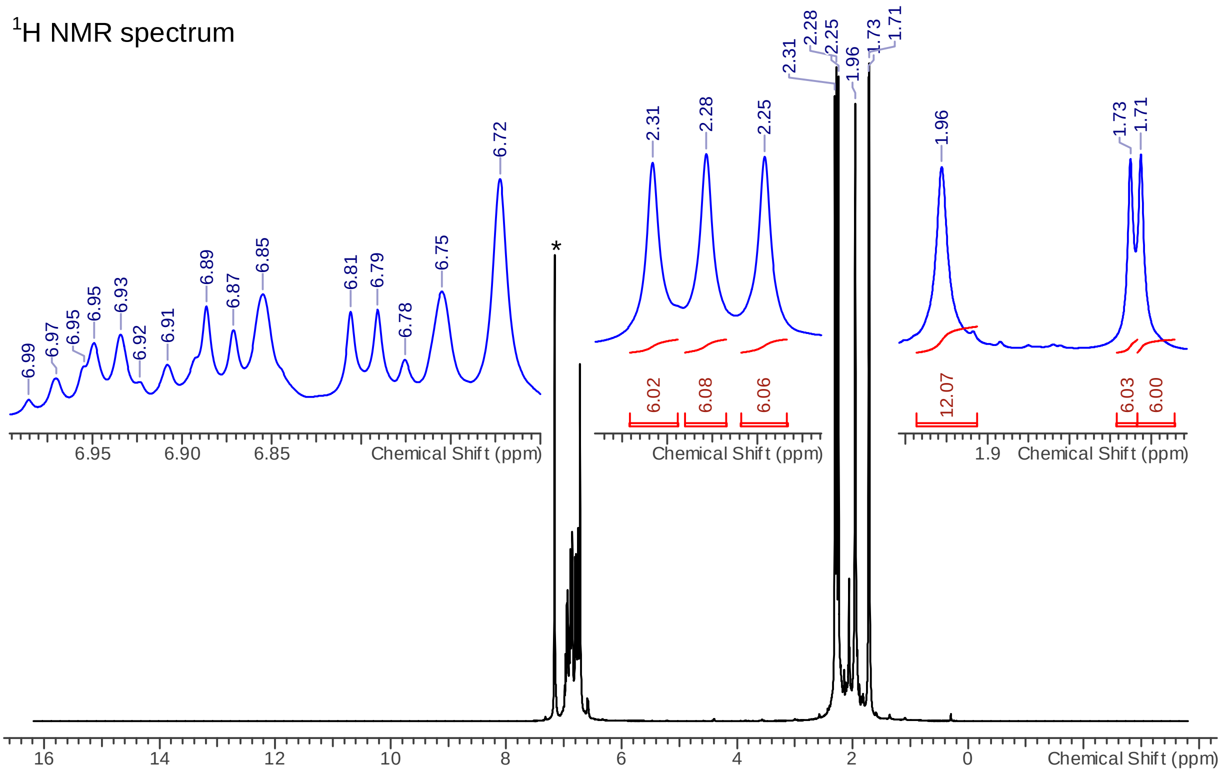
1H NMR spectrum
6.99
6.97
6.95
6.95
6.93
6.92
6.91
6.89
6.87
6.85
6.81
6.79
6.78
6.75
6.72
2.31
2.28
2.25
6.02
6.08
6.06
1.96
1.73
1.71
12.07
6.03
6.00
*
Chemical Shift (ppm)

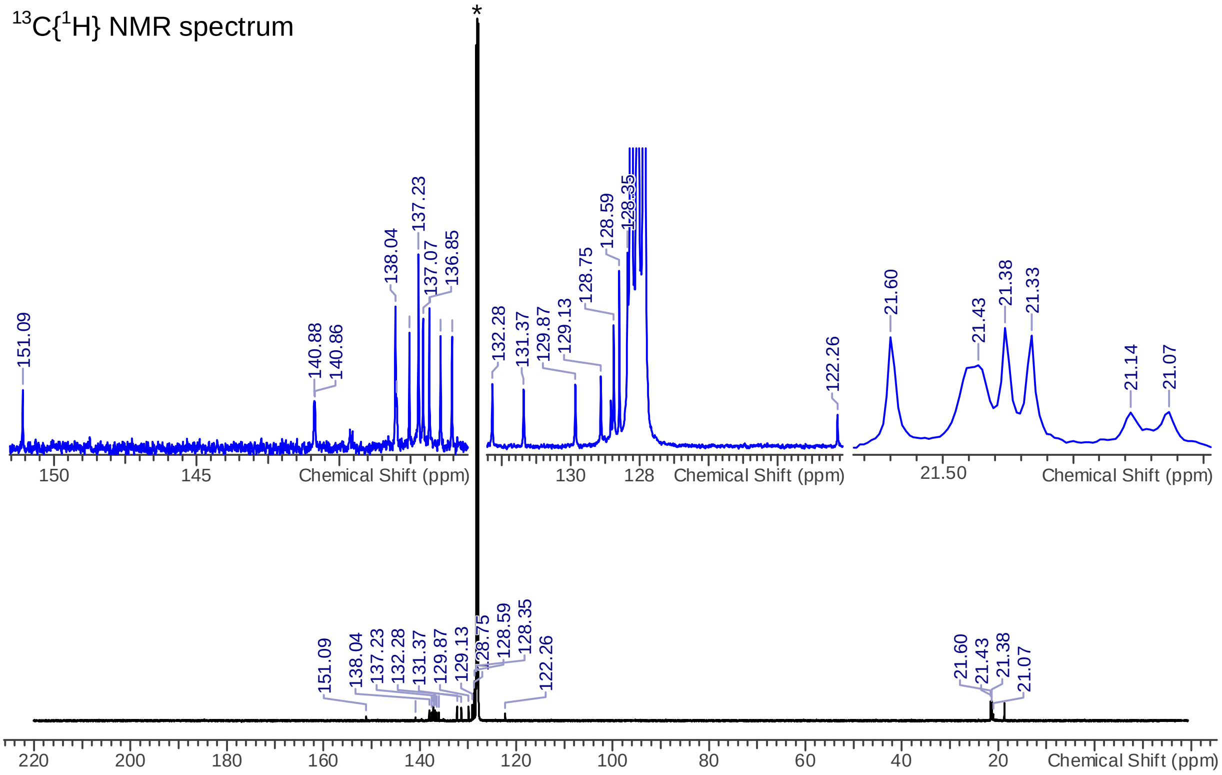
13C{1H} NMR spectrum
*
151.09
140.88
140.86
138.04
137.23
137.07
136.85
132.28
131.37
129.87
129.13
128.75
128.59
128.35
122.26
21.60
21.43
21.38
21.33
21.14
21.07
Chemical Shift (ppm)

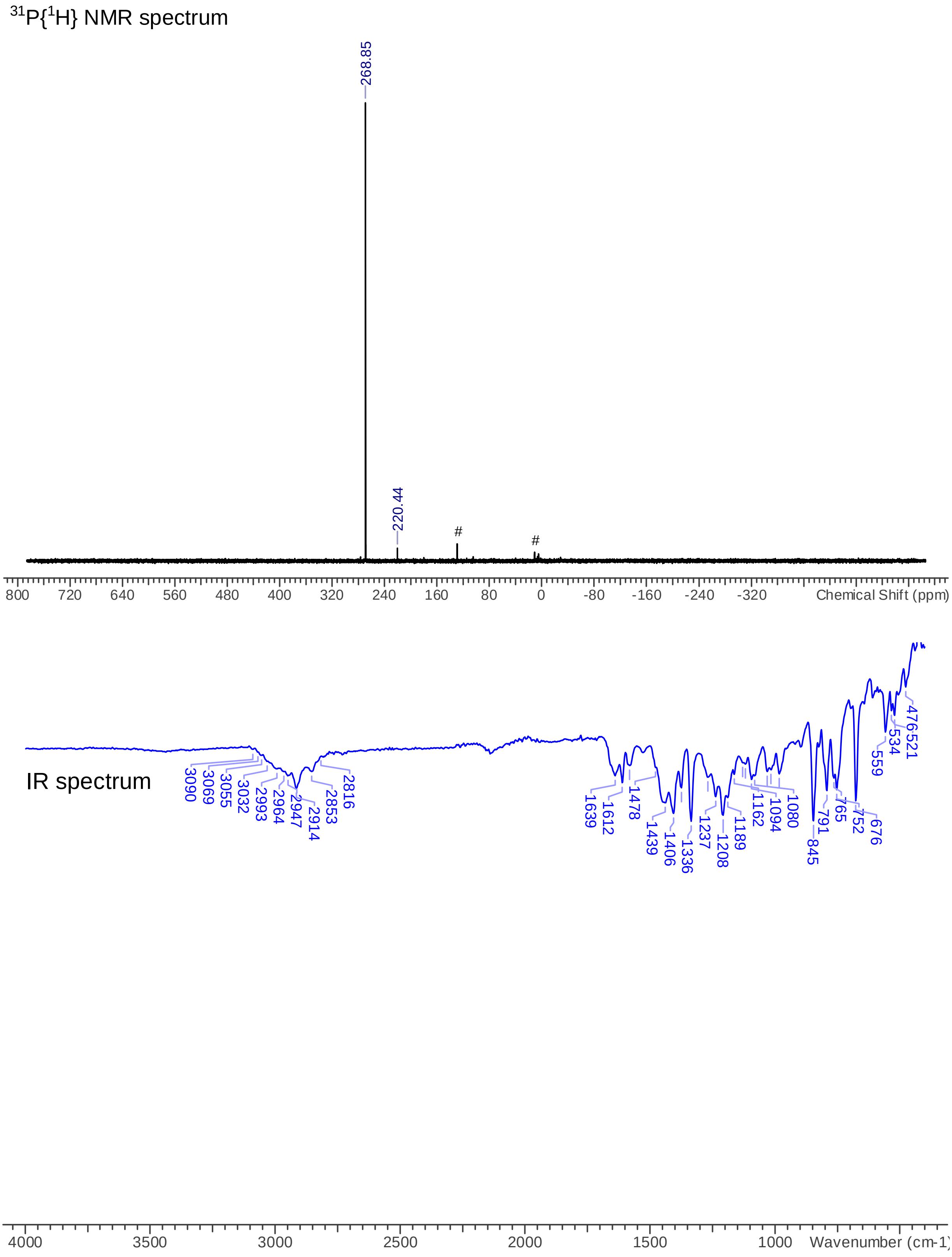


Figure S18. NMR and IR spectra of [P(μ−NTer)$_2$As]DmpNC in $C_6D_6$ (solvent signals indicated by asterisks).

### 2.2.4 Attempted synthesis of [As(μ-NTer)]$_2$DmpNC

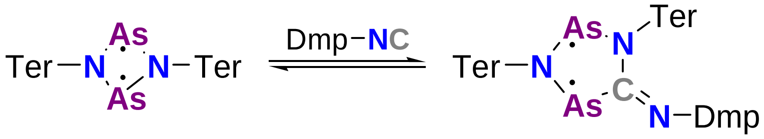


DmpNC (6 mg, 0.05 mmol) and [As(μ-NTer)]$_2$ (30 mg, 0.039 mmol) were combined in a J. YOUNG NMR tube and were dissolved in $C_6D_6$ (0.5 mL). The solution was left in an ultrasonic bath for 15 min prior to analysis. No colour change was observed.

The evaluation of the NMR spectra showed no reaction between the two starting materials. Only the signals of [As($\mu$−NTer)]$_2$ and DmpNC were detectable.

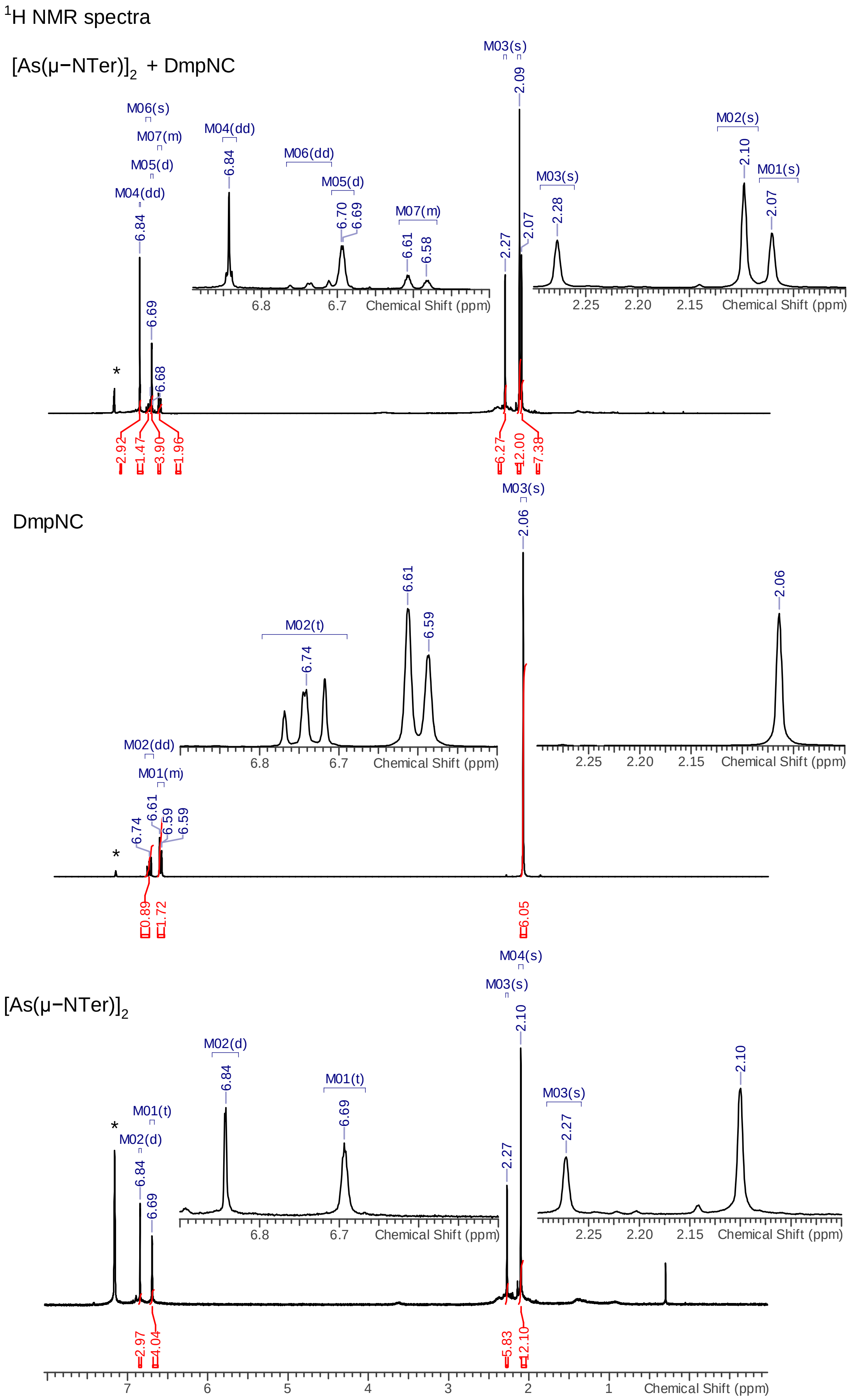


Figure S19. NMR spectra of $[As(\mu{-}NTer)]_2$ and DmpNC in $C_6D_6$ (solvent signals indicated by asterisks).

# 3 Computational Details

Computations were carried out using Gaussian09[S17] or ORCA 4.2.1.[S18]

Structure optimizations employed the pure DFT exchange-correlation functional PBE[S19,20] in conjunction with Grimme's dispersion correction D3(BJ)[S21,22] and the def2-TZVP basis set[S23] (notation PBE-D3/def2-TZVP). All structures were fully optimized and confirmed as minima or transition states by frequency analyses. Chemical shifts and coupling constants were derived by the GIAO method at the PBE0-D3/def2-TZVP level of theory.[S24-29] The calculated absolute shifts ($\sigma_{calc,X}$) were referenced to the experimental absolute shift of 85% $H_3PO_4$ in the gas phase ($\sigma_{ref,1}$ = 328.35 ppm),[S30] using $PH_3$ ($\sigma_{ref,2}$ = 594.45 ppm) as a secondary standard:[S31]

$$\begin{aligned}\delta_{calc,X} &= (\sigma_{ref,1} - \sigma_{ref,2}) - (\sigma_{calc,X} - \sigma_{calc,PH_3}) \\ &= \sigma_{calc,PH_3} - \sigma_{calc,X} - 266.1\ \text{ppm}\end{aligned}$$

At the PBE0-D3/def2-TZVP level of theory, $\sigma_{calc,PH_3}$ amounts to +572.04 ppm.

More accurate electronic energies for optimized structures were computed by single-point DLPNO-CCSD(T)[S32-35] calculations employing the def2-TZVP basis set[S23] and def2-TZVP/C correlation fitting basis[S36] (notation: DLPNO-CCSD(T)/def2-TZVP//PBE-D3/def2-TZVP). Thermodynamic quantities at this level of theory were calculated using the DLPNO-CCSD(T) single point energy and the thermal corrections at the PBE-D3/def2-SVP level of theory. The $T_1$ diagnostic was evaluated to ensure reliable results (empirically, CCSD(T) results are considered reliable if $T_1 < 0.02$).[S37]

Please note that all computations were carried out for single, isolated molecules in the gas phase (ideal gas approximation). There may well be significant differences between gas phase and condensed phase.

## 3.1 Summary of calculated data

### 3.1.1 Thermodynamic data

Table S3. Summary of calculated data, including electronic energies and thermal corrections. TS = transition state.

| Compd. | PG | Opt. method | $E_{tot}$[a] | ΔG[b] | $E_{CCSD(T)}$[c] | $T_1$ |
|---|---|---|---|---|---|---|
| $[TerNP]_2$ | $D_2$ | PBE-D3/ def2-TZVP | −2649.5088 | 0.7310 | −2646.8677 | 0.010 |
| $[TerN]_2PAs$ | $C_2$ | | −4543.8479 | 0.7273 | −4540.4304 | 0.011 |
| $[TerNAs]_2$ | $D_2$ | | −6438.1828 | 0.7252 | −6433.9943 | 0.010 |
| $[TerNP]_2DmpNC$ | $C_1$ | | −3052.2956 | 0.8715 | −3049.2398 | 0.011 |
| $[TerN]_2PAsDmpNC$ | $C_1$ | | −4946.6315 | 0.8699 | −4942.8011 | 0.012 |
| DmpNC | $C_{2v}$ | | −402.7344 | 0.1151 | −402.3334 | 0.011 |
| $H_2$ | $D_{\infty}h$ | | −1.1661 | −0.0017 | −1.1684 | 0.006 |
| $[TerNAs]_2DmpNC$ | $C_1$ | | −6840.9600 | 0.8652 | −6836.3569 | 0.011 |
| $[TerNP]_2 \cdot H_2$_TS | $C_1$ | | −2650.6689 | 0.7415 | −2648.0181 | 0.010 |
| $[TerN]_2PAs \cdot H_2$_TS | $C_1$ | | −4545.0065 | 0.7381 | −4541.5821 | 0.010 |
| $[TerNAs]_2 \cdot H_2$_TS | $C_1$ | | −6439.3419 | 0.7379 | −6435.1451 | 0.010 |
| $[TerNP]_2DmpNC \cdot H_2$_TS | $C_1$ | | −3053.4565 | 0.8855 | −3050.3927 | 0.010 |
| $[TerN]_2PAsDmpNC \cdot H_2$_TS | $C_1$ | | −4947.7941 | 0.8849 | −4943.9581 | 0.011 |
| $[TerNP]_2 \cdot H_2$ | $C_2$ | | −2650.6991 | 0.7504 | −2648.0574 | 0.010 |
| $[TerN]_2PAs \cdot H_2$ | $C_1$ | | −4545.0311 | 0.7450 | −4541.6188 | 0.010 |
| $[TerNAs]_2 \cdot H_2$ | $C_2$ | | −6439.3606 | 0.7434 | −6435.1743 | 0.010 |
| $[TerNP]_2DmpNC \cdot H_2$ | $C_1$ | | −3053.4940 | 0.8913 | −3050.4431 | 0.010 |
| $[TerN]_2PAsDmpNC \cdot H_2$ | $C_1$ | | −4947.8252 | 0.8901 | −4944.0010 | 0.010 |

[a] Total SCF energy in a.u.; [b] thermal correction to Gibbs energy in a.u. (298 K unless stated otherwise); [c] single-point DLPNO-CCSD(T)/def2-TZVP energy.

### 3.1.2 Reaction energies

Table S4: Reaction energies (DLPNO-CCSD(T)/def2-TZVP//PBE-D3/def2-TZVP, in kJ·mol$^{-1}$, c° = 1 mol/L).

| Reaction | $\Delta E^{tot}$ | $\Delta_R H$ | $\Delta_R G^o$ |
|---|---|---|---|
| $[TerNP]_2 + H_2 \rightarrow [TerNP]_2 \cdot H_2$ | −56.0 | −44.4 | −8.5 |
| $[TerN]_2PAs + H_2 \rightarrow [TerN]_2PAs \cdot H_2$ | −52.6 | −43.0 | −9.8 |
| $[TerNAs]_2 + H_2 \rightarrow [TerNAs]_2 \cdot H_2$ | −30.4 | −22.6 | +13.9 |
| $[TerNP]_2DmpNC + H_2 \rightarrow [TerNP]_2DmpNC \cdot H_2$ | −91.7 | −77.6 | −43.3 |
| $[TerN]_2PAsDmpNC + H_2 \rightarrow [TerN]_2PAsDmpNC \cdot H_2$ | −82.8 | −70.4 | −33.5 |
| $[TerNAs]_2$+ DmpNC → $[TerNAs]_2DmpNC$ | −76.7 | −69.1 | −19.1 |
| $[TerN]_2PAs$ + DmpNC → $[TerN]_2PAsDmpNC$ | −98.0 | −89.6 | −33.7 |
| $[TerNP]_2$ + DmpNC → $[TerNP]_2DmpNC$ | −101.4 | −93.2 | −42.6 |

Table S5: Activation energies (DLPNO-CCSD(T)/def2-TZVP//PBE-D3/def2-TZVP, in kJ·mol$^{-1}$, c° = 1 mol/L).

| Process | $\Delta E^{tot}$ | $\Delta H^{\ddagger}$ | $\Delta G^{\ddagger}$ |
|---|---|---|---|
| $[TerNP]_2 + H_2 \rightarrow [TerNP]_2 \cdot H_2$_TS | 47.3 | 47.3 | 71.5 |
| $[TerN]_2PAs + H_2 \rightarrow [TerN]_2PAs \cdot H_2$_TS | 43.9 | 43.1 | 68.5 |
| $[TerNAs]_2 + H_2 \rightarrow [TerNAs]_2 \cdot H_2$_TS | 46.2 | 44.6 | 76.2 |
| $[TerNP]_2DmpNC + H_2 \rightarrow [TerNP]_2DmpNC \cdot H_2$_TS | 40.6 | 39.2 | 73.8 |
| $[TerN]_2PAsDmpNC + H_2 \rightarrow [TerN]_2PAsDmpNC \cdot H_2$_TS | 29.8 | 28.1 | 65.5 |
| $[TerNP]_2 \cdot H_2 \rightarrow [TerNP]_2 \cdot H_2$_TS | 103.3 | 91.7 | 79.9 |
| $[TerN]_2PAs \cdot H_2 \rightarrow [TerN]_2PAs \cdot H_2$_TS | 96.5 | 86.0 | 78.3 |
| $[TerNAs]_2 \cdot H_2 \rightarrow [TerNAs]_2 \cdot H_2$_TS | 76.6 | 67.2 | 62.3 |
| $[TerNP]_2DmpNC \cdot H_2 \rightarrow [TerNP]_2DmpNC \cdot H_2$_TS | 132.3 | 116.8 | 117.0 |
| $[TerN]_2PAsDmpNC \cdot H_2 \rightarrow [TerN]_2PAsDmpNC \cdot H_2$_TS | 112.6 | 98.5 | 99.0 |

### 3.1.3 Calculated NMR Shifts and coupling constants

The following tables contain experimentally observed $^{1}H$ and $^{31}P$ NMR parameters in toluene-$d_8$ at 298 K. Calculated values are given in brackets for comparison (GIAO method, PBE0-D3/def2-TZVP).

Table S6. $^{1}H$ and $^{31}P$ NMR data of $[TerNP]_2{\cdot}H_2$ (8-$H_2$). Calculated values (GIAO method) are given in brackets.

| | ***δ*** [ppm] | J [Hz] | | |
|---|---|---|---|---|
| | | A | X | |
| A ($^{1}H$) | 7.04<br>(6.93) | $J_{AA'}$ = 5.9 (1.3) | – | 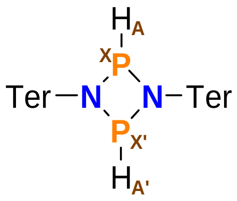 |
| X ($^{31}P$) | 194.0<br>(175.2) | $J_{AX}$ = 129.8 (108.5)<br>$J_{AX'}$ = 6.1 (−6.7) | $J_{XX'}$ = 21.7 (−3.0) | |

Table S7. $^{1}H$ and $^{31}P$ NMR data of $[TerN]_2PAs{\cdot}H_2$ (9-$H_2$). Calculated values (GIAO method) are given in brackets.

| | ***δ*** [ppm] | J [Hz] | | | |
|---|---|---|---|---|---|
| | | A | B | M | |
| A ($^{1}H$) | 6.91<br>(6.86) | – | – | – | 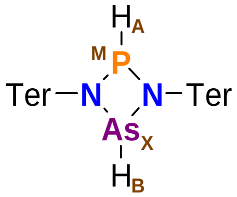 |
| B ($^{1}H$) | 8.36<br>(8.10) | $J_{AB}$ = 5.25 (6.1) | – | – | |
| M ($^{31}P$) | 204.1<br>(220.2) | $J_{AM}$ = 135.5<br>(102.6) | $J_{BM}$ = 5.75 (5.8) | – | |
| X ($^{75}As$) | very<br>broad | $J_{AX}$ = invisible<br>(1.9) | $J_{BX}$ = invisible<br>(−48.0) | $J_{MX}$ = invisible<br>(4.2) | |

Table S8. $^{1}$H and $^{75}$As NMR data of $[TerNAs]_2{\cdot}H_2$ (10-$H_2$). Calculated values (GIAO method) are given in brackets.

| | $\delta$ [ppm] | J [Hz] A | X | |
|---|---|---|---|---|
| A ($^{1}$H) | 8.07 (6.85) | $J_{AA'}$ = invisible (0.2) | – | 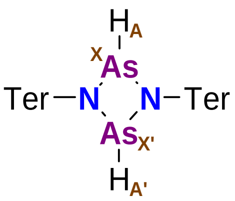 |
| X ($^{75}$As) | very broad | $J_{AX}$ = invisible (−10.5)<br>$J_{AX'}$ = invisible (−6.3) | $J_{XX'}$ = invisible (−7.1) | |



Table S9. $^{1}$H and $^{31}$P NMR data of $[TerNP]_2DmpNC{\cdot}H_2$ (11-$H_2$). Calculated values (GIAO method) are given in brackets.

| | $\delta$ [ppm] | J [Hz] A | B | M | |
|---|---|---|---|---|---|
| A ($^{1}$H) | 5.08 (5.88) | – | – | – | 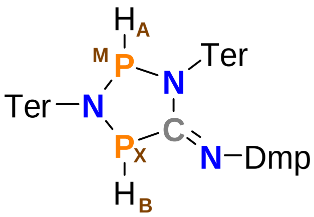 |
| B ($^{1}$H) | 4.20 (4.96) | $J_{AB}$ = 2.5 (2.2) | – | – | |
| M ($^{31}$P) | 105.9 (113.8) | $J_{AM}$ = 202.2 (148.6) | $J_{BM}$ = 9.6 (5.9) | – | |
| X ($^{31}$P) | 20.2 (32.9) | $J_{AX}$ = 4.1 (2.8) | $J_{BX}$ = 169.7 (−128.1) | $J_{XX}$ = invisible (−20.7) | |



Table S10. $^{1}$H, $^{31}$P and $^{75}$As NMR data of $[TerN]_2PAsDmpNC{\cdot}H_2$ (12-$H_2$). Calculated values (GIAO method) are given in brackets.

| | $\delta$ [ppm] | J [Hz] A | B | M | |
|---|---|---|---|---|---|
| A ($^{1}$H) | 5.15 (5.18) | – | – | – | 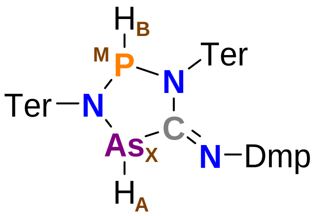 |
| B ($^{1}$H) | 5.39 (6.02) | $J_{AB}$ = ≤1, not resolved (2.5) | – | – | |
| M ($^{31}$P) | 113.3 (122.5) | $J_{AM}$ = 4.2 (5.3) | $J_{BM}$ = 205.7 (−156.8) | – | |
| X ($^{75}$As) | very broad | $J_{AX}$ = invisible (15.0) | $J_{BX}$ = invisible (1.4) | $J_{MX}$ = invisible (−18.6) | |

## 3.2 Optimized structures (.xyz files)

### 3.2.1 DmpNC (PBE-D3/def2-TZVP)

```
19
DmpNC PBE-D3/def2-TZVP
N          0.00000          0.00000          1.81988
C          0.00000          0.00000          3.00232
C          0.00000          0.00000          0.43768
C          0.00000          1.23868         -0.23729
C         -0.00000         -1.23868         -0.23729
C          0.00000          1.20977         -1.63449
C         -0.00000         -1.20977         -1.63449
C         -0.00000          0.00000         -2.32880
H          0.00000          2.15464         -2.18095
H         -0.00000         -2.15464         -2.18095
H         -0.00000          0.00000         -3.41970
C          0.00000          2.52635          0.53187
H         -0.88091          2.59966          1.18744
H          0.88091          2.59966          1.18744
H          0.00000          3.38716         -0.14778
C         -0.00000         -2.52635          0.53187
H          0.88091         -2.59966          1.18744
H         -0.88091         -2.59966          1.18744
H         -0.00000         -3.38716         -0.147787
```

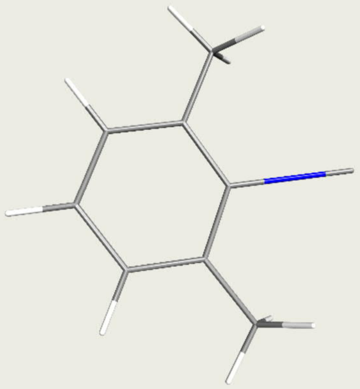

### 3.2.2 $[TerNP]_2$ (PBE-D3/def2-TZVP)

```
102
[TerNP]2 PBE-D3/def2-TZVP
N          0.00000          0.00000         -1.11373
N         -0.00000          0.00000          1.11373
P          1.34196         -0.00000          0.00000
P         -1.34196          0.00000          0.00000
C         -0.00000          0.00000          2.51578
C          1.15151          0.41355          3.22973
C         -1.15151         -0.41355          3.22973
C          1.13067          0.40425          4.62874
C         -1.13067         -0.40425          4.62874
C         -0.00000          0.00000          5.33559
H          2.02588          0.73427          5.15965
H         -2.02588         -0.73427          5.15965
H         -0.00000          0.00000          6.42631
C          0.00000          0.00000         -2.51578
C          1.15151         -0.41355         -3.22973
C         -1.15151          0.41355         -3.22973
C          1.13067         -0.40425         -4.62874
C         -1.13067          0.40425         -4.62874
C          0.00000          0.00000         -5.33559
H          2.02588         -0.73427         -5.15965
H         -2.02588          0.73427         -5.15965
H          0.00000          0.00000         -6.42631
C         -2.38012          0.88834         -2.53306
C         -3.49607          0.03671         -2.39705
C         -2.43031          2.21065         -2.03345
C         -4.64192          0.52264         -1.75763
```

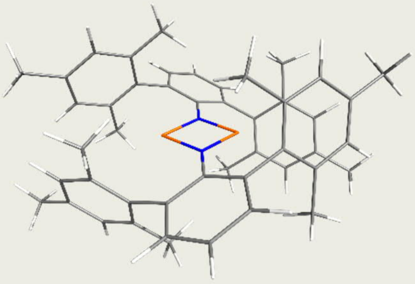

| | | | |
|---|---|---|---|
| C | -3.59518 | 2.65340 | -1.40493 |
| C | -4.71076 | 1.82189 | -1.25072 |
| H | -5.50022 | -0.14316 | -1.63783 |
| H | -3.62778 | 3.67377 | -1.01271 |
| C | 2.38012 | -0.88834 | -2.53306 |
| C | 3.49607 | -0.03671 | -2.39705 |
| C | 2.43031 | -2.21065 | -2.03345 |
| C | 4.64192 | -0.52264 | -1.75763 |
| C | 3.59518 | -2.65340 | -1.40493 |
| C | 4.71076 | -1.82189 | -1.25072 |
| H | 5.50022 | 0.14316 | -1.63783 |
| H | 3.62778 | -3.67377 | -1.01271 |
| C | -2.38012 | -0.88834 | 2.53306 |
| C | -3.49607 | -0.03671 | 2.39705 |
| C | -2.43031 | -2.21065 | 2.03345 |
| C | -4.64192 | -0.52264 | 1.75763 |
| C | -3.59518 | -2.65340 | 1.40493 |
| C | -4.71076 | -1.82189 | 1.25072 |
| H | -5.50022 | 0.14316 | 1.63783 |
| H | -3.62778 | -3.67377 | 1.01271 |
| C | 2.38012 | 0.88834 | 2.53306 |
| C | 2.43031 | 2.21065 | 2.03345 |
| C | 3.49607 | 0.03671 | 2.39705 |
| C | 3.59518 | 2.65340 | 1.40493 |
| C | 4.64192 | 0.52264 | 1.75763 |
| C | 4.71076 | 1.82189 | 1.25072 |
| H | 3.62778 | 3.67377 | 1.01271 |
| H | 5.50022 | -0.14316 | 1.63783 |
| C | -3.44373 | 1.39325 | 2.86434 |
| H | -4.42327 | 1.87609 | 2.75125 |
| H | -3.13411 | 1.47473 | 3.91562 |
| H | -2.71238 | 1.96427 | 2.26962 |
| C | -1.23185 | -3.11331 | 2.13934 |
| H | -0.84670 | -3.15765 | 3.16852 |
| H | -1.47746 | -4.13185 | 1.81206 |
| H | -0.40856 | -2.74168 | 1.50730 |
| C | -5.94634 | -2.31922 | 0.55021 |
| H | -5.71559 | -2.64477 | -0.47593 |
| H | -6.38131 | -3.18563 | 1.07178 |
| H | -6.71607 | -1.53787 | 0.49373 |
| C | 3.44373 | -1.39325 | 2.86434 |
| H | 4.42327 | -1.87609 | 2.75125 |
| H | 3.13411 | -1.47473 | 3.91562 |
| H | 2.71238 | -1.96427 | 2.26962 |
| C | 1.23185 | 3.11331 | 2.13934 |
| H | 0.40856 | 2.74168 | 1.50730 |
| H | 0.84670 | 3.15765 | 3.16852 |
| H | 1.47746 | 4.13185 | 1.81206 |
| C | 5.94634 | 2.31922 | 0.55021 |
| H | 5.71559 | 2.64477 | -0.47593 |
| H | 6.38131 | 3.18563 | 1.07178 |
| H | 6.71607 | 1.53787 | 0.49373 |
| C | 5.94634 | -2.31922 | -0.55021 |
| H | 5.71559 | -2.64477 | 0.47593 |
| H | 6.38131 | -3.18563 | -1.07178 |
| H | 6.71607 | -1.53787 | -0.49373 |
| C | -5.94634 | 2.31922 | -0.55021 |
| H | -5.71559 | 2.64477 | 0.47593 |
| H | -6.38131 | 3.18563 | -1.07178 |
| H | -6.71607 | 1.53787 | -0.49373 |

```
C          -1.23185         3.11331        -2.13934
H          -0.84670         3.15765        -3.16852
H          -1.47746         4.13185        -1.81206
H          -0.40856         2.74168        -1.50730
C           1.23185        -3.11331        -2.13934
H           0.84670        -3.15765        -3.16852
H           1.47746        -4.13185        -1.81206
H           0.40856        -2.74168        -1.50730
C           3.44373         1.39325        -2.86434
H           4.42327         1.87609        -2.75125
H           3.13411         1.47473        -3.91562
H           2.71238         1.96427        -2.26962
C          -3.44373        -1.39325        -2.86434
H          -4.42327        -1.87609        -2.75125
H          -3.13411        -1.47473        -3.91562
H          -2.71238        -1.96427        -2.26962
```

### 3.2.3 [TerN]$_2$PAs (PBE-D3/def2-TZVP)

```
102
[TerN]2PAs PBE-D3/def2-TZVP
N          -0.00000         1.14754        -0.05911
N          -0.00000        -1.14754        -0.05911
P          -0.00000         0.00000        -1.33647
C           0.04899        -2.54336        -0.11266
C           0.47733        -3.21883        -1.28478
C          -0.32026        -3.30634         1.02534
C           0.50099        -4.61774        -1.30284
C          -0.27784        -4.70290         0.96586
C           0.12311        -5.36790        -0.19114
H           0.84102        -5.11447        -2.21393
H          -0.57403        -5.26686         1.85276
H           0.14882        -6.45778        -0.22338
C          -0.04899         2.54336        -0.11266
C          -0.47733         3.21883        -1.28478
C           0.32026         3.30634         1.02534
C          -0.50099         4.61774        -1.30284
C           0.27784         4.70290         0.96586
C          -0.12311         5.36790        -0.19114
H          -0.84102         5.11447        -2.21393
H           0.57403         5.26686         1.85276
H          -0.14882         6.45778        -0.22338
C           0.76014         2.65992         2.29440
C          -0.14462         2.53087         3.37106
C           2.09597         2.21253         2.43309
C           0.30026         1.94221         4.56104
C           2.49975         1.64721         3.64406
C           1.61304         1.49118         4.71618
H          -0.40766         1.81835         5.38443
H           3.53000         1.29464         3.74350
C          -0.95291         2.48360        -2.49015
C          -0.12303         2.35033        -3.62173
C          -2.26126         1.94615        -2.50192
C          -0.61699         1.68161        -4.74717
C          -2.71154         1.28468        -3.64575
C          -1.90295         1.13859        -4.77916
H           0.03203         1.56851        -5.61915
H          -3.72159         0.86512        -3.64923
```

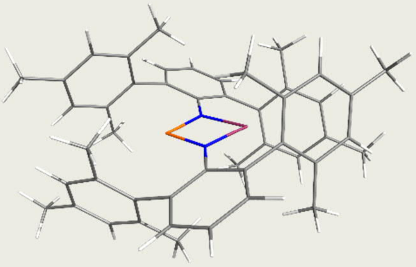

| | | | |
|---|---|---|---|
| C | -0.76014 | -2.65992 | 2.29440 |
| C | 0.14462 | -2.53087 | 3.37106 |
| C | -2.09597 | -2.21253 | 2.43309 |
| C | -0.30026 | -1.94221 | 4.56104 |
| C | -2.49975 | -1.64721 | 3.64406 |
| C | -1.61304 | -1.49118 | 4.71618 |
| H | 0.40766 | -1.81835 | 5.38443 |
| H | -3.53000 | -1.29464 | 3.74350 |
| C | 0.95291 | -2.48360 | -2.49015 |
| C | 2.26126 | -1.94615 | -2.50192 |
| C | 0.12303 | -2.35033 | -3.62173 |
| C | 2.71154 | -1.28468 | -3.64575 |
| C | 0.61699 | -1.68161 | -4.74717 |
| C | 1.90295 | -1.13859 | -4.77916 |
| H | 3.72159 | -0.86512 | -3.64923 |
| H | -0.03203 | -1.56851 | -5.61915 |
| C | 1.57395 | -2.98536 | 3.23997 |
| H | 2.16150 | -2.67582 | 4.11421 |
| H | 1.64461 | -4.07972 | 3.14795 |
| H | 2.04243 | -2.55906 | 2.34039 |
| C | -3.05526 | -2.31499 | 1.27988 |
| H | -3.09998 | -3.33900 | 0.88109 |
| H | -4.06567 | -2.00972 | 1.58063 |
| H | -2.73256 | -1.66366 | 0.45161 |
| C | -2.06775 | -0.84991 | 5.99942 |
| H | -2.51900 | 0.13626 | 5.81173 |
| H | -2.83165 | -1.46241 | 6.50313 |
| H | -1.23073 | -0.71505 | 6.69745 |
| C | -1.29782 | -2.84672 | -3.60534 |
| H | -1.75001 | -2.76882 | -4.60278 |
| H | -1.37010 | -3.89023 | -3.26947 |
| H | -1.90418 | -2.24344 | -2.90977 |
| C | 3.14148 | -2.04855 | -1.28625 |
| H | 2.73685 | -1.44118 | -0.45947 |
| H | 3.20566 | -3.08295 | -0.91809 |
| H | 4.15580 | -1.69044 | -1.50515 |
| C | 2.41058 | -0.41098 | -5.99479 |
| H | 2.69917 | 0.62211 | -5.74674 |
| H | 3.30296 | -0.90232 | -6.41210 |
| H | 1.64795 | -0.37034 | -6.78408 |
| C | -2.41058 | 0.41098 | -5.99479 |
| H | -2.69917 | -0.62211 | -5.74674 |
| H | -3.30296 | 0.90232 | -6.41210 |
| H | -1.64795 | 0.37034 | -6.78408 |
| C | 2.06775 | 0.84991 | 5.99942 |
| H | 2.51900 | -0.13626 | 5.81173 |
| H | 2.83165 | 1.46241 | 6.50313 |
| H | 1.23073 | 0.71505 | 6.69745 |
| C | 3.05526 | 2.31499 | 1.27988 |
| H | 3.09998 | 3.33900 | 0.88109 |
| H | 4.06567 | 2.00972 | 1.58063 |
| H | 2.73256 | 1.66366 | 0.45161 |
| C | -3.14148 | 2.04855 | -1.28625 |
| H | -3.20566 | 3.08295 | -0.91809 |
| H | -4.15580 | 1.69044 | -1.50515 |
| H | -2.73685 | 1.44118 | -0.45947 |
| C | 1.29782 | 2.84672 | -3.60534 |
| H | 1.75001 | 2.76882 | -4.60278 |
| H | 1.37010 | 3.89023 | -3.26947 |
| H | 1.90418 | 2.24344 | -2.90977 |

```
C           -1.57395         2.98536         3.23997
H           -2.16150         2.67582         4.11421
H           -1.64461         4.07972         3.14795
H           -2.04243         2.55906         2.34039
As           0.00000        -0.00000         1.48133
```

### 3.2.4 [TerNAs]$_2$ (PBE-D3/def2-TZVP)

```
102
[TerNAs]2 PBE-D3/def2-TZVP
N           -0.00000         0.00000        -1.18693
N           -0.00000         0.00000         1.18693
C            0.00000        -0.00000         2.58012
C            1.16158         0.37870         3.30716
C           -1.16158        -0.37870         3.30716
C            1.14105         0.36886         4.70537
C           -1.14105        -0.36886         4.70537
C            0.00000        -0.00000         5.41492
H            2.04641         0.67160         5.23582
H           -2.04641        -0.67160         5.23582
H            0.00000        -0.00000         6.50551
C            0.00000        -0.00000        -2.58012
C            1.16158        -0.37870        -3.30716
C           -1.16158         0.37870        -3.30716
C            1.14105        -0.36886        -4.70537
C           -1.14105         0.36886        -4.70537
C            0.00000        -0.00000        -5.41492
H            2.04641        -0.67160        -5.23582
H           -2.04641         0.67160        -5.23582
H            0.00000        -0.00000        -6.50551
C           -2.40913         0.81917        -2.62185
C           -3.50108        -0.06572        -2.49430
C           -2.50894         2.14349        -2.13265
C           -4.67014         0.38733        -1.87193
C           -3.69758         2.55480        -1.52621
C           -4.78749         1.68887        -1.37870
H           -5.50737        -0.30528        -1.75520
H           -3.76740         3.57617        -1.14184
C            2.40913        -0.81917        -2.62185
C            3.50108         0.06572        -2.49430
C            2.50894        -2.14349        -2.13265
C            4.67014        -0.38733        -1.87193
C            3.69758        -2.55480        -1.52621
C            4.78749        -1.68887        -1.37870
H            5.50737         0.30528        -1.75520
H            3.76740        -3.57617        -1.14184
C           -2.40913        -0.81917         2.62185
C           -3.50108         0.06572         2.49430
C           -2.50894        -2.14349         2.13265
C           -4.67014        -0.38733         1.87193
C           -3.69758        -2.55480         1.52621
C           -4.78749        -1.68887         1.37870
H           -5.50737         0.30528         1.75520
H           -3.76740        -3.57617         1.14184
C            2.40913         0.81917         2.62185
C            2.50894         2.14349         2.13265
```

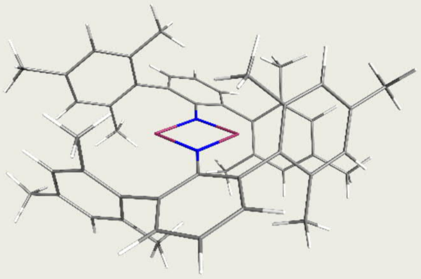

| | | | |
|---|---|---|---|
| C | 3.50108 | -0.06572 | 2.49430 |
| C | 3.69758 | 2.55480 | 1.52621 |
| C | 4.67014 | 0.38733 | 1.87193 |
| C | 4.78749 | 1.68887 | 1.37870 |
| H | 3.76740 | 3.57617 | 1.14184 |
| H | 5.50737 | -0.30528 | 1.75520 |
| C | -3.40354 | 1.48809 | 2.97787 |
| H | -4.30436 | 2.05395 | 2.70637 |
| H | -3.27895 | 1.54047 | 4.06965 |
| H | -2.53210 | 1.99375 | 2.53451 |
| C | -1.33933 | -3.08307 | 2.23654 |
| H | -0.97248 | -3.15711 | 3.27086 |
| H | -1.60999 | -4.08755 | 1.88659 |
| H | -0.49678 | -2.72171 | 1.62472 |
| C | -6.04993 | -2.15397 | 0.70432 |
| H | -5.83752 | -2.57154 | -0.29165 |
| H | -6.54471 | -2.94692 | 1.28648 |
| H | -6.76566 | -1.32982 | 0.58336 |
| C | 3.40354 | -1.48809 | 2.97787 |
| H | 4.30436 | -2.05395 | 2.70637 |
| H | 3.27895 | -1.54047 | 4.06965 |
| H | 2.53210 | -1.99375 | 2.53451 |
| C | 1.33933 | 3.08307 | 2.23654 |
| H | 0.49678 | 2.72171 | 1.62472 |
| H | 0.97248 | 3.15711 | 3.27086 |
| H | 1.60999 | 4.08755 | 1.88659 |
| C | 6.04993 | 2.15397 | 0.70432 |
| H | 5.83752 | 2.57154 | -0.29165 |
| H | 6.54471 | 2.94692 | 1.28648 |
| H | 6.76566 | 1.32982 | 0.58336 |
| C | 6.04993 | -2.15397 | -0.70432 |
| H | 5.83752 | -2.57154 | 0.29165 |
| H | 6.54471 | -2.94692 | -1.28648 |
| H | 6.76566 | -1.32982 | -0.58336 |
| C | -6.04993 | 2.15397 | -0.70432 |
| H | -5.83752 | 2.57154 | 0.29165 |
| H | -6.54471 | 2.94692 | -1.28648 |
| H | -6.76566 | 1.32982 | -0.58336 |
| C | -1.33933 | 3.08307 | -2.23654 |
| H | -0.97248 | 3.15711 | -3.27086 |
| H | -1.60999 | 4.08755 | -1.88659 |
| H | -0.49678 | 2.72171 | -1.62472 |
| C | 1.33933 | -3.08307 | -2.23654 |
| H | 0.97248 | -3.15711 | -3.27086 |
| H | 1.60999 | -4.08755 | -1.88659 |
| H | 0.49678 | -2.72171 | -1.62472 |
| C | 3.40354 | 1.48809 | -2.97787 |
| H | 4.30436 | 2.05395 | -2.70637 |
| H | 3.27895 | 1.54047 | -4.06965 |
| H | 2.53210 | 1.99375 | -2.53451 |
| C | -3.40354 | -1.48809 | -2.97787 |
| H | -4.30436 | -2.05395 | -2.70637 |
| H | -3.27895 | -1.54047 | -4.06965 |
| H | -2.53210 | -1.99375 | -2.53451 |
| As | -1.47290 | 0.00000 | -0.00000 |
| As | 1.47290 | -0.00000 | 0.00000 |

### 3.2.5 [TerNP]$_2$DmpNC (PBE-D3/def2-TZVP)

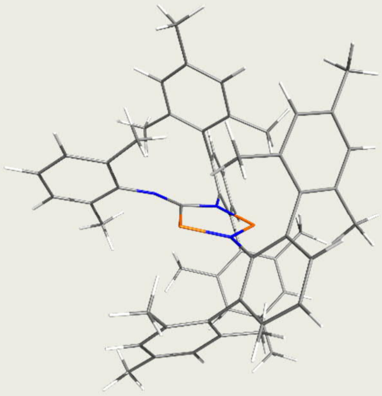

```
121
[TerNP]₂DmpNC PBE-D3/def2-TZVP
C         -5.92840       -0.25616        3.32277
C         -3.54586        0.43659        3.79905
C         -1.23923        1.21775        4.42406
C         -0.88276        4.70036        1.15456
C          4.13548        5.15978        0.96922
C         -4.44170       -0.45960        3.20671
C          1.62158        4.88268        0.99331
C         -2.16252        0.24177        3.74347
C          0.41608        4.43351        0.44007
C          2.84764        4.68211        0.35497
C         -3.91569       -1.55206        2.51297
C          0.21952       -1.78556        4.35526
C         -1.65491       -0.87894        3.04676
C          1.54980       -2.12845        4.56653
C         -2.53879       -1.77681        2.41342
C          2.84864        3.99990       -0.86639
C          0.44333        3.75002       -0.79317
C         -0.19998       -1.20409        3.14836
C         -1.49269        4.42593       -2.17433
C         -4.25915        1.16000        0.14539
C          1.67098        3.53195       -1.45597
C         -0.83991        3.39993       -1.47312
C         -2.67494        4.19213       -2.86958
C          2.48842       -1.85709        3.57550
C         -2.01685       -2.97470        1.67289
C          0.75773       -0.96733        2.13871
C          2.77770        1.56357        1.88521
C         -1.42071        2.11413       -1.47728
C         -3.23565        2.91721       -2.86074
C          2.12367       -1.26427        2.35953
C         -4.08142        0.19772       -0.99828
C         -2.63020        1.86431       -2.16450
C          1.71904        2.84569       -2.79376
C         -4.70301       -1.05517       -0.96069
C         -3.30695        0.53681       -2.12454
C          3.54674        0.45552        1.22128
C          3.22594       -0.90339        1.42535
C          0.53946       -1.13689       -0.33986
C         -4.57017       -1.97830       -2.00049
C          4.64614        0.78184        0.41585
C         -3.21414       -0.34855       -3.21890
C         -5.18202       -3.34983       -1.90172
C          4.04263       -1.91275        0.86258
C          3.77053       -3.37635        1.09005
C         -3.83553       -1.59732       -3.12843
C         -2.46751        0.03171       -4.46955
C          5.44724       -0.19661       -0.17359
C          5.13268       -1.53737        0.07344
C          6.59256        0.17491       -1.07597
H         -6.19387        0.80955        3.27140
H         -3.93371        1.31170        4.32786
H         -1.79939        2.08021        4.80816
H         -0.70081        5.16293        2.13324
H          3.95108        5.84879        1.80411
H          1.59691        5.40909        1.95129
```

| | | | |
|---|---|---|---|
| H | -6.30569 | -0.63812 | 4.28500 |
| H | -6.47009 | -0.78485 | 2.52617 |
| H | -0.70556 | 0.75243 | 5.26629 |
| H | 4.76521 | 5.67556 | 0.22971 |
| H | -1.53429 | 5.37103 | 0.57421 |
| H | -1.45457 | 3.77362 | 1.31694 |
| H | -0.46591 | 1.59124 | 3.73455 |
| H | 4.72660 | 4.31520 | 1.35991 |
| H | -0.53035 | -1.97337 | 5.12564 |
| H | 1.85792 | -2.58863 | 5.50659 |
| H | -4.59590 | -2.26077 | 2.03388 |
| H | -1.03876 | 5.41846 | -2.17495 |
| H | -5.15472 | 0.90991 | 0.72728 |
| H | -4.34849 | 2.19705 | -0.20665 |
| H | 3.79573 | 3.83287 | -1.38681 |
| H | -3.16026 | 5.00291 | -3.41501 |
| H | -3.40445 | 1.12078 | 0.83999 |
| H | -1.49738 | -3.66869 | 2.35210 |
| H | 2.32652 | 1.24065 | 2.83261 |
| H | 3.43127 | 2.42050 | 2.09103 |
| H | 3.54330 | -2.07908 | 3.74187 |
| H | -2.83499 | -3.51908 | 1.18351 |
| H | 1.96359 | 1.93803 | 1.24180 |
| H | 2.74597 | 2.81207 | -3.17932 |
| H | -5.30753 | -1.31354 | -0.08808 |
| H | -1.28269 | -2.69262 | 0.90606 |
| H | -4.17262 | 2.72019 | -3.38431 |
| H | 1.34772 | 1.81091 | -2.72587 |
| H | 1.08676 | 3.36315 | -3.53141 |
| H | -6.04213 | -3.35809 | -1.21791 |
| H | 4.28649 | -3.74506 | 1.99121 |
| H | 4.88594 | 1.83751 | 0.26069 |
| H | 2.69719 | -3.56230 | 1.21155 |
| H | -4.44827 | -4.07740 | -1.51784 |
| H | -2.98424 | 0.83591 | -5.01608 |
| H | -1.45598 | 0.39841 | -4.24172 |
| H | -5.51672 | -3.71508 | -2.88270 |
| H | 4.12870 | -3.96876 | 0.23755 |
| H | -3.73861 | -2.29432 | -3.96516 |
| H | -2.37263 | -0.82983 | -5.14285 |
| H | 5.74510 | -2.32244 | -0.37871 |
| H | 6.94141 | 1.19871 | -0.88240 |
| H | 7.44340 | -0.50979 | -0.95106 |
| H | 6.28865 | 0.12441 | -2.13455 |
| N | -0.75701 | 1.01985 | -0.80771 |
| N | 0.32868 | -0.41527 | 0.87784 |
| P | -0.53128 | 1.04982 | 0.85233 |
| P | -0.16085 | -0.29961 | -1.76818 |
| C | 2.33334 | -2.48581 | -2.44118 |
| C | 1.07187 | -4.37304 | -1.50212 |
| C | 2.71552 | -3.32120 | -3.50099 |
| C | 1.47848 | -5.16179 | -2.57722 |
| C | 2.29465 | -4.64403 | -3.58528 |
| H | 3.37781 | -2.91200 | -4.26863 |
| H | 1.15071 | -6.20353 | -2.61963 |
| H | 2.61219 | -5.27334 | -4.41817 |
| C | 1.47786 | -3.01052 | -1.43222 |
| N | 1.10436 | -2.30062 | -0.29181 |
| C | 0.19637 | -4.94598 | -0.42429 |
| H | 0.57353 | -4.68162 | 0.57466 |

```
H          -0.82705       -4.54364       -0.48213
H           0.13671       -6.03952       -0.50993
C           2.88150       -1.08587       -2.40370
H           2.26474       -0.39420       -3.00324
H           2.92324       -0.68003       -1.38698
H           3.89831       -1.06675       -2.82169
```

### 3.2.6 $[TerN]_2$PAsDmpNC (PBE-D3/def2-TZVP)

```
121
[TerN]2PAsDmpNC PBE-D3/def2-TZVP
As          0.25769       -0.40234       -1.84130
N          -0.40486       -0.43118        0.89203
N           0.86337        1.00598       -0.72313
N          -1.28732       -2.28099       -0.24725
C          -0.63083       -1.17213       -0.30848
P           0.49887        1.00636        0.89937
C          -0.88229       -0.94595        2.15627
C           1.56272        2.11075       -1.32288
C          -1.66840       -2.99025       -1.38584
C          -2.26517       -1.16750        2.36229
C           0.04942       -1.22813        3.17836
C           2.79786        1.87268       -1.97171
C           1.00219        3.40755       -1.30397
C          -1.33875       -4.37376       -1.42701
C          -2.46271       -2.42875       -2.42464
C          -2.67588       -1.73309        3.57648
C          -3.33673       -0.75501        1.41345
C          -0.41631       -1.77919        4.38329
C           1.52269       -0.98511        3.10205
C           3.44082        2.93634       -2.61525
C           3.46843        0.54206       -1.94345
C           1.69240        4.44441       -1.95196
C          -0.29658        3.77320       -0.66143
C          -1.75727       -5.15115       -2.50588
C          -0.53302       -4.98153       -0.31430
C          -2.85905       -3.25424       -3.48726
C          -2.93660       -1.00095       -2.41462
H          -3.74303       -1.89768        3.73070
C          -1.76507       -2.04947        4.57994
C          -3.58329        0.61735        1.19908
C          -4.19886       -1.72261        0.84507
H           0.31305       -2.00300        5.16362
C           2.07921        0.09258        3.82863
C           2.36888       -1.92504        2.47646
H           4.39559        2.74180       -3.10666
C           2.89594        4.21781       -2.61117
C           4.16568        0.15220       -0.78322
C           3.45505       -0.29291       -3.07980
H           1.24974        5.44202       -1.93918
C          -1.50433        3.61527       -1.37598
C          -0.29312        4.43686        0.58357
H          -1.48927       -6.21042       -2.52726
C          -2.51096       -4.59946       -3.54422
H          -0.54358       -6.07805       -0.37827
H          -0.91974       -4.67261        0.66798
```

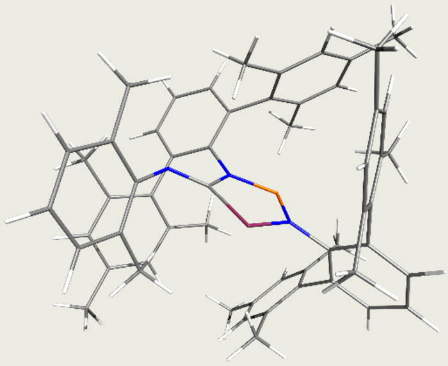

| | | | |
|---|---|---|---|
| H | 0.51585 | -4.64898 | -0.34966 |
| H | -3.47447 | -2.81845 | -4.27883 |
| H | -3.95064 | -0.93721 | -2.83511 |
| H | -2.28442 | -0.35232 | -3.02530 |
| H | -2.95777 | -0.57521 | -1.40559 |
| H | -2.10839 | -2.48812 | 5.51812 |
| C | -4.64921 | 0.99736 | 0.37230 |
| C | -2.77197 | 1.68676 | 1.87559 |
| C | -5.25263 | -1.29445 | 0.03383 |
| C | -4.01713 | -3.19714 | 1.09150 |
| C | 3.47024 | 0.20020 | 3.92557 |
| C | 1.20081 | 1.11535 | 4.49987 |
| C | 3.75324 | -1.78650 | 2.61586 |
| C | 1.79931 | -3.07523 | 1.69659 |
| H | 3.40960 | 5.03750 | -3.11570 |
| C | 4.79163 | -1.09839 | -0.76084 |
| C | 4.25871 | 1.06081 | 0.41264 |
| C | 4.07870 | -1.54196 | -3.00583 |
| C | 2.79251 | 0.13956 | -4.36149 |
| C | -2.68452 | 4.12674 | -0.82871 |
| C | -1.52647 | 2.94853 | -2.72410 |
| C | -1.49994 | 4.93190 | 1.09263 |
| C | 0.98466 | 4.63645 | 1.35560 |
| H | -2.83801 | -5.21985 | -4.38012 |
| H | -4.82991 | 2.06339 | 0.20840 |
| C | -5.48967 | 0.05949 | -0.22777 |
| H | -3.39477 | 2.56175 | 2.10044 |
| H | -1.95103 | 2.04516 | 1.23232 |
| H | -2.32633 | 1.33371 | 2.81455 |
| H | -5.89956 | -2.04855 | -0.42301 |
| H | -4.38504 | -3.77647 | 0.23412 |
| H | -4.57932 | -3.52639 | 1.98025 |
| H | -2.96065 | -3.44410 | 1.24573 |
| H | 3.89587 | 1.04207 | 4.47865 |
| C | 4.32579 | -0.74048 | 3.34420 |
| H | 1.80161 | 1.94675 | 4.89111 |
| H | 0.63455 | 0.67907 | 5.33640 |
| H | 0.45632 | 1.52825 | 3.80165 |
| H | 4.40140 | -2.52829 | 2.14221 |
| H | 2.59759 | -3.73589 | 1.33413 |
| H | 1.22385 | -2.72678 | 0.82797 |
| H | 1.10353 | -3.67021 | 2.30692 |
| H | 5.33575 | -1.39563 | 0.13887 |
| C | 4.73878 | -1.97173 | -1.85003 |
| H | 5.12521 | 0.79999 | 1.03250 |
| H | 3.36789 | 0.97142 | 1.05506 |
| H | 4.34526 | 2.11511 | 0.11590 |
| H | 4.04265 | -2.20036 | -3.87799 |
| H | 2.66150 | -0.71412 | -5.03896 |
| H | 3.39494 | 0.89777 | -4.88637 |
| H | 1.80504 | 0.58844 | -4.18214 |
| H | -3.61520 | 4.00824 | -1.39041 |
| C | -2.70531 | 4.79481 | 0.40015 |
| H | -2.53499 | 2.97342 | -3.15630 |
| H | -1.21187 | 1.89489 | -2.65458 |
| H | -0.83503 | 3.44124 | -3.42471 |
| H | -1.49272 | 5.44443 | 2.05843 |
| H | 0.78166 | 5.09154 | 2.33367 |
| H | 1.68837 | 5.28647 | 0.81404 |
| H | 1.50846 | 3.68294 | 1.52510 |

```
C          -6.59628        0.48557        -1.15406
C           5.81824       -0.63599         3.50739
C           5.35548       -3.34255        -1.77341
C          -3.99600        5.31869         0.96871
H          -7.48470       -0.15199        -1.04179
H          -6.27557        0.41219        -2.20635
H          -6.89356        1.52774        -0.97287
H           6.34930       -1.08345         2.65527
H           6.15412       -1.16524         4.41372
H           6.14040        0.41040         3.60337
H           5.71869       -3.67785        -2.75481
H           4.61635       -4.08422        -1.42905
H           6.19698       -3.36591        -1.06700
H          -3.81594        6.04371         1.77364
H          -4.60433        4.50127         1.39025
H          -4.60663        5.80605         0.19508
```

### 3.2.7 $[TerNP]_2{\cdot}H_2$ (PBE-D3/def2-TZVP)

```
104
[TerNP]2·H2 PBE-D3/def2-TZVP
N          -1.14969       -0.00124         0.12680
N           1.14969        0.00124         0.12680
P           0.00576        1.33922         0.02338
P          -0.00576       -1.33922         0.02338
C           2.47569        0.05670        -0.28643
C           3.16156        1.30077        -0.25177
C           3.17446       -1.10368        -0.70494
C           4.47596        1.37497        -0.72569
C           4.49307       -0.98333        -1.15796
C           5.14705        0.24724        -1.19362
H           4.98432        2.34076        -0.68546
H           5.00871       -1.89063        -1.48050
H           6.17581        0.31940        -1.54767
C          -2.47569       -0.05670        -0.28643
C          -3.17446        1.10368        -0.70494
C          -3.16156       -1.30077        -0.25177
C          -4.49307        0.98333        -1.15796
C          -4.47596       -1.37497        -0.72569
C          -5.14705       -0.24724        -1.19362
H          -5.00871        1.89063        -1.48050
H          -4.98432       -2.34076        -0.68546
H          -6.17581       -0.31940        -1.54767
C          -2.56259       -2.51395         0.37924
C          -2.24376       -3.66032        -0.38974
C          -2.44581       -2.56485         1.79079
C          -1.81756       -4.81929         0.26121
C          -2.00582       -3.74589         2.39699
C          -1.68898       -4.88506         1.65332
H          -1.56511       -5.69625        -0.33957
H          -1.92269       -3.77724         3.48701
C          -2.57764        2.47103        -0.65151
C          -2.61433        3.20324         0.55660
C          -2.08367        3.07824        -1.82926
C          -2.15915        4.52694         0.56220
C          -1.64847        4.40275        -1.77929
```

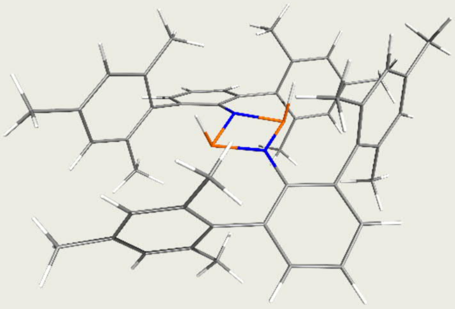

| | | | |
|---|---|---|---|
| C | -1.68898 | 5.15034 | -0.59640 |
| H | -2.19185 | 5.09083 | 1.49865 |
| H | -1.25866 | 4.86529 | -2.69038 |
| C | 2.57764 | -2.47103 | -0.65151 |
| C | 2.61433 | -3.20324 | 0.55660 |
| C | 2.08367 | -3.07824 | -1.82926 |
| C | 2.15915 | -4.52694 | 0.56220 |
| C | 1.64847 | -4.40275 | -1.77929 |
| C | 1.68898 | -5.15034 | -0.59640 |
| H | 2.19185 | -5.09083 | 1.49865 |
| H | 1.25866 | -4.86529 | -2.69038 |
| C | 2.56259 | 2.51395 | 0.37924 |
| C | 2.44581 | 2.56485 | 1.79079 |
| C | 2.24376 | 3.66032 | -0.38974 |
| C | 2.00582 | 3.74589 | 2.39699 |
| C | 1.81756 | 4.81929 | 0.26121 |
| C | 1.68898 | 4.88506 | 1.65332 |
| H | 1.92269 | 3.77724 | 3.48701 |
| H | 1.56511 | 5.69625 | -0.33957 |
| C | 3.15384 | -2.58041 | 1.81680 |
| H | 3.14114 | -3.30001 | 2.64561 |
| H | 4.18637 | -2.22742 | 1.67497 |
| H | 2.56416 | -1.70191 | 2.11842 |
| C | 1.96043 | -2.28539 | -3.09967 |
| H | 2.91060 | -1.81301 | -3.38564 |
| H | 1.61669 | -2.91774 | -3.92849 |
| H | 1.23078 | -1.46846 | -2.96763 |
| C | 1.28171 | -6.59855 | -0.58945 |
| H | 0.49406 | -6.79849 | -1.32952 |
| H | 2.13708 | -7.24535 | -0.84369 |
| H | 0.91666 | -6.91158 | 0.39759 |
| C | 2.27500 | 3.61258 | -1.89125 |
| H | 2.02169 | 4.59116 | -2.31930 |
| H | 3.25213 | 3.29935 | -2.28314 |
| H | 1.53586 | 2.87743 | -2.25229 |
| C | 2.79806 | 1.37520 | 2.64065 |
| H | 2.06151 | 0.56678 | 2.50639 |
| H | 3.77691 | 0.95832 | 2.36254 |
| H | 2.81794 | 1.64592 | 3.70411 |
| C | 1.19920 | 6.13450 | 2.33334 |
| H | 0.11475 | 6.07759 | 2.52351 |
| H | 1.69022 | 6.28083 | 3.30579 |
| H | 1.38015 | 7.02617 | 1.71784 |
| C | -1.28171 | 6.59855 | -0.58945 |
| H | -0.49406 | 6.79849 | -1.32952 |
| H | -2.13708 | 7.24535 | -0.84369 |
| H | -0.91666 | 6.91158 | 0.39759 |
| C | -1.19920 | -6.13450 | 2.33334 |
| H | -0.11475 | -6.07759 | 2.52351 |
| H | -1.69022 | -6.28083 | 3.30579 |
| H | -1.38015 | -7.02617 | 1.71784 |
| C | -2.79806 | -1.37520 | 2.64065 |
| H | -3.77691 | -0.95832 | 2.36254 |
| H | -2.81794 | -1.64592 | 3.70411 |
| H | -2.06151 | -0.56678 | 2.50639 |
| C | -1.96043 | 2.28539 | -3.09967 |
| H | -2.91060 | 1.81301 | -3.38564 |
| H | -1.61669 | 2.91774 | -3.92849 |
| H | -1.23078 | 1.46846 | -2.96763 |
| C | -3.15384 | 2.58041 | 1.81680 |

```
H          -3.14114         3.30001         2.64561
H          -4.18637         2.22742         1.67497
H          -2.56416         1.70191         2.11842
C          -2.27500        -3.61258        -1.89125
H          -2.02169        -4.59116        -2.31930
H          -3.25213        -3.29935        -2.28314
H          -1.53586        -2.87743        -2.25229
H           0.06365        -1.75686         1.40766
H          -0.06365         1.75686         1.40766
```

### 3.2.8 [TerN]$_2$PAs·H2 (PBE-D3/def2-TZVP)

```
104
[TerN]2PAs·H2 PBE-D3/def2-TZVP
N           0.07959         1.13385         0.18944
N          -0.08285        -1.18264        -0.20918
P          -1.29869         0.00090         0.14280
C          -0.19557        -2.56042        -0.06482
C          -1.42840        -3.20050        -0.35907
C           0.89668        -3.34199         0.39269
C          -1.54115        -4.58608        -0.19483
C           0.74928        -4.72784         0.51352
C          -0.46090        -5.35865         0.22770
H          -2.49624        -5.05903        -0.43362
H           1.60156        -5.30941         0.87172
H          -0.56140        -6.43916         0.33619
C           0.00643         2.51606         0.05460
C          -1.19048         3.21995         0.36707
C           1.13598         3.26992        -0.36869
C          -1.23727         4.61111         0.22166
C           1.04497         4.65877        -0.49955
C          -0.13501         5.34264        -0.21436
H          -2.17033         5.11930         0.47480
H           1.93328         5.20442        -0.82548
H          -0.18993         6.42680        -0.31569
C           2.44113         2.61505        -0.66445
C           3.30742         2.25630         0.39854
C           2.85378         2.42104        -2.00461
C           4.55070         1.69590         0.10229
C           4.09529         1.82460        -2.25150
C           4.95574         1.45192        -1.21424
H           5.21468         1.42317         0.92509
H           4.40083         1.65263        -3.28717
C          -2.39471         2.52649         0.90760
C          -3.54567         2.33506         0.11217
C          -2.40308         2.11967         2.26611
C          -4.66742         1.71829         0.68089
C          -3.54428         1.51370         2.78917
C          -4.68731         1.29494         2.00958
H          -5.55171         1.56006         0.05808
H          -3.54148         1.19877         3.83656
C           2.18459        -2.71478         0.79949
C           3.33054        -2.85318        -0.01223
C           2.26632        -2.00362         2.01768
C           4.54022        -2.29530         0.41453
C           3.49490        -1.45755         2.40252
```

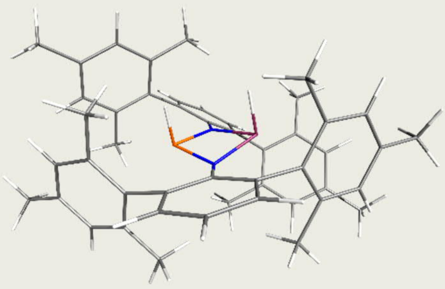

| | | | |
|---|---|---|---|
| C | 4.64729 | -1.60219 | 1.62346 |
| H | 5.42537 | -2.40805 | -0.21818 |
| H | 3.55467 | -0.91126 | 3.34820 |
| C | -2.59882 | -2.43546 | -0.88274 |
| C | -2.60176 | -2.00484 | -2.23043 |
| C | -3.73378 | -2.20663 | -0.07157 |
| C | -3.72739 | -1.34570 | -2.73364 |
| C | -4.84018 | -1.55079 | -0.62020 |
| C | -4.85732 | -1.10558 | -1.94479 |
| H | -3.72231 | -1.01725 | -3.77692 |
| H | -5.71107 | -1.36995 | 0.01522 |
| C | 3.25878 | -3.57009 | -1.33573 |
| H | 4.16176 | -3.38067 | -1.93113 |
| H | 3.16181 | -4.65853 | -1.20438 |
| H | 2.38176 | -3.25221 | -1.91886 |
| C | 1.05725 | -1.82564 | 2.89607 |
| H | 0.52530 | -2.77632 | 3.04410 |
| H | 1.34235 | -1.42534 | 3.87778 |
| H | 0.33464 | -1.12929 | 2.44150 |
| C | 5.97205 | -1.06653 | 2.09576 |
| H | 5.84341 | -0.19240 | 2.74966 |
| H | 6.51808 | -1.82785 | 2.67602 |
| H | 6.61524 | -0.77793 | 1.25286 |
| C | -3.73664 | -2.57498 | 1.38640 |
| H | -4.75340 | -2.53253 | 1.79872 |
| H | -3.32411 | -3.57543 | 1.56867 |
| H | -3.10999 | -1.85990 | 1.94684 |
| C | -1.40798 | -2.23834 | -3.11494 |
| H | -0.57634 | -1.57495 | -2.82664 |
| H | -1.03770 | -3.26999 | -3.02743 |
| H | -1.65234 | -2.03770 | -4.16619 |
| C | -6.04315 | -0.36256 | -2.49831 |
| H | -5.86560 | 0.72539 | -2.48744 |
| H | -6.24572 | -0.64553 | -3.54115 |
| H | -6.94878 | -0.55506 | -1.90699 |
| C | -5.89675 | 0.61634 | 2.59302 |
| H | -5.67216 | -0.42850 | 2.85856 |
| H | -6.23280 | 1.11691 | 3.51352 |
| H | -6.73437 | 0.61099 | 1.88274 |
| C | 6.26867 | 0.77784 | -1.50348 |
| H | 6.16227 | -0.31687 | -1.43375 |
| H | 6.62838 | 1.01115 | -2.51472 |
| H | 7.04218 | 1.07720 | -0.78236 |
| C | 1.97828 | 2.84207 | -3.15442 |
| H | 1.96564 | 3.93757 | -3.26297 |
| H | 2.33898 | 2.41106 | -4.09751 |
| H | 0.93514 | 2.53179 | -3.00217 |
| C | -1.18642 | 2.30776 | 3.12963 |
| H | -0.79784 | 3.33423 | 3.06395 |
| H | -1.41198 | 2.08153 | 4.17978 |
| H | -0.37292 | 1.63992 | 2.80232 |
| C | -3.58152 | 2.70506 | -1.34705 |
| H | -4.59001 | 3.02667 | -1.64313 |
| H | -2.87025 | 3.50212 | -1.59269 |
| H | -3.31922 | 1.83029 | -1.96590 |
| C | 2.88211 | 2.44392 | 1.82797 |
| H | 3.71683 | 2.25095 | 2.51394 |
| H | 2.50035 | 3.45927 | 2.00625 |
| H | 2.06542 | 1.74904 | 2.08250 |
| As | 1.38728 | -0.02990 | -0.61644 |

```
H          -1.70654        0.36239       -1.20708
H           0.86678        0.23147       -2.08430
```

### 3.2.9 [TerNAs]$_2$·H2 (PBE-D3/def2-TZVP)

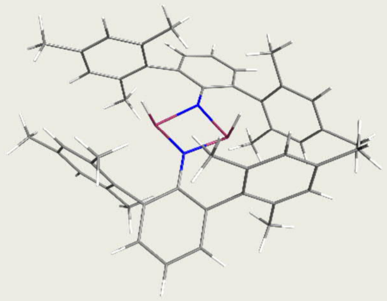

```
104
[TerNAs]₂·H2 PBE-D3/def2-TZVP
N          -1.22941       -0.04208        0.15990
N           1.22941        0.04208        0.15990
C           2.54493        0.14830       -0.25427
C           3.20500        1.41261       -0.20585
C           3.29597       -0.98161       -0.68583
C           4.52118        1.52974       -0.66489
C           4.61426       -0.81783       -1.12291
C           5.23459        0.43028       -1.13701
H           4.99920        2.51037       -0.60891
H           5.15903       -1.70617       -1.45122
H           6.26483        0.53747       -1.47711
C          -2.54493       -0.14830       -0.25427
C          -3.29597        0.98161       -0.68583
C          -3.20500       -1.41261       -0.20585
C          -4.61426        0.81783       -1.12291
C          -4.52118       -1.52974       -0.66489
C          -5.23459       -0.43028       -1.13701
H          -5.15903        1.70617       -1.45122
H          -4.99920       -2.51037       -0.60891
H          -6.26483       -0.53747       -1.47711
C          -2.57253       -2.61263        0.41750
C          -2.25084       -3.75484       -0.35784
C          -2.41633       -2.66053        1.82592
C          -1.77596       -4.90220        0.28172
C          -1.93679       -3.83168        2.42096
C          -1.60947       -4.96326        1.66969
H          -1.51792       -5.77347       -0.32521
H          -1.82314       -3.85914        3.50832
C          -2.73728        2.36452       -0.66794
C          -2.72500        3.10424        0.53733
C          -2.30361        2.97107       -1.87087
C          -2.26866        4.42792        0.51751
C          -1.86600        4.29566       -1.84535
C          -1.84541        5.04493       -0.66250
H          -2.25587        4.99455        1.45268
H          -1.51738        4.75520       -2.77444
C           2.73728       -2.36452       -0.66794
C           2.72500       -3.10424        0.53733
C           2.30361       -2.97107       -1.87087
C           2.26866       -4.42792        0.51751
C           1.86600       -4.29566       -1.84535
C           1.84541       -5.04493       -0.66250
H           2.25587       -4.99455        1.45268
H           1.51738       -4.75520       -2.77444
C           2.57253        2.61263        0.41750
C           2.41633        2.66053        1.82592
C           2.25084        3.75484       -0.35784
C           1.93679        3.83168        2.42096
C           1.77596        4.90220        0.28172
```

| | | | |
|---|---|---|---|
| C | 1.60947 | 4.96326 | 1.66969 |
| H | 1.82314 | 3.85914 | 3.50832 |
| H | 1.51792 | 5.77347 | -0.32521 |
| C | 3.21069 | -2.48496 | 1.82040 |
| H | 3.14517 | -3.20024 | 2.65040 |
| H | 4.25478 | -2.15037 | 1.72623 |
| H | 2.62151 | -1.59471 | 2.08608 |
| C | 2.25084 | -2.17733 | -3.14665 |
| H | 3.23967 | -1.78931 | -3.42984 |
| H | 1.86287 | -2.78642 | -3.97332 |
| H | 1.59621 | -1.29837 | -3.02829 |
| C | 1.42028 | -6.48790 | -0.67618 |
| H | 0.63640 | -6.66829 | -1.42524 |
| H | 2.26946 | -7.14240 | -0.93125 |
| H | 1.04246 | -6.80688 | 0.30400 |
| C | 2.35240 | 3.72330 | -1.85792 |
| H | 1.96903 | 4.65544 | -2.29316 |
| H | 3.38559 | 3.57761 | -2.20336 |
| H | 1.76407 | 2.88483 | -2.26430 |
| C | 2.76254 | 1.47423 | 2.68126 |
| H | 2.04004 | 0.65809 | 2.51840 |
| H | 3.75392 | 1.07237 | 2.42627 |
| H | 2.74984 | 1.74039 | 3.74595 |
| C | 1.06897 | 6.19889 | 2.33665 |
| H | -0.01128 | 6.09866 | 2.53271 |
| H | 1.55652 | 6.37775 | 3.30551 |
| H | 1.21040 | 7.08997 | 1.71010 |
| C | -1.42028 | 6.48790 | -0.67618 |
| H | -0.63640 | 6.66829 | -1.42524 |
| H | -2.26946 | 7.14240 | -0.93125 |
| H | -1.04246 | 6.80688 | 0.30400 |
| C | -1.06897 | -6.19889 | 2.33665 |
| H | 0.01128 | -6.09866 | 2.53271 |
| H | -1.55652 | -6.37775 | 3.30551 |
| H | -1.21040 | -7.08997 | 1.71010 |
| C | -2.76254 | -1.47423 | 2.68126 |
| H | -3.75392 | -1.07237 | 2.42627 |
| H | -2.74984 | -1.74039 | 3.74595 |
| H | -2.04004 | -0.65809 | 2.51840 |
| C | -2.25084 | 2.17733 | -3.14665 |
| H | -3.23967 | 1.78931 | -3.42984 |
| H | -1.86287 | 2.78642 | -3.97332 |
| H | -1.59621 | 1.29837 | -3.02829 |
| C | -3.21069 | 2.48496 | 1.82040 |
| H | -3.14517 | 3.20024 | 2.65040 |
| H | -4.25478 | 2.15037 | 1.72623 |
| H | -2.62151 | 1.59471 | 2.08608 |
| C | -2.35240 | -3.72330 | -1.85792 |
| H | -1.96903 | -4.65544 | -2.29316 |
| H | -3.38559 | -3.57761 | -2.20336 |
| H | -1.76407 | -2.88483 | -2.26430 |
| As | 0.04414 | -1.46521 | -0.01515 |
| As | -0.04414 | 1.46521 | -0.01515 |
| H | 0.14320 | -1.87359 | 1.48203 |
| H | -0.14320 | 1.87359 | 1.48203 |

### 3.2.10 $[TerNP]_2DmpNC\cdot H2$ (PBE-D3/def2-TZVP)

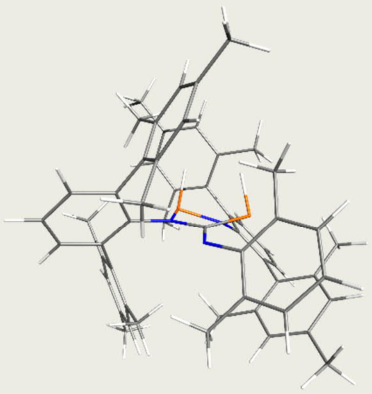

```
123
[TerNP]2DmpNC·H2 PBE-D3/def2-TZVP
C          -4.67462       -1.90901        4.23467
C          -3.91369        0.30479        3.28097
C          -3.32887        2.52744        2.26985
C          -5.04213        0.14300       -0.15861
C          -4.18238        4.41299       -2.69269
C          -3.59575       -0.93597        3.84207
C          -4.60127        2.22110       -1.50492
C          -2.92830        1.21013        2.87535
C          -4.37020        0.85787       -1.29700
C          -3.96795        2.93261       -2.52993
C          -2.24505       -1.25452        4.00161
C          -0.33809        2.85146        3.73016
C          -1.56941        0.85307        3.02143
C           0.62913        3.84200        3.61116
C          -1.22585       -0.38629        3.59644
C          -3.11048        2.23255       -3.38428
C          -3.45745        0.18096       -2.13806
C          -0.50183        1.86185        2.74822
C          -4.12803       -2.21417       -2.34812
C          -1.19182       -3.21245        1.48812
C          -2.84594        0.86988       -3.21136
C          -3.17225       -1.27181       -1.94783
C          -3.86230       -3.57961       -2.28729
C           1.42872        3.86846        2.47304
C           0.21619       -0.76046        3.79115
C           0.33769        1.87601        1.60885
C           0.10129        4.03573       -1.02726
C          -1.92961       -1.71725       -1.43573
C          -2.62217       -4.00963       -1.81930
C           1.29161        2.91592        1.45519
C          -0.13659       -3.68579        0.53074
C          -1.64678       -3.10427       -1.38284
C          -1.92856        0.15396       -4.16516
C           1.07848       -4.18943        1.01015
C          -0.35656       -3.63882       -0.85909
C           1.55916        3.65788       -0.93492
C           2.13251        3.12990        0.24025
C           1.23670        0.11107        0.09939
C           2.07579       -4.65558        0.15073
C           2.38919        3.94555       -2.02814
C           0.62596       -4.13074       -1.74693
C           3.37735       -5.19244        0.68024
C           3.53607        2.96338        0.32320
C           4.19832        2.47553        1.58453
C           1.82369       -4.62344       -1.22614
C           0.42435       -4.05793       -3.23551
C           3.77141        3.76362       -1.97681
C           4.32448        3.28447       -0.78408
C           4.63946        4.04170       -3.17408
H          -4.94641       -2.55619        3.38479
H          -4.96434        0.58196        3.15853
H          -4.41711        2.66469        2.31762
H          -5.69146        0.82712        0.40261
H          -5.16826        4.72097       -2.31840
H          -5.28866        2.74572       -0.83579
```

| | | | |
|---|---|---|---|
| H | -5.58888 | -1.38964 | 4.55426 |
| H | -4.34594 | -2.56540 | 5.05227 |
| H | -2.84770 | 3.37804 | 2.77250 |
| H | -4.09992 | 4.71845 | -3.74499 |
| H | -5.65025 | -0.70382 | -0.50733 |
| H | -4.29452 | -0.26638 | 0.53869 |
| H | -3.02866 | 2.57100 | 1.20932 |
| H | -3.42509 | 4.98067 | -2.12756 |
| H | -0.98730 | 2.81475 | 4.60701 |
| H | 0.74886 | 4.59629 | 4.39021 |
| H | -1.97198 | -2.21019 | 4.45707 |
| H | -5.07980 | -1.85395 | -2.74369 |
| H | -0.90175 | -3.42304 | 2.52441 |
| H | -2.15991 | -3.69779 | 1.29342 |
| H | -2.63052 | 2.76131 | -4.21249 |
| H | -4.61109 | -4.30334 | -2.61178 |
| H | -1.36179 | -2.12934 | 1.40396 |
| H | 0.73704 | -0.03168 | 4.43108 |
| H | -0.47863 | 3.69553 | -0.16226 |
| H | 0.00225 | 5.13212 | -1.07537 |
| H | 2.16750 | 4.66022 | 2.33808 |
| H | 0.30872 | -1.75175 | 4.25372 |
| H | -0.36551 | 3.62813 | -1.93588 |
| H | -1.68519 | 0.79036 | -5.02568 |
| H | 1.24472 | -4.22280 | 2.09058 |
| H | 0.75853 | -0.77282 | 2.83552 |
| H | -2.39463 | -5.07603 | -1.76299 |
| H | -0.98220 | -0.12808 | -3.67890 |
| H | -2.38194 | -0.77818 | -4.53216 |
| H | 3.46953 | -5.02021 | 1.76096 |
| H | 4.37673 | 3.30530 | 2.28706 |
| H | 1.93627 | 4.35014 | -2.93817 |
| H | 3.57350 | 1.73279 | 2.09454 |
| H | 4.23171 | -4.70694 | 0.18748 |
| H | -0.47604 | -4.59908 | -3.55982 |
| H | 0.29478 | -3.01088 | -3.55704 |
| H | 3.46161 | -6.27606 | 0.50144 |
| H | 5.16940 | 2.01611 | 1.35689 |
| H | 2.59340 | -4.97648 | -1.91793 |
| H | 1.28985 | -4.47232 | -3.76843 |
| H | 5.40618 | 3.13869 | -0.71640 |
| H | 4.16510 | 4.75916 | -3.85768 |
| H | 5.61866 | 4.44259 | -2.87647 |
| H | 4.82737 | 3.11808 | -3.74618 |
| N | -0.97078 | -0.76872 | -0.99188 |
| N | 0.18017 | 0.87969 | 0.58578 |
| P | -1.39427 | 0.71773 | -0.21098 |
| P | 0.71476 | -0.83789 | -1.43339 |
| C | 4.07236 | -0.56557 | -1.00162 |
| C | 4.12756 | -1.46258 | 1.28265 |
| C | 5.25425 | -1.27391 | -1.26500 |
| C | 5.30008 | -2.14746 | 0.97105 |
| C | 5.86796 | -2.06570 | -0.30183 |
| H | 5.70394 | -1.17830 | -2.25657 |
| H | 5.77657 | -2.75294 | 1.74568 |
| H | 6.79222 | -2.59847 | -0.53099 |
| C | 3.48407 | -0.67605 | 0.28531 |
| N | 2.36376 | 0.04206 | 0.70560 |
| C | 3.53128 | -1.56703 | 2.65522 |
| H | 3.25976 | -0.57558 | 3.04607 |

```
H          2.60291        -2.15835         2.63936
H          4.23207        -2.04948         3.34971
C          3.51746         0.30532        -2.09515
H          2.93082        -0.28225        -2.82179
H          2.88095         1.11108        -1.71285
H          4.33762         0.77226        -2.65803
H          0.71105         0.38687        -2.20695
H         -1.07511         1.59466        -1.30765
```

### 3.2.11 [TerN]$_2$PAsDmpNC·H2 (PBE-D3/def2-TZVP)

```
123
[TerN]2PAsDmpNC·H2 PBE-D3/def2-TZVP
As        -0.76488        -1.06593        -1.33402
N         -0.22689         0.93144         0.59456
N          1.06016        -0.80966        -0.83874
N         -2.39992         0.09004         0.84149
C         -1.28456         0.11920         0.21376
P          1.33687         0.77781        -0.23670
C         -0.37743         2.00306         1.54675
C          2.10408        -1.70348        -1.16515
C         -3.52147        -0.66589         0.49822
C         -1.32415         3.03443         1.31677
C          0.45724         2.06285         2.68750
C          1.92677        -3.10596        -1.03014
C          3.34270        -1.20656        -1.64927
C         -4.14259        -1.38443         1.55957
C         -4.13134        -0.65605        -0.78412
C         -1.46204         4.05602         2.26518
C         -2.15567         3.16181         0.08399
C          0.29053         3.11623         3.60033
C          1.52531         1.07550         3.02665
C          3.00102        -3.96003        -1.30340
C          0.61319        -3.69556        -0.64651
C          4.39534        -2.09838        -1.89288
C          3.52874         0.23502        -1.98952
C         -5.31380        -2.09968         1.31785
C         -3.52800        -1.38222         2.92789
C         -5.31195        -1.39018        -0.97550
C         -3.60354         0.13319        -1.95151
H         -2.19536         4.84068         2.07166
C         -0.67064         4.10124         3.40833
C         -1.56999         3.60033        -1.12188
C         -3.55960         3.00218         0.16597
H          0.93461         3.13650         4.48149
C          2.88355         1.42639         2.86260
C          1.18317        -0.12542         3.67843
H          2.84824        -5.03389        -1.17763
C          4.23987        -3.46973        -1.71182
C          0.16171        -3.62965         0.68780
C         -0.18863        -4.31258        -1.63200
H          5.33879        -1.69688        -2.26799
C          2.88539         0.76597        -3.13201
C          4.39955         1.05386        -1.23273
H         -5.77214        -2.65145         2.14197
C         -5.90434        -2.11328         0.05235
```

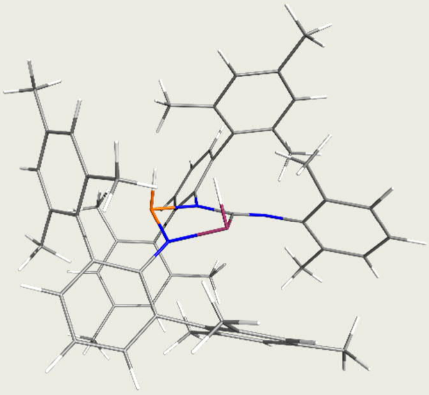

| | | | |
|---|---|---|---|
| H | -4.21372 | -1.82171 | 3.66443 |
| H | -3.26502 | -0.36190 | 3.24249 |
| H | -2.59181 | -1.96088 | 2.94480 |
| H | -5.77766 | -1.37074 | -1.96406 |
| H | -4.43685 | 0.57698 | -2.51391 |
| H | -3.05820 | -0.51031 | -2.66411 |
| H | -2.93939 | 0.94976 | -1.64854 |
| H | -0.79125 | 4.90670 | 4.13427 |
| C | -2.38742 | 3.79413 | -2.24446 |
| C | -0.11616 | 3.99204 | -1.21933 |
| C | -4.33645 | 3.23507 | -0.97154 |
| C | -4.23461 | 2.61455 | 1.45521 |
| C | 3.86911 | 0.54529 | 3.31938 |
| C | 3.28221 | 2.71310 | 2.19344 |
| C | 2.20173 | -0.97396 | 4.12420 |
| C | -0.25832 | -0.47151 | 3.91870 |
| H | 5.06708 | -4.15210 | -1.91097 |
| C | -1.10795 | -4.12545 | 1.00113 |
| C | 1.03146 | -3.03564 | 1.75701 |
| C | -1.44616 | -4.80489 | -1.27261 |
| C | 0.25657 | -4.37212 | -3.06919 |
| C | 3.06511 | 2.11743 | -3.44897 |
| C | 2.02656 | -0.10157 | -4.01138 |
| C | 4.54617 | 2.39789 | -1.58400 |
| C | 5.11785 | 0.50468 | -0.03299 |
| H | -6.82635 | -2.66976 | -0.12253 |
| H | -1.92493 | 4.12486 | -3.17911 |
| C | -3.77021 | 3.61348 | -2.19368 |
| H | -0.02981 | 5.09040 | -1.25542 |
| H | 0.34853 | 3.59863 | -2.13471 |
| H | 0.47463 | 3.65269 | -0.36132 |
| H | -5.41907 | 3.09602 | -0.90352 |
| H | -5.20737 | 2.14582 | 1.25524 |
| H | -4.41227 | 3.49589 | 2.09201 |
| H | -3.61876 | 1.90839 | 2.02495 |
| H | 4.91962 | 0.81776 | 3.18663 |
| C | 3.55210 | -0.66544 | 3.94297 |
| H | 4.36918 | 2.85834 | 2.24231 |
| H | 2.79401 | 3.58492 | 2.65083 |
| H | 2.98724 | 2.70135 | 1.13089 |
| H | 1.92864 | -1.90343 | 4.63111 |
| H | -0.35440 | -1.44414 | 4.41854 |
| H | -0.82440 | -0.50576 | 2.97810 |
| H | -0.75109 | 0.28701 | 4.54665 |
| H | -1.45778 | -4.06215 | 2.03536 |
| C | -1.93708 | -4.70143 | 0.03316 |
| H | 0.57638 | -3.16181 | 2.74685 |
| H | 1.19328 | -1.96042 | 1.59600 |
| H | 2.02776 | -3.50201 | 1.77004 |
| H | -2.07416 | -5.26017 | -2.04325 |
| H | -0.52761 | -4.80819 | -3.70148 |
| H | 1.17246 | -4.96731 | -3.19539 |
| H | 0.48787 | -3.36531 | -3.45343 |
| H | 2.55928 | 2.52287 | -4.32976 |
| C | 3.87143 | 2.95692 | -2.67599 |
| H | 1.80329 | 0.40412 | -4.95975 |
| H | 1.06700 | -0.33864 | -3.52656 |
| H | 2.51758 | -1.06097 | -4.22870 |
| H | 5.19821 | 3.03196 | -0.97699 |
| H | 5.66015 | 1.29984 | 0.49438 |

```
H           5.84026        -0.27691        -0.30949
H           4.40766         0.04508         0.67099
C          -4.62447         3.78815        -3.42012
C           4.63203        -1.61740         4.38150
C          -3.32415        -5.16737         0.37881
C           3.99274         4.42277        -2.99305
H          -5.61223         4.19855        -3.16721
H          -4.79342         2.82108        -3.92215
H          -4.14875         4.45671        -4.15075
H           4.90643        -2.30176         3.56212
H           4.30333        -2.23639         5.22774
H           5.54488        -1.08216         4.67820
H          -3.65204        -5.97990        -0.28427
H          -4.04403        -4.33990         0.27625
H          -3.38160        -5.52169         1.41762
H           4.97976         4.81583        -2.71242
H           3.23932         5.00538        -2.43789
H           3.83328         4.61765        -4.06254
H          -0.73522         0.20041        -2.24455
H           0.91961         1.50051        -1.40989
```

### 3.2.12 [TerNP]$_2$·$H_2$_TS (PBE-D3/def2-TZVP)

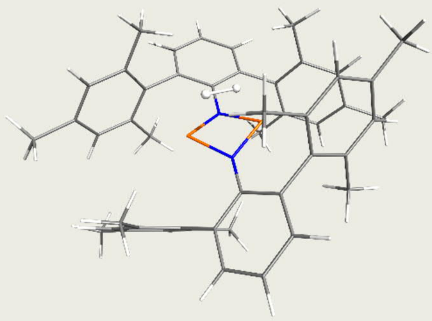

```
104
[TerNP]₂·H₂_TS PBE-D3/def2-TZVP
N          -0.07048         1.12641        -0.09252
N           0.03200        -1.12105         0.08266
P           1.31596         0.04000         0.04657
P          -1.23141        -0.01529         0.54145
C           0.05692        -2.52127         0.05869
C           1.21792        -3.23081         0.44996
C          -1.07845        -3.23444        -0.40073
C           1.21410        -4.62955         0.40530
C          -1.04289        -4.63385        -0.41673
C           0.09053        -5.33803        -0.01583
H           2.11738        -5.15883         0.71562
H          -1.92331        -5.16772        -0.78013
H           0.10238        -6.42835        -0.04306
C          -0.07107         2.52709        -0.00475
C           1.06773         3.26271        -0.42185
C          -1.20887         3.23131         0.46095
C           1.05278         4.65941        -0.35067
C          -1.18348         4.62943         0.51440
C          -0.06243         5.35292         0.11464
H           1.94120         5.20065        -0.68270
H          -2.07457         5.14618         0.87688
H          -0.06044         6.44262         0.15793
C          -2.44455         2.52046         0.89181
C          -3.41980         2.16118        -0.06467
C          -2.66041         2.24893         2.26310
C          -4.58287         1.51613         0.36425
C          -3.82610         1.57793         2.64190
C          -4.79495         1.19506         1.70709
H          -5.33423         1.23651        -0.37726
H          -3.98309         1.35032         3.69990
C           2.28107         2.58130        -0.95160
```

| | | | |
|---|---|---|---|
| C | 3.42333 | 2.43202 | -0.13671 |
| C | 2.28782 | 2.09495 | -2.27906 |
| C | 4.54766 | 1.78469 | -0.66127 |
| C | 3.43103 | 1.45446 | -2.75940 |
| C | 4.57104 | 1.28403 | -1.96434 |
| H | 5.42591 | 1.65466 | -0.02378 |
| H | 3.43048 | 1.07301 | -3.78449 |
| C | -2.29352 | -2.54420 | -0.92048 |
| C | -3.48339 | -2.53476 | -0.16536 |
| C | -2.26063 | -1.93604 | -2.19540 |
| C | -4.62282 | -1.92500 | -0.70079 |
| C | -3.42046 | -1.33371 | -2.68857 |
| C | -4.61490 | -1.32128 | -1.95991 |
| H | -5.54071 | -1.90884 | -0.10692 |
| H | -3.38992 | -0.86260 | -3.67533 |
| C | 2.44769 | -2.52547 | 0.91310 |
| C | 2.56730 | -2.14984 | 2.27103 |
| C | 3.50748 | -2.27974 | 0.01459 |
| C | 3.73065 | -1.50446 | 2.69495 |
| C | 4.66033 | -1.64243 | 0.48683 |
| C | 4.78624 | -1.23360 | 1.81577 |
| H | 3.81512 | -1.20470 | 3.74324 |
| H | 5.47573 | -1.44420 | -0.21307 |
| C | -3.52614 | -3.11780 | 1.22276 |
| H | -4.50735 | -2.94839 | 1.68560 |
| H | -3.32898 | -4.19968 | 1.22456 |
| H | -2.76004 | -2.65949 | 1.86769 |
| C | -0.99521 | -1.91154 | -3.00945 |
| H | -0.52497 | -2.90458 | -3.05593 |
| H | -1.19498 | -1.57045 | -4.03367 |
| H | -0.25491 | -1.23038 | -2.56061 |
| C | -5.85565 | -0.68507 | -2.52672 |
| H | -5.64477 | 0.31592 | -2.93244 |
| H | -6.26885 | -1.28507 | -3.35292 |
| H | -6.64034 | -0.58778 | -1.76394 |
| C | 3.38910 | -2.63384 | -1.44295 |
| H | 4.36841 | -2.58747 | -1.93723 |
| H | 2.96605 | -3.63622 | -1.59267 |
| H | 2.72044 | -1.92010 | -1.95335 |
| C | 1.45440 | -2.43490 | 3.24239 |
| H | 0.54026 | -1.88656 | 2.96867 |
| H | 1.19350 | -3.50375 | 3.24899 |
| H | 1.73677 | -2.13686 | 4.26040 |
| C | 6.01358 | -0.50382 | 2.29101 |
| H | 5.78451 | 0.55303 | 2.50268 |
| H | 6.40724 | -0.93915 | 3.22132 |
| H | 6.81170 | -0.52855 | 1.53687 |
| C | 5.78482 | 0.57843 | -2.50592 |
| H | 6.58307 | 0.52086 | -1.75384 |
| H | 5.53935 | -0.44737 | -2.82137 |
| H | 6.18868 | 1.09772 | -3.38852 |
| C | -6.02003 | 0.43444 | 2.13637 |
| H | -6.80982 | 0.48302 | 1.37453 |
| H | -5.77987 | -0.62895 | 2.29788 |
| H | -6.42644 | 0.82172 | 3.08165 |
| C | -1.64658 | 2.65476 | 3.29850 |
| H | -1.49183 | 3.74400 | 3.30432 |
| H | -1.96774 | 2.34453 | 4.30122 |
| H | -0.66682 | 2.19796 | 3.09409 |
| C | 1.06952 | 2.23043 | -3.15089 |

```
H          0.70038        3.26612       -3.17154
H          1.28570        1.91411       -4.17955
H          0.24611        1.60728       -2.76653
C          3.42392        2.89932        1.29476
H          4.39789        2.70975        1.76462
H          3.20132        3.97258        1.37792
H          2.65471        2.37055        1.87940
C         -3.19505        2.41840       -1.52919
H         -4.12685        2.29399       -2.09599
H         -2.79978        3.42708       -1.71223
H         -2.46078        1.70263       -1.93421
H         -0.12697        0.16250        1.98900
H          0.85495        0.21241        1.78120
```

### 3.2.13 $[TerN]_2PAs\cdot H_2$_TS (PBE-D3/def2-TZVP)

```
104
[TerN]2PAs·H2_TS PBE-D3/def2-TZVP
N         -0.02188       -1.14764        0.11198
N          0.08452        1.16760       -0.02247
P          1.31008       -0.03477       -0.14188
C          0.18942        2.55784        0.04071
C          1.38484        3.22815       -0.32501
C         -0.91296        3.32090        0.50748
C          1.45799        4.62071       -0.20545
C         -0.80080        4.71199        0.59966
C          0.37747        5.37082        0.25408
H          2.38828        5.11372       -0.49561
H         -1.66022        5.27599        0.96815
H          0.45195        6.45553        0.33953
C          0.01277       -2.54413        0.04706
C          1.17723       -3.25563        0.44332
C         -1.12560       -3.28406       -0.36570
C          1.18673       -4.65333        0.38722
C         -1.07536       -4.68163       -0.40278
C          0.07394       -5.37727       -0.03549
H          2.09445       -5.17219        0.70281
H         -1.96822       -5.22105       -0.72628
H          0.09823       -6.46706       -0.06624
C         -2.39717       -2.61087       -0.75197
C         -3.35759       -2.30111        0.23980
C         -2.67164       -2.33779       -2.11300
C         -4.56409       -1.71134       -0.14489
C         -3.88223       -1.72138       -2.44756
C         -4.83720       -1.39399       -1.47890
H         -5.30257       -1.47311        0.62348
H         -4.08570       -1.49541       -3.49778
C          2.39080       -2.55917        0.95490
C          3.53505       -2.43164        0.14026
C          2.40114       -2.05277        2.27427
C          4.66868       -1.79703        0.65935
C          3.55195       -1.42043        2.74842
C          4.69876       -1.28307        1.95718
H          5.54983       -1.68827        0.02159
H          3.55419       -1.02520        3.76839
C         -2.19327        2.67435        0.91068
```

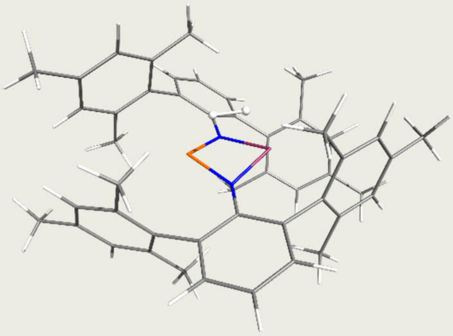

| | | | |
|---|---|---|---|
| C | -3.31929 | 2.75842 | 0.06455 |
| C | -2.28756 | 1.99417 | 2.14577 |
| C | -4.52065 | 2.16665 | 0.47093 |
| C | -3.50648 | 1.41683 | 2.51041 |
| C | -4.63782 | 1.49636 | 1.69077 |
| H | -5.38916 | 2.22748 | -0.19083 |
| H | -3.57383 | 0.88818 | 3.46553 |
| C | 2.56258 | 2.49303 | -0.86874 |
| C | 2.59231 | 2.15726 | -2.24236 |
| C | 3.66434 | 2.18750 | -0.04202 |
| C | 3.70729 | 1.48756 | -2.75191 |
| C | 4.76751 | 1.53335 | -0.59950 |
| C | 4.80260 | 1.15904 | -1.94468 |
| H | 3.72289 | 1.22006 | -3.81218 |
| H | 5.61641 | 1.29186 | 0.04485 |
| C | -3.23511 | 3.44456 | -1.27461 |
| H | -4.11825 | 3.21475 | -1.88532 |
| H | -3.17146 | 4.53817 | -1.16803 |
| H | -2.33734 | 3.13606 | -1.83043 |
| C | -1.09014 | 1.86720 | 3.04673 |
| H | -0.60805 | 2.84121 | 3.21478 |
| H | -1.37276 | 1.44253 | 4.01877 |
| H | -0.33083 | 1.21169 | 2.59146 |
| C | -5.95069 | 0.90504 | 2.12847 |
| H | -5.80434 | -0.02676 | 2.69363 |
| H | -6.49532 | 1.60013 | 2.78766 |
| H | -6.60142 | 0.69123 | 1.26935 |
| C | 3.63358 | 2.48000 | 1.43261 |
| H | 4.64352 | 2.44037 | 1.86149 |
| H | 3.19334 | 3.46104 | 1.65360 |
| H | 3.01961 | 1.72252 | 1.94930 |
| C | 1.44060 | 2.51618 | -3.14201 |
| H | 0.52172 | 1.99021 | -2.84096 |
| H | 1.21967 | 3.59313 | -3.09622 |
| H | 1.65880 | 2.24686 | -4.18359 |
| C | 5.97469 | 0.40203 | -2.50847 |
| H | 5.72692 | -0.66426 | -2.63609 |
| H | 6.26585 | 0.78531 | -3.49716 |
| H | 6.84808 | 0.46280 | -1.84504 |
| C | 5.93084 | -0.61097 | 2.50027 |
| H | 5.69141 | 0.37027 | 2.93663 |
| H | 6.39424 | -1.21338 | 3.29736 |
| H | 6.68427 | -0.46173 | 1.71506 |
| C | -6.11123 | -0.68995 | -1.85811 |
| H | -5.96903 | 0.40274 | -1.83533 |
| H | -6.43433 | -0.95720 | -2.87364 |
| H | -6.92577 | -0.92874 | -1.16040 |
| C | -1.67764 | -2.69606 | -3.18384 |
| H | -1.49111 | -3.78009 | -3.20803 |
| H | -2.03743 | -2.38225 | -4.17211 |
| H | -0.70674 | -2.21270 | -3.00040 |
| C | 1.18121 | -2.16103 | 3.14792 |
| H | 0.79604 | -3.19071 | 3.17835 |
| H | 1.40282 | -1.83889 | 4.17369 |
| H | 0.36782 | -1.52845 | 2.75734 |
| C | 3.52764 | -2.89991 | -1.29097 |
| H | 4.52080 | -2.78138 | -1.74385 |
| H | 3.22709 | -3.95286 | -1.38198 |
| H | 2.81082 | -2.31366 | -1.88845 |
| C | -3.07266 | -2.57132 | 1.69150 |

```
H          -3.95073       -2.34773        2.31093
H          -2.77708       -3.61692        1.85928
H          -2.23887       -1.94403        2.04551
As         -1.38630        0.03876       -0.48507
H           0.78264       -0.16017       -1.80204
H          -0.23154       -0.10124       -1.99949
```

### 3.2.14 [TerNAs]$_2$·$H_2$_TS (PBE-D3/def2-TZVP)

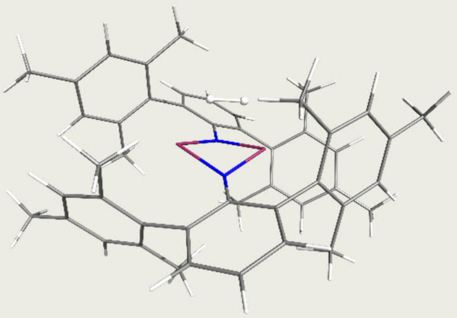

```
104
[TerNAs]₂·H₂_TS PBE-D3/def2-TZVP
N          -0.06779        1.19619       -0.03513
N           0.06787       -1.19622       -0.03524
C           0.12631       -2.58611       -0.05896
C           1.30517       -3.29047        0.30762
C          -0.99791       -3.33867       -0.50127
C           1.33618       -4.68704        0.23632
C          -0.92787       -4.73426       -0.54779
C           0.22931       -5.42079       -0.18506
H           2.25724       -5.19680        0.52785
H          -1.80611       -5.28174       -0.89693
H           0.26952       -6.50935       -0.23497
C          -0.12627        2.58608       -0.05891
C           0.99795        3.33864       -0.50122
C          -1.30515        3.29041        0.30765
C           0.92788        4.73423       -0.54778
C          -1.33618        4.68698        0.23634
C          -0.22932        5.42074       -0.18505
H           1.80610        5.28172       -0.89694
H          -2.25725        5.19673        0.52786
H          -0.26954        6.50929       -0.23498
C          -2.52451        2.57541        0.77882
C          -3.55979        2.26305       -0.13064
C          -2.66523        2.25427        2.14969
C          -4.70583        1.61742        0.34214
C          -3.81941        1.58854        2.57368
C          -4.84620        1.25256        1.68393
H          -5.50118        1.37025       -0.36438
H          -3.91907        1.32754        3.63088
C           2.25599        2.67199       -0.93909
C           3.39303        2.68928       -0.10384
C           2.31641        2.03633       -2.20060
C           4.56957        2.07162       -0.54391
C           3.51046        1.43104       -2.59844
C           4.65108        1.44054       -1.78686
H           5.44474        2.07406        0.11155
H           3.55113        0.93665       -3.57328
C          -2.25595       -2.67202       -0.93914
C          -3.39300       -2.68935       -0.10390
C          -2.31636       -2.03630       -2.20063
C          -4.56953       -2.07164       -0.54394
C          -3.51040       -1.43096       -2.59843
C          -4.65101       -1.44047       -1.78684
H          -5.44471       -2.07414        0.11149
H          -3.55106       -0.93652       -3.57324
C           2.52453       -2.57549        0.77881
```

| | | | |
|---|---|---|---|
| C | 2.66529 | -2.25441 | 2.14970 |
| C | 3.55973 | -2.26297 | -0.13068 |
| C | 3.81939 | -1.58854 | 2.57366 |
| C | 4.70570 | -1.61720 | 0.34207 |
| C | 4.84608 | -1.25235 | 1.68385 |
| H | 3.91906 | -1.32756 | 3.63086 |
| H | 5.50097 | -1.36987 | -0.36449 |
| C | -3.34326 | -3.32486 | 1.26143 |
| H | -4.23968 | -3.06940 | 1.84198 |
| H | -3.27960 | -4.42166 | 1.19846 |
| H | -2.45808 | -2.99554 | 1.82580 |
| C | -1.10614 | -1.98412 | -3.09188 |
| H | -0.66553 | -2.98217 | -3.23044 |
| H | -1.36051 | -1.57179 | -4.07695 |
| H | -0.32464 | -1.35062 | -2.64249 |
| C | -5.93323 | -0.80795 | -2.25639 |
| H | -5.75283 | 0.17873 | -2.70808 |
| H | -6.42196 | -1.42842 | -3.02439 |
| H | -6.64544 | -0.68394 | -1.42933 |
| C | 3.41062 | -2.56879 | -1.59580 |
| H | 4.33374 | -2.33322 | -2.14102 |
| H | 3.15680 | -3.62382 | -1.77032 |
| H | 2.59577 | -1.96914 | -2.03345 |
| C | 1.58748 | -2.61967 | 3.13386 |
| H | 0.63745 | -2.12337 | 2.88468 |
| H | 1.39108 | -3.70219 | 3.12376 |
| H | 1.87011 | -2.32341 | 4.15225 |
| C | 6.05627 | -0.49364 | 2.15707 |
| H | 6.41371 | -0.86691 | 3.12748 |
| H | 6.88118 | -0.56338 | 1.43517 |
| H | 5.81898 | 0.57455 | 2.28818 |
| C | 5.93332 | 0.80811 | -2.25646 |
| H | 5.75297 | -0.17858 | -2.70818 |
| H | 6.42199 | 1.42863 | -3.02446 |
| H | 6.64557 | 0.68411 | -1.42943 |
| C | -6.05643 | 0.49391 | 2.15713 |
| H | -6.41186 | 0.86493 | 3.12913 |
| H | -6.88244 | 0.56642 | 1.43677 |
| H | -5.82006 | -0.57489 | 2.28493 |
| C | -1.58737 | 2.61943 | 3.13382 |
| H | -1.39220 | 3.70220 | 3.12503 |
| H | -1.86923 | 2.32160 | 4.15197 |
| H | -0.63689 | 2.12454 | 2.88360 |
| C | 1.10619 | 1.98418 | -3.09186 |
| H | 0.66584 | 2.98231 | -3.23073 |
| H | 1.36048 | 1.57149 | -4.07679 |
| H | 0.32451 | 1.35103 | -2.64229 |
| C | 3.34324 | 3.32466 | 1.26155 |
| H | 4.24011 | 3.06995 | 1.84175 |
| H | 3.27855 | 4.42140 | 1.19869 |
| H | 2.45858 | 2.99449 | 1.82623 |
| C | -3.41072 | 2.56890 | -1.59576 |
| H | -4.33394 | 2.33362 | -2.14091 |
| H | -3.15659 | 3.62386 | -1.77026 |
| H | -2.59608 | 1.96901 | -2.03349 |
| As | -1.41931 | -0.06107 | 0.35600 |
| As | 1.41929 | 0.06102 | 0.35631 |
| H | -0.52997 | -0.04657 | 1.96703 |
| H | 0.52953 | 0.04633 | 1.96717 |

### 3.2.15 [TerNP]$_2$DmpNC·$H_2$_TS (PBE-D3/def2-TZVP)

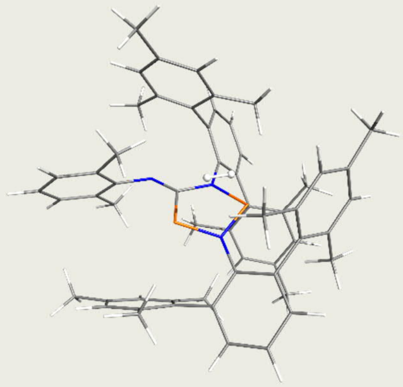

```
123
[TerNP]2DmpNC·H2_TS PBE-D3/def2-TZVP
C          4.70661        1.96694        4.11681
C          3.90078       -0.26293        3.23966
C          3.26419       -2.51473        2.32313
C          4.97893       -0.13563       -0.24621
C          3.94678       -4.59282       -2.36060
C          3.60888        0.99817        3.76855
C          4.45882       -2.31531       -1.39295
C          2.89568       -1.16552        2.87821
C          4.26470       -0.93314       -1.30312
C          3.78085       -3.09772       -2.33325
C          2.26517        1.34289        3.93648
C          0.27799       -2.73737        3.81603
C          1.54460       -0.78253        3.03321
C         -0.69214       -3.72741        3.71293
C          1.22642        0.48124        3.56964
C          2.92396       -2.45472       -3.23356
C          3.34862       -0.31687       -2.18233
C          0.46406       -1.78713        2.80025
C          4.02735        2.02825       -2.64305
C          1.41439        3.34602        1.38060
C          2.69862       -1.07683       -3.18074
C          3.08729        1.14787       -2.09326
C          3.82188        3.40479       -2.63435
C         -1.46509       -3.79924        2.55810
C         -0.20701        0.89111        3.75614
C         -0.35803       -1.84139        1.65119
C          0.01890       -4.06575       -0.81510
C          1.91049        1.67111       -1.50670
C          2.65190        3.91078       -2.07586
C         -1.30533       -2.88525        1.50825
C          0.32161        3.81540        0.46487
C          1.68300        3.07165       -1.50949
C          1.78057       -0.41589       -4.17283
C         -0.85620        4.35697        0.98911
C          0.45748        3.69782       -0.93251
C         -1.44295       -3.70405       -0.85350
C         -2.09360       -3.14796        0.27087
C         -1.26948       -0.05156        0.15746
C         -1.89564        4.79967        0.16645
C         -2.20087       -4.02383       -1.98607
C         -0.55362        4.19310       -1.78596
C         -3.18631        5.30734        0.74706
C         -3.49875       -3.00947        0.27516
C         -4.24431       -2.52190        1.48902
C         -1.71119        4.72988       -1.21929
C         -0.43927        4.06384       -3.28047
C         -3.58700       -3.84750       -2.02274
C         -4.21569       -3.36050       -0.87382
C         -4.37462       -4.14703       -3.26930
H          4.94548        2.61375        3.25677
H          4.94525       -0.55924        3.10967
```

| | | | |
|---|---|---|---|
| H | 4.35473 | -2.63552 | 2.28532 |
| H | 5.64706 | -0.77874 | 0.34082 |
| H | 4.92018 | -4.89816 | -1.95315 |
| H | 5.14931 | -2.79647 | -0.69509 |
| H | 5.63092 | 1.44411 | 4.39907 |
| H | 4.41393 | 2.62483 | 4.94690 |
| H | 2.84530 | -3.33429 | 2.92463 |
| H | 3.85772 | -4.98961 | -3.38147 |
| H | 5.57569 | 0.67914 | -0.68075 |
| H | 4.26307 | 0.32936 | 0.45016 |
| H | 2.87338 | -2.64074 | 1.30048 |
| H | 3.16792 | -5.08062 | -1.75192 |
| H | 0.91190 | -2.67348 | 4.70229 |
| H | -0.83229 | -4.45031 | 4.51800 |
| H | 2.01355 | 2.31682 | 4.36454 |
| H | 4.92530 | 1.60512 | -3.09726 |
| H | 1.21812 | 3.65608 | 2.41416 |
| H | 2.39488 | 3.74340 | 1.07843 |
| H | 2.40843 | -3.04426 | -3.99651 |
| H | 4.56412 | 4.07740 | -3.06627 |
| H | 1.50235 | 2.24974 | 1.37621 |
| H | -0.74053 | 0.19787 | 4.42435 |
| H | 0.67347 | -3.19171 | -0.68332 |
| H | 0.23039 | -4.74958 | 0.02142 |
| H | -2.20098 | -4.59574 | 2.43675 |
| H | -0.27666 | 1.90103 | 4.18089 |
| H | 0.31981 | -4.55774 | -1.74965 |
| H | 1.42206 | -1.13964 | -4.91590 |
| H | -0.96418 | 4.43108 | 2.07497 |
| H | -0.75496 | 0.87938 | 2.80309 |
| H | 2.46963 | 4.98675 | -2.05836 |
| H | 0.90058 | 0.02425 | -3.68014 |
| H | 2.29044 | 0.40448 | -4.69996 |
| H | -3.06517 | 5.60279 | 1.79821 |
| H | -4.49743 | -3.35861 | 2.16017 |
| H | -1.69148 | -4.44803 | -2.85588 |
| H | -3.64579 | -1.79887 | 2.05472 |
| H | -3.95702 | 4.52096 | 0.70635 |
| H | 0.44660 | 4.58038 | -3.67711 |
| H | -0.34337 | 3.00515 | -3.57319 |
| H | -3.56880 | 6.17153 | 0.18524 |
| H | -5.18445 | -2.03671 | 1.19431 |
| H | -2.50854 | 5.07811 | -1.88125 |
| H | -1.32889 | 4.47347 | -3.77575 |
| H | -5.30126 | -3.22881 | -0.87306 |
| H | -3.89763 | -4.93525 | -3.86839 |
| H | -5.39923 | -4.46493 | -3.03126 |
| H | -4.45052 | -3.25156 | -3.90800 |
| N | 0.93898 | 0.80490 | -0.90194 |
| N | -0.20107 | -0.84186 | 0.62541 |
| P | 1.32904 | -0.67810 | -0.15248 |
| P | -0.75247 | 1.01306 | -1.23103 |
| C | -4.05957 | 0.58510 | -1.01071 |
| C | -4.18939 | 1.44908 | 1.28403 |
| C | -5.21571 | 1.32068 | -1.31180 |
| C | -5.33827 | 2.15826 | 0.93670 |
| C | -5.85518 | 2.10695 | -0.36020 |
| H | -5.62487 | 1.25134 | -2.32308 |
| H | -5.83691 | 2.75951 | 1.70124 |
| H | -6.75909 | 2.66120 | -0.61766 |

```
C          -3.52182         0.66420         0.30206
N          -2.42291        -0.07637         0.73600
C          -3.63356         1.53318         2.67579
H          -3.35544         0.53814         3.05256
H          -2.71455         2.14022         2.69836
H          -4.35992         1.99080         3.36098
C          -3.46743        -0.28679        -2.08447
H          -2.79581         0.28269        -2.75097
H          -2.88998        -1.12151        -1.67301
H          -4.26477        -0.70462        -2.71559
H          -0.36457        -0.85804        -1.95496
H           0.32504        -1.33024        -1.69689
```

### 3.2.16 [TerN]$_2$PAsDmpNC·$H_2$_TS (PBE-D3/def2-TZVP)

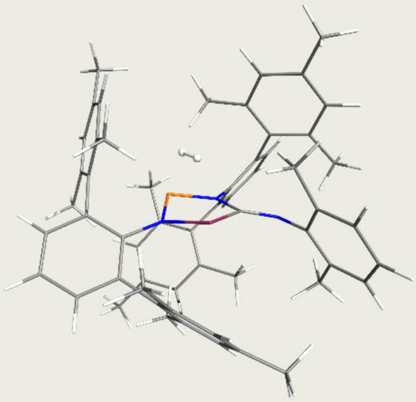

```
123
[TerN]₂PAsDmpNC·H₂_TS PBE-D3/def2-TZVP
As          0.79067         1.17413        -1.17153
N           0.23308        -0.89509         0.63279
N          -1.03085         0.81096        -0.79377
N           2.44176        -0.12039         0.85513
C           1.30566        -0.07564         0.24703
P          -1.30730        -0.72434        -0.13113
C           0.38559        -1.96119         1.59631
C          -2.07561         1.64057        -1.30662
C           3.53227         0.66266         0.47690
C           1.33373        -2.99397         1.39060
C          -0.43978        -1.98049         2.74442
C          -1.94306         3.05409        -1.23942
C          -3.24697         1.07965        -1.87507
C           4.16981         1.41188         1.50618
C           4.07945         0.67646        -0.83479
C           1.48691        -3.97622         2.37800
C           2.13932        -3.17190         0.14874
C          -0.25639        -2.99436         3.69765
C          -1.52298        -0.99548         3.04152
C          -3.00230         3.85824        -1.67913
C          -0.70137         3.72402        -0.75559
C          -4.27726         1.92626        -2.30319
C          -3.40805        -0.38737        -2.08447
C           5.29073         2.18371         1.20542
C           3.61241         1.39253         2.89976
C           5.20817         1.47181        -1.08754
C           3.53761        -0.16373        -1.96053
H           2.22272        -4.76373         2.20749
C           0.71082        -3.97844         3.53276
C           1.50916        -3.65711        -1.01901
C           3.54361        -3.02851         0.18360
H          -0.89224        -2.98631         4.58479
C          -2.87365        -1.37712         2.87693
C          -1.20749         0.23657         3.64843
H          -2.88844         4.94108        -1.60244
C          -4.17174         3.30966        -2.19706
C          -0.39900         3.76255         0.62180
C           0.17863         4.31758        -1.68833
H          -5.16658         1.47190        -2.74405
```

| | | | |
|---|---|---|---|
| C | -2.71727 | -1.01482 | -3.14555 |
| C | -4.27817 | -1.13615 | -1.26338 |
| H | 5.76253 | 2.76040 | 2.00495 |
| C | 5.81270 | 2.22720 | -0.09012 |
| H | 4.32811 | 1.82276 | 3.61327 |
| H | 3.35890 | 0.36904 | 3.21130 |
| H | 2.67922 | 1.97416 | 2.96148 |
| H | 5.62421 | 1.47408 | -2.09838 |
| H | 4.36510 | -0.56893 | -2.56060 |
| H | 2.90791 | 0.42554 | -2.65148 |
| H | 2.93614 | -1.00606 | -1.60397 |
| H | 0.84707 | -4.75260 | 4.28939 |
| C | 2.28551 | -3.89819 | -2.15853 |
| C | 0.05124 | -4.03464 | -1.02463 |
| C | 4.28003 | -3.30279 | -0.97405 |
| C | 4.26811 | -2.61790 | 1.43816 |
| C | -3.87998 | -0.50018 | 3.29367 |
| C | -3.24012 | -2.69597 | 2.25229 |
| C | -2.24730 | 1.07466 | 4.06388 |
| C | 0.22512 | 0.63407 | 3.86282 |
| H | -4.98541 | 3.95468 | -2.53123 |
| C | 0.81637 | 4.31425 | 1.03533 |
| C | -1.34983 | 3.18255 | 1.62650 |
| C | 1.38104 | 4.86191 | -1.22941 |
| C | -0.11318 | 4.28575 | -3.16527 |
| C | -2.85491 | -2.39496 | -3.31845 |
| C | -1.85313 | -0.21334 | -4.08079 |
| C | -4.38326 | -2.51478 | -1.47171 |
| C | -5.04239 | -0.47850 | -0.14715 |
| H | 6.69396 | 2.83137 | -0.31125 |
| H | 1.79101 | -4.26514 | -3.06227 |
| C | 3.67134 | -3.71566 | -2.16201 |
| H | -0.14431 | -4.83542 | -0.29402 |
| H | -0.25330 | -4.39082 | -2.01766 |
| H | -0.61147 | -3.19825 | -0.76094 |
| H | 5.36506 | -3.16898 | -0.94723 |
| H | 5.21642 | -2.12217 | 1.19081 |
| H | 4.50403 | -3.49416 | 2.06349 |
| H | 3.66202 | -1.92640 | 2.03464 |
| H | -4.92397 | -0.79503 | 3.15712 |
| C | -3.59033 | 0.73371 | 3.88459 |
| H | -4.32943 | -2.83123 | 2.23901 |
| H | -2.79232 | -3.54378 | 2.79004 |
| H | -2.87766 | -2.75255 | 1.21303 |
| H | -1.99710 | 2.02548 | 4.54195 |
| H | 0.29417 | 1.62699 | 4.32608 |
| H | 0.78359 | 0.65319 | 2.91644 |
| H | 0.74838 | -0.08529 | 4.51159 |
| H | 1.05745 | 4.31781 | 2.10209 |
| C | 1.73629 | 4.84186 | 0.12367 |
| H | -1.03928 | 3.43134 | 2.64861 |
| H | -1.39283 | 2.08627 | 1.55002 |
| H | -2.37579 | 3.54919 | 1.47402 |
| H | 2.07909 | 5.28458 | -1.95694 |
| H | 0.72697 | 4.70257 | -3.73537 |
| H | -1.01853 | 4.85506 | -3.42157 |
| H | -0.28562 | 3.25451 | -3.51319 |
| H | -2.30917 | -2.88097 | -4.13175 |
| C | -3.66293 | -3.16758 | -2.47768 |
| H | -1.48154 | -0.83946 | -4.90212 |

```
H          -0.98025         0.21140        -3.56189
H          -2.41085         0.63301        -4.50895
H          -5.03716        -3.09804        -0.81775
H          -5.60637        -1.22295         0.42919
H          -5.75151         0.27294        -0.52416
H          -4.36353         0.04432         0.54424
C           4.47926        -3.93102        -3.41303
C          -4.69062         1.67757         4.28862
C           3.08185         5.33460         0.57878
C          -3.73513        -4.66212        -2.63528
H           5.50378        -4.25277        -3.17987
H           4.55435        -2.99804        -3.99573
H           4.01910        -4.68616        -4.06554
H          -4.95683         2.34828         3.45535
H          -4.38650         2.31178         5.13282
H          -5.60243         1.13485         4.57436
H           3.46698         6.12414        -0.08128
H           3.81153         4.50848         0.56984
H           3.04120         5.72691         1.60453
H          -2.95050        -5.15404        -2.03740
H          -3.58575        -4.96460        -3.68102
H          -4.70184        -5.05753        -2.29440
H           0.36453        -0.79496        -1.98996
H          -0.29862        -1.27840        -1.72830
```

### 3.2.17 $H_2$ (PBE-D3/def2-TZVP)

```
2
H₂ PBE-D3/def2-TZVP
H           0.00000         0.00000         0.37573
H           0.00000         0.00000        -0.37573
```

### 3.2.18 $[TerNAs]_2DmpNC$ (PBE-D3/def2-TZVP)

```
121
[TerNAs]₂DmpNC PBE-D3/def2-TZVP
As         -0.00000         0.00000         1.87090
N          -0.76972         0.15099        -0.85911
N           1.39349        -0.11410         0.62713
N          -2.58060         0.31189         0.61426
C          -1.31066         0.13615         0.44424
As          1.05793        -0.00000        -1.15674
C          -1.62603         0.36272        -1.99626
C           2.72926        -0.33737         1.09788
C          -3.19944         0.16742         1.85498
C          -2.68855        -0.52991        -2.27996
C          -1.38810         1.47282        -2.83943
C           3.34808         0.62936         1.92795
C           3.41318        -1.53046         0.76371
C          -4.03061         1.23721         2.29218
C          -3.13475        -1.02652         2.62770
C          -3.52929        -0.24833        -3.36509
C          -2.92983        -1.81114        -1.55869
```

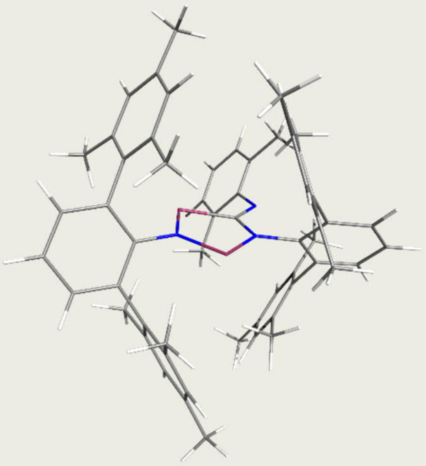

| | | | |
|---|---|---|---|
| C | -2.25049 | 1.70341 | -3.92223 |
| C | -0.26192 | 2.44102 | -2.67389 |
| C | 4.62873 | 0.37207 | 2.43131 |
| C | 2.70054 | 1.93504 | 2.24151 |
| C | 4.69948 | -1.73798 | 1.28339 |
| C | 2.84659 | -2.60385 | -0.10666 |
| C | -4.73281 | 1.12168 | 3.49076 |
| C | -4.13448 | 2.48637 | 1.46428 |
| C | -3.86113 | -1.09216 | 3.82601 |
| C | -2.35664 | -2.24164 | 2.20083 |
| H | -4.34222 | -0.94402 | -3.57771 |
| C | -3.32702 | 0.86238 | -4.17864 |
| C | -2.01332 | -2.87480 | -1.70040 |
| C | -4.14327 | -2.01674 | -0.86030 |
| H | -2.06236 | 2.57112 | -4.55698 |
| C | 0.83211 | 2.38668 | -3.56823 |
| C | -0.35616 | 3.49341 | -1.73906 |
| H | 5.09620 | 1.12731 | 3.06554 |
| C | 5.30579 | -0.80370 | 2.11760 |
| C | 2.74147 | 2.97152 | 1.28815 |
| C | 2.09293 | 2.14791 | 3.49631 |
| H | 5.21546 | -2.66488 | 1.02633 |
| C | 2.00376 | -3.59336 | 0.44540 |
| C | 3.25343 | -2.69381 | -1.45334 |
| H | -5.36033 | 1.95527 | 3.81595 |
| C | -4.64904 | -0.03532 | 4.26866 |
| H | -4.95720 | 3.12219 | 1.81853 |
| H | -4.29472 | 2.24539 | 0.40309 |
| H | -3.20567 | 3.07592 | 1.50854 |
| H | -3.81110 | -2.01442 | 4.41103 |
| H | -2.90828 | -3.15514 | 2.46617 |
| H | -1.37600 | -2.29240 | 2.70555 |
| H | -2.16665 | -2.26013 | 1.12229 |
| H | -3.99447 | 1.06022 | -5.01871 |
| C | -2.29926 | -4.10587 | -1.09454 |
| C | -0.76439 | -2.74579 | -2.52705 |
| C | -4.38706 | -3.26447 | -0.28070 |
| C | -5.17974 | -0.93218 | -0.72890 |
| C | 1.81155 | 3.38288 | -3.50624 |
| C | 0.95418 | 1.28229 | -4.58616 |
| C | 0.63879 | 4.47603 | -1.72453 |
| C | -1.52346 | 3.58470 | -0.79872 |
| H | 6.30408 | -0.98861 | 2.51695 |
| C | 2.11724 | 4.18735 | 1.58618 |
| C | 3.45171 | 2.79690 | -0.02731 |
| C | 1.46512 | 3.37273 | 3.74346 |
| C | 2.12066 | 1.09030 | 4.56841 |
| C | 1.57694 | -4.64717 | -0.36701 |
| C | 1.59107 | -3.54116 | 1.89081 |
| C | 2.79672 | -3.76518 | -2.23181 |
| C | 4.18711 | -1.67654 | -2.05724 |
| H | -5.20855 | -0.11605 | 5.20193 |
| H | -1.58178 | -4.92367 | -1.20728 |
| C | -3.47442 | -4.32074 | -0.37415 |
| H | -0.50471 | -3.70596 | -2.99127 |
| H | 0.10272 | -2.45035 | -1.91196 |
| H | -0.87466 | -1.99739 | -3.32288 |
| H | -5.31641 | -3.40845 | 0.27738 |
| H | -5.76079 | -1.06818 | 0.19304 |
| H | -5.88743 | -0.94977 | -1.57333 |

| | | | |
|---|---|---|---|
| H | -4.71038 | 0.05786 | -0.69827 |
| H | 2.66363 | 3.32743 | -4.18951 |
| C | 1.72614 | 4.44588 | -2.60150 |
| H | 1.95782 | 1.27334 | -5.03125 |
| H | 0.22251 | 1.40060 | -5.39992 |
| H | 0.76416 | 0.29232 | -4.14420 |
| H | 0.55346 | 5.29361 | -1.00434 |
| H | -1.39852 | 4.42296 | -0.10081 |
| H | -1.65180 | 2.66428 | -0.21432 |
| H | -2.46603 | 3.73099 | -1.34925 |
| H | 2.15551 | 4.99102 | 0.84666 |
| C | 1.44956 | 4.40021 | 2.79480 |
| H | 3.73040 | 3.77017 | -0.45034 |
| H | 2.80469 | 2.29912 | -0.76794 |
| H | 4.36023 | 2.18825 | 0.07891 |
| H | 0.96897 | 3.52470 | 4.70583 |
| H | 1.40054 | 1.32286 | 5.36353 |
| H | 3.11910 | 1.01531 | 5.02767 |
| H | 1.88013 | 0.09441 | 4.16971 |
| H | 0.92135 | -5.40818 | 0.06521 |
| C | 1.96414 | -4.75575 | -1.70738 |
| H | 1.00201 | -4.42657 | 2.16200 |
| H | 0.97892 | -2.64915 | 2.09933 |
| H | 2.46734 | -3.48830 | 2.55461 |
| H | 3.10336 | -3.82336 | -3.27971 |
| H | 4.20735 | -1.76678 | -3.15120 |
| H | 5.21585 | -1.80899 | -1.68732 |
| H | 3.89971 | -0.64546 | -1.80265 |
| C | -3.73927 | -5.63429 | 0.31048 |
| C | 2.76596 | 5.53377 | -2.58713 |
| C | 0.71744 | 5.68848 | 3.05860 |
| C | 1.49652 | -5.91062 | -2.55142 |
| H | -4.80499 | -5.90103 | 0.27330 |
| H | -3.45423 | -5.58584 | 1.37448 |
| H | -3.16271 | -6.45010 | -0.14718 |
| H | 2.79001 | 6.05499 | -1.62004 |
| H | 2.55658 | 6.28964 | -3.36108 |
| H | 3.77003 | 5.13382 | -2.78841 |
| H | 0.73550 | 5.95279 | 4.12508 |
| H | -0.34098 | 5.60321 | 2.76268 |
| H | 1.15149 | 6.52173 | 2.48859 |
| H | 1.73448 | -5.75458 | -3.61206 |
| H | 0.40943 | -6.05807 | -2.46375 |
| H | 1.97377 | -6.85156 | -2.23550 |

# 4 References


S1. V. V. Zhivonitko, K. Sorochkina, K. Chernichenko, B. Kotai, T. Foldes, I. Papai, V.-V. Telkki, T. Repo and I. Koptyug, Phys. Chem. Chem. Phys., 2016, 18, 27784-27795.

S2. S. Korchak, S. J. Yang, S. Mamone and S. Glöggler, Chemistryopen, 2018, 7, 344-348.

S3. S. Korchak, S. Mamone and S. Glöggler, Chemistryopen, 2018, 7, 672-676.

S4. C. R. Bowers, in Encyclopedia of Nuclear Magnetic Resonance, eds. D. M. Grant and R. K. Harris, Wiley, Chichester, 2002, vol. 9, ch. Chapter, pp. 750-769.

S5. V. V. Zhivonitko, J. Bresien, A. Schulz and I. V. Koptyug, Phys. Chem. Chem. Phys., 2019, 21, 5890-5893.

S6. S. Aime, R. Gobetto and D. Canet, J. Am. Chem. Soc., 1998, 120, 6770-6773.

S7. K. Sorochkina, V. V. Zhivonitko, K. Chernichenko, V. V. Telkki, T. Repo and I. V. Koptyug, J. Phys. Chem. Lett., 2018, 9, 903-907.

S8. H. Friebolin, Basic one- and two-dimensional NMR spectroscopy, WILEY-VCH, Weinheim, 5th edn., 2011.

S9. C. B. Fischer, S. J. Xu and H. Zipse, Chem. Eur. J., 2006, 12, 5779-5784.

S10. J. Bresien, T. Kröger-Badge, S. Lochbrunner, D. Michalik, H. Müller, A. Schulz and E. Zander, Chem. Sci., 2019, 10, 3486-3493.

S11. P. L. Bailey, R. A. Coxall, C. M. Dick, S. Fabre, L. C. Henderson, C. Herber, S. T. Liddle, D. Lorono-Gonzalez, A. Parkin and S. Parsons, Chem. Eur. J., 2003, 9, 4820-4828.

S12. S. Demeshko, C. Godemann, R. Kuzora, A. Schulz and A. Villinger, Angew. Chem. Int. Ed., 2013, 52, 2105-2108.

S13. H. Beer, J. Bresien, D. Michalik, A. Schulz and A. Villinger, Dalton Trans., 2020, 49, 13986-13992.

S14. B. A. Chalmers, M. Buhl, P. S. Nejman, A. M. Z. Slawin, J. D. Woollins and P. Kilian, J. Organomet. Chem., 2015, 799-800, 70-74.

S15. A. Hinz, A. Schulz and A. Villinger, Angew. Chem. Int. Ed., 2015, 54, 668-672.

S16. A. Hinz, A. Schulz and A. Villinger, Chem. Sci., 2016, 7, 745-751.

S17. M. J. Frisch, G. W. Trucks, H. B. Schlegel, G. E. Scuseria, M. A. Robb, J. R. Cheeseman, G. Scalmani, V. Barone, B. Mennucci, G. A. Petersson, H. Nakatsuji, M. Caricato, X. Li, H. P. Hratchian, A. F. Izmaylov, J. Bloino, G. Zheng, J. L. Sonnenberg, M. Hada, M. Ehara, K. Toyota, R. Fukuda, J. Hasegawa, M. Ishida, T. Nakajima, Y. Honda, O. Kitao, H. Nakai, T. Vreven, J. A. Montgomery Jr., J. E. Peralta, F. Ogliaro, M. Bearpark, J. J. Heyd, E. Brothers, K. N. Kudin, V. N. Staroverov, T. Keith, R. Kobayashi, J. Normand, K. Raghavachari, A. Rendell, J. C. Burant, S. S. Iyengar, J. Tomasi, M. Cossi, N. Rega, J. M. Millam, M. Klene, J. E. Knox, J. B. Cross, V. Bakken, C. Adamo, J. Jaramillo, R. Gomperts, R. E. Stratmann, O. Yazyev, A. J. Austin, R. Cammi, C. Pomelli, J. W. Ochterski, R. L. Martin, K. Morokuma, V. G. Zakrzewski, G. A. Voth, P. Salvador, J. J. Dannenberg, S.

Dapprich, A. D. Daniels, O. Farkas, J. B. Foresman, J. V. Ortiz, J. Cioslowski and D. J. Fox, Gaussian 09 Rev. D.01, 2013.
S18. F. Neese, Wiley Interdiscip. Rev. Comput. Mol. Sci., 2018, 8, 1327.
S19. J. P. Perdew, K. Burke and M. Ernzerhof, ACS Symp. Ser., 1996, 629, 453-462.
S20. J. P. Perdew, K. Burke and M. Ernzerhof, Phys. Rev. Lett., 1997, 78, 1396-1396.
S21. S. Grimme, J. Antony, S. Ehrlich and H. Krieg, J. Chem. Phys., 2010, 132, 154104.
S22. S. Grimme, S. Ehrlich and L. Goerigk, J. Comput. Chem., 2011, 32, 1456-1465.
S23. F. Weigend and R. Ahlrichs, Phys. Chem. Chem. Phys., 2005, 7, 3297-3305.
S24. F. London, J. Phys. Radium, 1937, 8, 397-409.
S25. R. McWeeny, Phys. Rev., 1962, 126, 1028-1034.
S26. R. Ditchfield, Mol. Phys., 1974, 27, 789-807.
S27. K. Wolinski, J. F. Hinton and P. Pulay, J. Am. Chem. Soc., 1990, 112, 8251-8260.
S28. J. R. Cheeseman, G. W. Trucks, T. A. Keith and M. J. Frisch, J. Chem. Phys., 1996, 104, 5497-5509.
S29. C. Adamo and V. Barone, J. Chem. Phys., 1999, 110, 6158-6170.
S30. C. J. Jameson, A. De Dios and A. K. Jameson, Chem. Phys. Lett., 1990, 167, 575-582.
S31. C. van Wüllen, Phys. Chem. Chem. Phys., 2000, 2, 2137-2144.
S32. D. G. Liakos, Y. Guo and F. Neese, J. Phys. Chem. A, 2020, 124, 90-100.
S33. C. Riplinger, P. Pinski, U. Becker, E. F. Valeev and F. Neese, J. Chem. Phys., 2016, 144, 024109.
S34. C. Riplinger and F. Neese, J. Chem. Phys., 2013, 138, 034106.
S35. D. G. Liakos, M. Sparta, M. K. Kesharwani, J. M. L. Martin and F. Neese, J. Chem. Theory Comput., 2015, 11, 1525-1539.
S36. A. Hellweg, C. Hättig, S. Höfener and W. Klopper, Theor. Chem. Acc., 2007, 117, 587-597.
S37. C. J. Cramer, Essentials of computational chemistry : theories and models, Wiley, Chichester, West Sussex, England ; Hoboken, NJ, 2nd edn., 2004.
